# Comparison of Adhesive Strategies with Different Etching Approaches on the Clinical Performance of Restorations in Non-Carious Cervical Lesions: A Systematic Review and Network Meta-Analysis

**DOI:** 10.3390/jfb17040160

**Published:** 2026-03-25

**Authors:** Alain Manuel Chaple Gil, Laura Pereda Vázquez, Meylin Santiesteban Velázquez, Jorge J. Menéndez

**Affiliations:** 1 Facultad de Ciencias de la Salud, Carrera de Odontología, Universidad Autónoma de Chile, Santiago 8900000, Chile; 2 Penn Dental Medicine, University of Pennsylvania, Philadelphia, PA 19104, USA; 3 Facultad de Odontología, Universidad Autónoma del Paraguay, Asunción 1255, Paraguay; 4 College of Dental Medicine, Nova Southeastern University, Davie, FL 33314, USA

**Keywords:** dental adhesives, composite resins, etching and conditioning agents, dental marginal adaptation, tooth cervical lesions, network meta-analysis as topic

## Abstract

Non-carious cervical lesions (NCCLs) present restorative challenges due to substrate sclerosis and marginal stress concentration, making the adhesive strategy a key determinant of long-term performance. This systematic review and frequentist random-effects network meta-analysis, conducted in accordance with PRISMA 2020 and the PRISMA-NMA extension and prospectively registered in PROSPERO, compared restorative strategies defined by etching approach, adhesive category, and restorative material, with marginal adaptation and retention loss as the primary outcomes. PubMed, Web of Science, Cochrane Library, Embase, and Scopus were searched without restrictions (25 January 2026), supplemented by alternative retrieval methods. Randomized clinical trials in permanent teeth evaluating at least two etching-based strategies (etch-and-rinse, self-etch, selective-etch, and/or resin-modified glass ionomer cement (RMGI)) were included. Risk of bias was assessed using RoB 2 and certainty of evidence with CINeMA. Seventy-four trials were eligible. Connected networks were established for marginal adaptation (57 studies; 28 interventions; 6798 patients; 1772 events) and retention loss (61 studies; 33 interventions; 7338 patients; 584 events). Selective-etch with a universal adhesive and nanocomposite reduced marginal adaptation failure compared with RMGI, whereas certain self-etch and non-universal combinations increased risk. For retention loss, selective-etch and etch-and-rinse protocols combined with universal adhesives and nanocomposites showed lower failure rates, while some self-etch or non-universal adhesive strategies performed less favorably. Overall confidence was predominantly high, with downgrading mainly due to imprecision and heterogeneity. Strategies incorporating selective enamel etching or etch-and-rinse approaches combined with universal adhesives and nanocomposites demonstrated the most consistent clinical advantages.

## 1. Introduction

Non-carious cervical lesions (NCCLs) are highly prevalent defects whose restoration remains clinically challenging due to substrate sclerosis, limited enamel at the cervical margin, and difficulties in isolation and stress distribution at the cemento-enamel junction [1,2,3,4]. In this context, the adhesive strategy is a key determinant of restoration longevity, particularly for outcomes such as retention, marginal adaptation, and fracture [2,3].

From an oral rehabilitation perspective, the durability of NCCL restorations is clinically relevant because marginal breakdown and restoration loss may compromise long-term comfort, increase hypersensitivity, and ultimately affect functional stability and maintenance needs. Therefore, identifying restorative protocols that maximize clinical longevity in NCCLs is directly aligned with rehabilitation-oriented decision-making in everyday practice [5,6,7].

Contemporary adhesive dentistry offers multiple clinically used approaches, most commonly the etch-and-rinse, self-etch, and selective-enamel-etching approaches combined with self-etch bonding [8,9]. These protocols differ in their interaction with enamel and dentin, technique sensitivity, and tolerance to moisture control, which may translate into differences in clinical performance over time [10,11]. Despite extensive clinical research, uncertainty persists regarding which strategy provides the most reliable clinical performance in NCCLs across follow-up periods [2,3].

Previous systematic reviews and clinical investigations have addressed adhesive performance in NCCLs; however, most comparative evidence has relied on direct or pairwise comparisons and therefore cannot simultaneously integrate all available direct and indirect evidence across multiple competing strategies [8]. Network meta-analysis (NMA) extends conventional meta-analysis by combining direct and indirect comparisons within a connected evidence network, enabling the estimation of relative effects across all interventions and the generation of probabilistic treatment hierarchies.

### 1.1. Research Question

Among patients with NCCLs restored with direct materials, what are the relative effects of all eligible restorative strategies (defined by etching mode, adhesive category, and restorative material) on marginal adaptation and retention loss?

### 1.2. Objective

Therefore, this systematic review and network meta-analysis aimed to estimate the relative clinical performance of all eligible restorative strategies for non-carious cervical lesion restorations, defined by the combination of etching approach, adhesive system, and restorative material, with respect to marginal adaptation and retention loss.

Accordingly, this study provides an updated synthesis of randomized clinical trials within a unified network framework, explicitly addressing key assumptions of transitivity and consistency, as well as major sources of uncertainty relevant to clinical translation.

## 2. Materials and Methods

The present systematic review and network meta-analysis was conducted in accordance with the Preferred Reporting Items for Systematic Reviews and Meta-Analyses (PRISMA 2020) guidelines [12], as well as the PRISMA extension for reporting network meta-analyses of health care interventions [13]. This review was prospectively registered in the International Prospective Register of Systematic Reviews (PROSPERO; registration number CRD420251158473).

### 2.1. Eligibility Criteria

The inclusion criteria for this review were defined using the PICO framework (Population, Intervention, Comparison, Outcome) to clearly delineate the clinical question and eligibility criteria.

Population (P): Human patients presenting with NCCLs in permanent teeth restored using direct adhesive composite restorations or resin-modified glass ionomer cements, applied according to clinically established protocols.

Intervention (I): Adhesive strategies involving etching agents and/or adhesive application protocols [14] with restorative material. Specifically:

• The etch-and-rinse approach, in which both enamel and dentin were conditioned with etching agent before adhesive application.
• The self-etch approach, in which no prior etching agent conditioning was performed, as the acidic monomers within the adhesive system provided substrate demineralization and bonding simultaneously.
• The selective-etch approach, in which etching agent was applied only to the enamel margins before using a self-etch adhesive on dentin.

Comparison (C): Alternative clinically relevant restorative strategies used for the management of NCCLs, including different adhesive protocols applied to composite resin restorations (etch-and-rinse, self-etch, and selective-etch strategies) as well as resin-modified glass ionomer cement (RMGI) restorations used as material-based comparators. Comparisons were therefore defined at the level of clinically applied restorative strategies, integrating both adhesive approach and restorative material when applicable.

To be eligible for inclusion, studies had to compare at least two distinct etching-based restorative strategies (etch-and-rinse, self-etch, selective-etch, or RMGI-based approaches). Trials in which all study arms employed the same etching protocol without variation were excluded, as they did not inform the comparative objective of the network meta-analysis.

Outcome (O): The primary outcomes were marginal adaptation and retention loss, assessed clinically using validated criteria such as USPHS [15], FDI [16], or comparable standardized indices as Vanherle et al. [17] Secondary outcomes were fracture of the restorations, anatomic form, marginal discoloration, postoperative sensitivity, secondary caries, and surface texture.

Randomized clinical trials evaluating NCCL restorations performed with resin-modified glass ionomer cements, either alone or in direct comparison with adhesive composite resin strategies, were considered eligible when clinical outcomes were assessed using standardized evaluation criteria.

The exclusion criteria were applied to ensure methodological coherence with the network meta-analysis’s comparative objective. Studies were excluded if they involved restorations placed on primary teeth or were conducted in vitro, in situ, or in animal models. Non-experimental designs, including observational studies, case reports, narrative reviews, short communications, and pilot trials with fewer than ten participants, were also excluded. In addition, clinical trials in which all study arms used the same etching protocol without comparing distinct etching-based restorative strategies were excluded, as they did not provide comparative information relevant to the network structure.

In addition to the aforementioned criteria, trials that introduced active co-interventions beyond the etching strategy or the restorative approach under evaluation were excluded. Specifically, studies assessing modified adhesive or restorative systems incorporating additional bioactive or functional components (e.g., nanoparticle-enriched formulations, antibacterial agents, or other additive modifications) were not eligible when the independent effect of such co-interventions could not be isolated from the etching protocol or restorative strategy. This exclusion was applied to preserve the transitivity assumption and ensure that observed differences in clinical outcomes could be attributed primarily to the restorative strategies compared within the network meta-analysis.

### 2.2. Search Strategy

The literature search was conducted on 25 January 2026, across PubMed, Web of Science, the Cochrane Library, Embase, and Scopus, without language or time restrictions. The search strategy was primarily designed to identify clinical studies evaluating adhesive restorations for NCCLs under different acid-etching modalities, including etch-and-rinse, selective-etch, and self-etch. Accordingly, combinations of NCCL-related terms and keywords describing adhesive systems and etching protocols were systematically incorporated into the search strings. Search alerts were established to identify newly published studies for future updates.

To ensure comprehensive retrieval of clinically relevant comparators, the search strategy also incorporated terms related to glass ionomer-based materials (e.g., “glass ionomer,” “resin-modified glass ionomer,” “RMGIC,” “RMGI”), allowing identification of trials in which adhesive strategies were compared with non-adhesive or alternative restorative approaches. Randomized clinical trials were identified by explicitly including trial-related terms within the search strings, rather than by applying database-specific methodological filters.

In addition to the primary search, an alternative identification approach was implemented using a more restrictive search formulation, in which selected terms and operators that could disproportionately broaden retrieval were intentionally removed. This complementary strategy aimed to identify potentially eligible studies that were more directly aligned with the review question but may have been obscured within the expanded results of the primary search. Furthermore, a supplementary retrieval step was conducted to identify eligible randomized or prospective clinical trials that met the inclusion criteria but were not retrieved by the primary search due to inconsistent reporting of study design terminology in titles or abstracts. All records identified through these alternative and supplementary approaches underwent the same independent screening and eligibility assessment as those retrieved through the primary database searches. The complete database-specific search strategies are provided in Supplementary File S1.

### 2.3. Study Selection

All bibliographic records retrieved from the search were imported into the Rayyan^®^ online platform [18], where duplicate entries were systematically removed.

Reviewer calibration was performed in two separate stages: (1) study selection and (2) data extraction. At each stage, Cohen’s kappa was used to assess inter-rater agreement beyond chance.

For the study selection stage, pilot screening was conducted on a randomly selected subset representing 10% of all search results. Two reviewers (MSV and LPV) independently examined titles and abstracts according to predefined eligibility criteria, working in a blinded, separate manner. Agreement was quantified using Cohen’s kappa, with a value ≥ 0.80 indicating excellent concordance. When the kappa score was below this threshold, the reviewers discussed disagreements and refined the inclusion/exclusion criteria as needed. This calibration was repeated until the acceptable level of agreement was reached, after which the complete screening of records was undertaken.

During data extraction, the same calibration protocol was applied. A random sample comprising 10% of the included studies was selected, and both reviewers independently extracted information using a standardized data collection form. Inter-rater reliability was again evaluated with Cohen’s kappa. Any differences in extracted data were resolved through discussion, and if consensus could not be reached, a third reviewer (JM) acted as an arbitrator. Only once the kappa value met or exceeded 0.80 was the full-scale data extraction performed.

### 2.4. Risk of Bias Evaluation

The risk of bias for the included studies was evaluated using the Risk of Bias 2.0 (RoB 2.0) instrument, in accordance with the recommendations outlined in the Cochrane Handbook for Systematic Reviews of Interventions [19]. The appraisal considered five key domains: (1) adequacy of the randomization process, (2) deviations from the assigned interventions, (3) completeness of outcome data, (4) accuracy and objectivity of outcome measurement, including the blinding of assessors, and (5) selective reporting of outcomes. Each domain was rated as having low risk, high risk, or raising some concerns. These individual judgments were then synthesized to provide an overall risk-of-bias classification for each study.

The assessments were conducted independently by two reviewers (MSV and LPV). Any differences in their evaluations were discussed until agreement was reached, with a third reviewer (JM) consulted when consensus was not initially possible. The final determinations were documented using the structured framework provided by the RoB 2.0 tool.

### 2.5. Data Extraction and Variables

Data extraction encompassed general study information and clinical setting variables, including the number of participants, patient age range, duration of follow-up, all reported evaluation time points, and the number of dropouts. For each intervention arm, detailed information on restorative materials and adhesive procedures was collected, together with methodological characteristics such as trial registration, randomization procedures when reported, and the clinical criteria used to define restoration survival.

Prior to data extraction, eligibility screening was conducted to ensure methodological and clinical homogeneity. Only randomized clinical trials conducted in humans were retained. Eligible studies were required to evaluate restorations placed in NCCLs of permanent teeth, to include at least two intervention arms differing in etching strategy, and to report outcomes using validated clinical evaluation criteria. Studies involving active co-interventions likely to confound the effect of the restorative protocol were excluded before data extraction.

Etching strategies were classified according to the operative protocol applied and coded using a standardized scheme, including etch-and-rinse, self-etch, and selective-etch approaches, as well as clinically relevant protocol variants (e.g., dentin moisture control, enamel beveling, and the use of additional hydrophobic bonding layers). Adhesive systems were categorized and coded based on the number of application steps and their universality (one-step, two-step, or three-step; universal or non-universal). Restorative materials were likewise classified and coded according to their material class, encompassing composite resin subtypes, resin-modified glass-ionomer cements, glass-hybrid systems, compomers, ormocer-based materials, and other clinically relevant categories. Interventions that did not include an etching agent or adhesive system were coded accordingly as blank.

To ensure analytical consistency, each intervention arm was assigned a unique composite treatment code integrating the etching strategy, adhesive system, and restorative material category (Table 1). Each reported treatment was operationally defined as the combination of the three coding dimensions, concatenated using the symbol “|”, following the structure *Etching strategy | Adhesive system | Restorative material*. This composite identifier constituted the treatment label used throughout data extraction, network construction, and statistical analyses, ensuring consistency across studies and follow-up periods. Commercial product names were not used to define treatment nodes.

For the network meta-analysis, treatment nodes were defined *a priori* based on the applied restorative strategy rather than on individual commercial brands. Nodes were constructed by combining the coded etching approach, adhesive system when applicable, and restorative material category. Restorations performed with resin-modified glass-ionomer cement were predefined as a separate treatment node, reflecting their distinct bonding mechanism and clinical indication in NCCLs. This node definition strategy was applied uniformly to ensure reproducibility of the network structure.

RMGI was selected as the reference comparator in the network meta-analysis for both methodological and clinical reasons. Historically, RMGI restorations have been widely used in the management of non-carious cervical lesions because of their chemical adhesion to dentin, fluoride release, and relative tolerance to moisture contamination. In addition, RMGI represented a clinically distinct restorative approach compared with adhesive composite protocols and was consistently reported across multiple trials within the evidence base. Using RMGI as the reference treatment therefore provided a clinically interpretable anchor for comparing the relative performance of contemporary adhesive strategies.

Outcomes reported as truly continuous variables without an associated clinical cut-off or frequency distribution were not converted into event data and were considered non-extractable for the event-based analytical model.

For the outcomes, clinically unacceptable scores corresponding to restorations requiring repair or replacement were classified as failures, whereas all clinically acceptable scores were classified as non-failures. The predefined mapping rules used to convert ordinal clinical scores into binary outcomes are provided in Table 2 to ensure full analytical reproducibility.

For each study, the longest available follow-up was used for the primary NMA; earlier time points were used only for descriptive summaries/sensitivity analyses.

Sensitivity analyses were conducted to assess the robustness of the primary analytical strategy. First, alternative analyses were performed using earlier follow-up intervals when sufficient data were available, allowing evaluation of whether the use of the longest available follow-up influenced the direction or magnitude of treatment effects. Second, sensitivity analyses excluding comparisons with sparse event data were explored to examine the potential impact of continuity correction procedures. These analyses did not materially change the overall direction of the estimates and therefore the primary results based on the longest follow-up were retained.

### 2.6. Data Analysis

All statistical analyses were performed using RStudio 2025.05.0 Build 496.

Network meta-analyses were conducted separately for each clinical outcome of interest using the *netmeta* package in RStudio (Version 2026.01.0, Build 392; RStudio Team, Boston, MA, USA) [20]. The analytical unit of comparison was the restorative strategy, defined a priori as a unique combination of three clinically relevant dimensions:

(i) etching approach,
(ii) adhesive system category, and
(iii) restorative material.

This node definition ensured that each intervention represented a clearly specified, clinically interpretable restorative protocol rather than isolated adhesive or etching components.

All eligible treatment arms contributing data to a given outcome were incorporated into a single outcome-specific network and analyzed under a random-effects model. Relative treatment effects were estimated as risk ratios (RRs), with corresponding 95% confidence intervals (CIs). Between-study heterogeneity was accounted for using the DerSimonian–Laird estimator.

Sparse event data were addressed using a predefined continuity correction approach. When at least one study arm in a comparison contained zero events, a continuity correction of 0.5 was applied to the corresponding 2 × 2 cells to allow estimation of risk ratios. Studies reporting zero events in all arms were retained for descriptive characterization of the evidence base but did not contribute information to the relative effect estimation on the risk ratio scale.

Multi-arm trials were incorporated within the network using the standard netmeta framework, which accounts for the correlation induced by shared comparator groups and prevents double counting of participants. This approach ensured that multi-arm evidence contributed appropriately to both direct and indirect estimates without inflating precision.

Forest plots were generated for each outcome to visualize the comparative risk of restoration failure across all restorative strategies included in the connected network. This outcome-based network approach enabled simultaneous comparison of multiple restorative strategies within a coherent analytical framework while preserving the core assumptions of transitivity and consistency required for valid network meta-analysis.

The assumption of transitivity was evaluated qualitatively by assessing the distribution of potential effect modifiers across treatment comparisons, including clinical evaluation criteria, follow-up duration, operator setting, lesion characteristics, and isolation procedures. No clinically meaningful imbalances were identified that would preclude combining direct and indirect evidence within the networks.

### 2.7. Certainty and Confidence of Evidence

The certainty of evidence for each outcome and comparison was assessed using the CINeMA (Confidence in Network Meta-Analysis) [21] framework, which evaluates six domains: within-study bias, across-study bias, indirectness, imprecision, heterogeneity, and incoherence.

Analyses were performed on the CINeMA web platform, generating graphical confidence profiles and summary matrices.

Indirectness was judged to be minimal across comparisons, as the included studies closely matched the prespecified population, interventions, and outcomes; nevertheless, this judgment was interpreted within the broader CINeMA framework, considering the clinical and methodological diversity inherent to NCCL trials.

## 3. Results

### 3.1. Study Selection and PRISMA Flowchart

A total of 831 records were identified through database searching, including PubMed (*n* = 230), Web of Science (*n* = 152), Scopus (*n* = 221), Embase (*n* = 192), and the Cochrane Library (*n* = 36). After removal of duplicate records (n = 482), 349 studies remained and were screened based on titles and abstracts. Of these, 256 records were excluded for failing to meet the predefined eligibility criteria.

Ninety-three reports were subsequently sought for full-text retrieval; however, five reports could not be retrieved. Consequently, 88 full-text articles were assessed for eligibility. At this stage, 15 reports were excluded due to insufficient outcome reporting (*n* = 2), wrong comparison (*n* = 5), wrong intervention (*n* = 6), wrong population (*n* = 1), or use of an inappropriate restorative material (*n* = 1).

**Figure 1:**
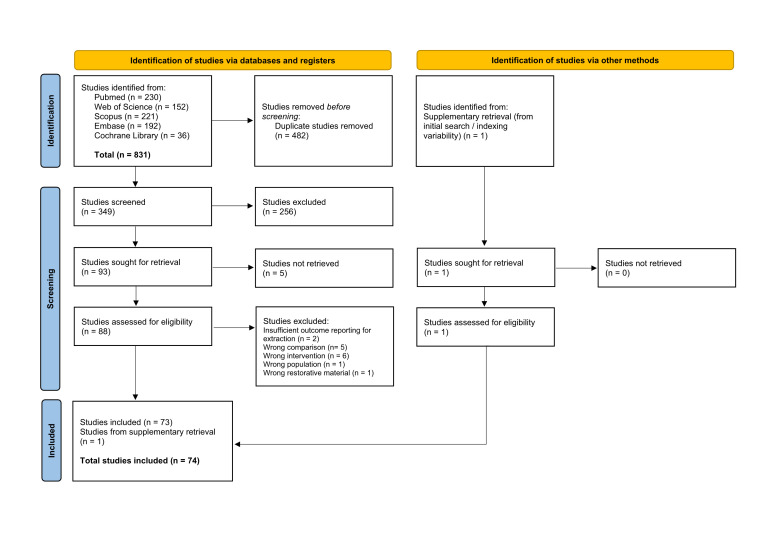
PRISMA 2020 flow diagram showing the study selection process.

In parallel, one additional study was identified through supplementary retrieval methods addressing indexing variability. This report was successfully retrieved and assessed for eligibility, and it met the inclusion criteria. Ultimately, 73 studies from database searches and one study from supplementary retrieval were included, resulting in a total of 74 studies incorporated into the final qualitative and quantitative synthesis (Figure 1).

All details regarding the studies excluded and included during the final screening stage were provided in Supplementary File S2.

### 3.2. Overview and Characteristics of Studies

The studies were conducted across multiple geographic regions, with the highest contribution originating from South America and Europe, particularly Brazil (n = 27) and Turkey (n = 11), followed by Belgium (n = 7), the United States (n = 5), and Germany (n = 5). The remaining studies were distributed across Asia, the Middle East, and other European countries, reflecting a broad international representation of clinical research on non-carious cervical lesion restorations.

Most investigations employed parallel-group randomized clinical trial designs, with the number of intervention arms ranging from two to four per study. Collectively, the included trials evaluated a wide spectrum of adhesive strategies and restorative material combinations, encompassing etch-and-rinse, self-etch, and selective-etch approaches, applied with both conventional and contemporary resin-based restorative materials.

Regarding follow-up duration, all studies reported baseline assessments, while longitudinal evaluations varied considerably. Short- to mid-term follow-ups (≤12 months) were common, whereas a substantial proportion of studies extended observations to 24 and 36 months, and a smaller subset reported long-term outcomes of 60 months or longer, with maximum follow-up periods reaching up to 72 months. This heterogeneity in follow-up intervals allowed the assessment of both early and longer-term clinical performance.

Eligibility criteria were largely consistent across studies, predominantly including adult patients with non-carious cervical lesions in permanent teeth, generally under conditions of good general and oral health. Despite minor variations, these criteria ensured clinical comparability across the evidence base.

Given the volume and heterogeneity of methodological characteristics, intervention protocols, and follow-up schedules, the complete descriptive overview of individual studies, including detailed inclusion criteria, intervention specifications, follow-up schemes, and authors’ conclusions, is provided in Supplementary File S3.

### 3.3. Risk of Bias Assessment

**Figure 2:**
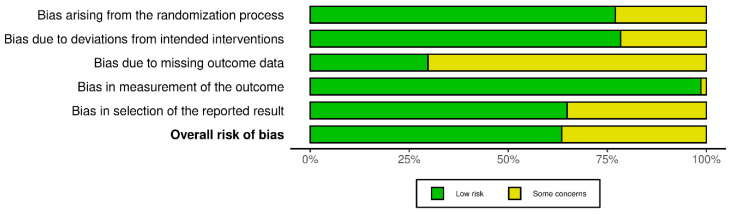
Summary of risk of bias judgments across RoB 2 domains.

The risk of bias assessment using the RoB 2 tool revealed a predominance of low risk of bias across most domains, as illustrated in Figure 2. Bias in measurement of the outcome (D4) was judged as low risk in the majority of studies, with only a limited proportion raising some concerns, followed by bias arising from the randomization process (D1) and bias due to deviations from intended interventions (D2).

In contrast, bias due to missing outcome data (D3) represented the most frequent source of methodological concern, accounting for the highest proportion of judgments classified as some concerns. Bias in selection of the reported result (D5) was predominantly judged as low risk, although some concerns were observed in a subset of studies due to limited information on outcome pre-specification.

Overall risk of bias judgments mirrored this domain-level distribution, with most studies classified as having low overall risk of bias, and the remaining studies downgraded to some concerns, primarily driven by incomplete reporting or attrition-related issues. A detailed, study-level risk of bias assessment is provided in Supplementary File S4.

### 3.4. Network Meta-Analysis

Among the eight predefined clinical outcomes, quantitative network meta-analysis was performed for marginal adaptation and retention loss. For the remaining outcomes (anatomic form, fracture, marginal discoloration, postoperative sensitivity, secondary caries, and surface texture), effect estimates were not pooled in the primary analysis and are reported descriptively in the Supplementary File S5.

In addition to marginal adaptation and retention loss, several other predefined outcomes were assessed, including anatomic form, fracture, marginal discoloration, postoperative sensitivity, secondary caries, and surface texture. For several of these predefined outcomes, effect estimates did not show statistically significant differences between interventions and were characterized by sparse or heterogeneous comparisons. These outcomes are reported in detail in the Supplementary Material.

In contrast, the remaining outcomes were affected by sparse data, fragmented or disconnected networks, and/or limited overlap between interventions. As a result, these outcomes did not meet the minimum structural requirements for robust network modeling and were therefore not prioritized for the primary quantitative synthesis.

To support the transitivity assumption, the main prespecified effect modifiers (clinical evaluation criteria, follow-up schedules, clinical setting, lesion characteristics, and isolation procedures) were inspected across the principal contrasts and did not show clinically meaningful imbalances.

#### 3.4.1. Network Geometry of the Included Studies

The network for the marginal adaptation outcome comprised 57 clinical studies, encompassing 28 distinct interventions and 6798 patients. Across this evidence base, the network geometry enabled 378 theoretically possible pairwise comparisons, of which 62 were informed by direct clinical evidence. Importantly, the network was fully connected, enabling comparative inference across all included interventions within a single analytical framework.

From a design perspective, the evidence base included 33 two-arm trials and 24 multi-arm studies. A total of 1772 events related to marginal adaptation were recorded across all study arms. Most studies (45 trials) reported no zero-event arms, whereas 12 studies included at least one arm with zero events, and 4 studies presented zero events across all arms. This distribution reflects the presence of both informative comparisons and sparse data across the network.

For the retention loss outcome, the evidence network was expanded in both size and complexity, incorporating 61 clinical studies and 33 distinct interventions, with an overall sample of 7338 patients. The resulting network structure supported 528 theoretically possible pairwise comparisons, of which 75 were informed by direct evidence, while maintaining a fully connected geometry, thereby allowing comprehensive indirect and mixed comparisons across all interventions.

The study designs contributing to this network comprised 34 two-arm trials and 27 multi-arm studies, reflecting a heterogeneous yet methodologically complementary evidence base. A total of 584 retention loss events were observed across the network. In contrast to the marginal adaptation outcome, a substantial proportion of studies (32 trials) included at least one zero-event arm, and 6 studies reported zero events across all arms, whereas only 29 studies had no zero events. A higher proportion of zero-event arms was observed for the retention loss outcome compared with marginal adaptation.

#### 3.4.2. Network Meta-Analysis Results According to Outcomes

**Figure 3:**
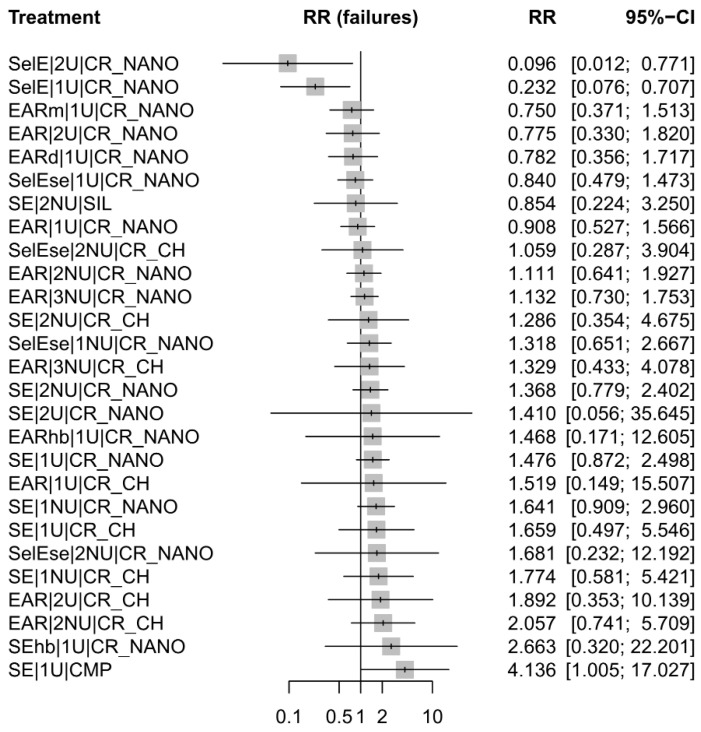
Forest plot of relative risks for marginal adaptation failure comparing restorative and adhesive strategies against resin-modified glass ionomer (RMGI) as the reference treatment.

The forest plot from Figure 3 summarizes the risk ratio (RR) of failure for marginal adaptation across the evaluated restorative and adhesive strategies, using RMGI as the reference comparator. In the network meta-analysis for marginal adaptation, two adhesive–restorative strategies, SelE|2U|CR_NANO and SelE|1U|CR_NANO, demonstrated a statistically significant reduction in the risk of marginal adaptation failure compared with the resin-modified glass ionomer (RMGI), as their relative risks (RRs) and corresponding 95% confidence intervals (CIs) were entirely below unity. In contrast, SE|1U|CMP showed a significantly poorer performance than RMGI, with an RR greater than 1.0 and a 95% CI that did not cross the null value, indicating a higher risk of marginal adaptation failure for this outcome.

All remaining treatment comparisons yielded RRs with 95% CIs overlapping 1.0, indicating no statistically significant differences relative to RMGI. Accordingly, no statistically significant differences were observed between these interventions and the reference treatment.

**Figure 4:**
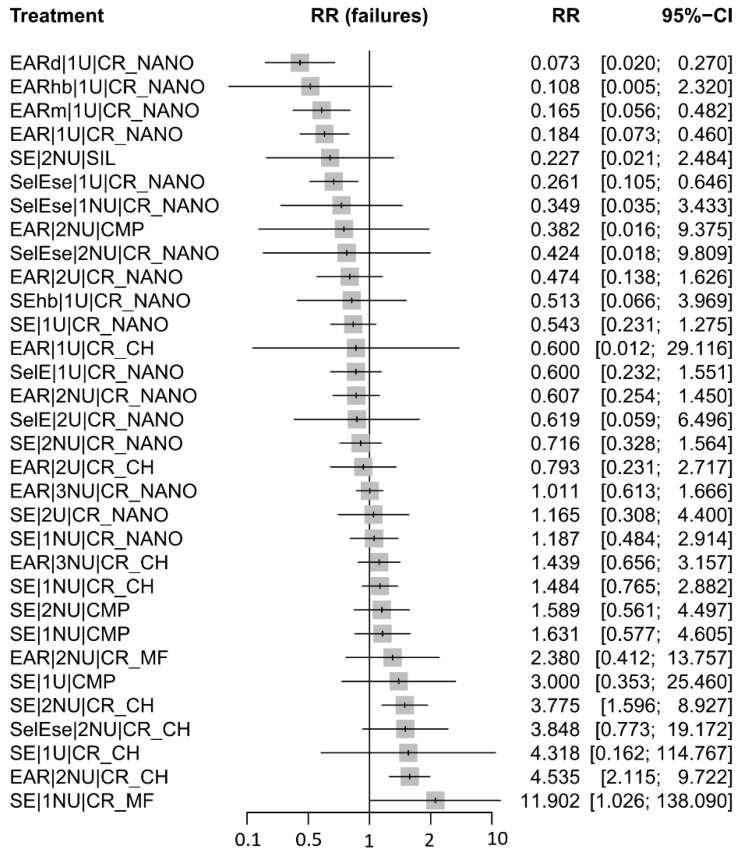
Forest plot of relative risks for retention loss comparing restorative and adhesive strategies against resin-modified glass ionomer (RMGI) as the reference treatment.

The forest plot for the outcome of retention loss (Figure 4) illustrates differential performance among the evaluated restorative and adhesive strategies when compared with the resin-modified glass ionomer (RMGI) as the reference treatment. Specifically, EARd|1U|CR_NANO, EARm|1U|CR_NANO, EAR|1U|CR_NANO, and SelEse|1U|CR_NANO demonstrated relative risks (RRs) with corresponding 95% confidence intervals (CIs) entirely below 1.0, indicating a statistically significant lower risk of retention loss compared with RMGI.

In contrast, SE|2NU|CR_CH, EAR|2NU|CR_CH, and SE|1NU|CR_MF exhibited RRs greater than 1.0 with 95% CIs entirely above the null value, reflecting a significantly higher risk of retention loss relative to the RMGI comparator.

All remaining treatment comparisons yielded RRs with 95% CIs overlapping unity, indicating no statistically significant differences with respect to the RMGI. Accordingly, these interventions can be interpreted as having retention outcomes broadly comparable to those of the reference treatment under the evaluated conditions.

### 3.5. Overall Treatment Ranking

Because only marginal adaptation and retention loss generated connected networks suitable for quantitative synthesis, the ranking framework was derived exclusively from the estimates obtained for these two outcomes. The ranking visualization should therefore be interpreted as a descriptive summary of the relative positioning of treatments within these networks rather than as a global ranking across all predefined clinical outcomes.

**Figure 5:**
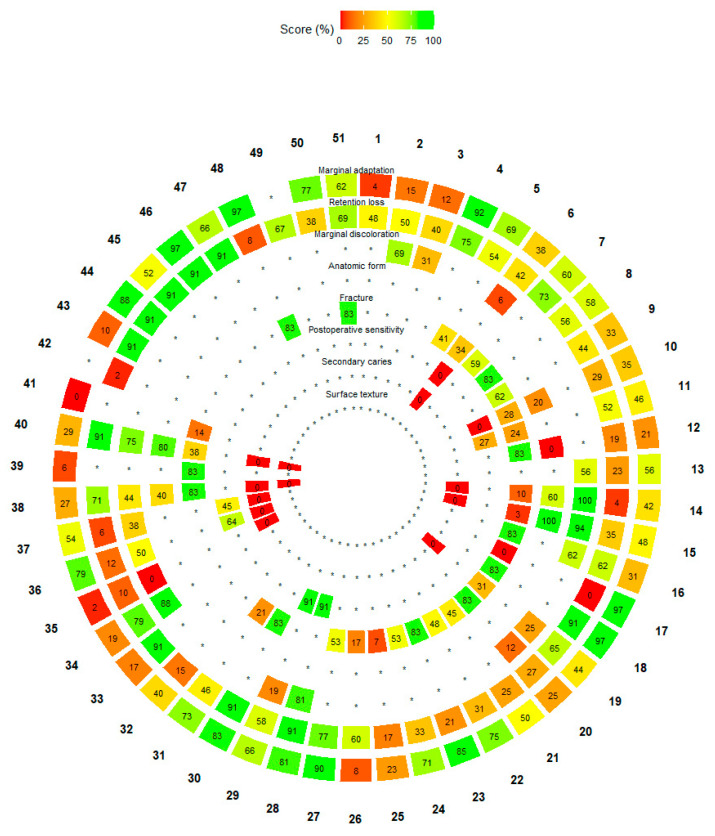
P-score–based circular ranking of 51 treatment combinations across clinical outcomes in the network meta-analysis. **Treatments**: 1 = EAR|3NU|CR_CH; 2 = SE|2NU|CR_CH; 3 = SE|1NU|CR_CH; 4 = SelE|2U|CR_NANO; 5 = EAR|2U|CR_NANO; 6 = SE|1U|CR_NANO; 7 = EARd|1U|CR_NANO; 8 = EARm|1U|CR_NANO; 9 = SelEse|1U|CR_NANO; 10 = EAR|2NU|CR_NANO; 11 = SE|2NU|CR_NANO; 12 = SE|1NU|CR_NANO; 13 = EAR|2U|CR_CH; 14 = EAR|2NU|CR_CH; 15 = RMGI; 16 = EAR|2NU|CR_MF; 17 = CR_saFLW; 18 = SelE|3NU|CR_NANO; 19 = EAR|1U|CR_NANO; 20 = SE|1NU|CR_MF; 21 = SE|1U|ORM; 22 = SelEse|1U|ORM; 23 = EARd|1U|ORM; 24 = EAR|1U|ORM; 25 = EAR|3NU|CR_NANO; 26 = SE|1U|CR_CH; 27 = EAR|1U|CR_CH; 28 = SelEse|1NU|LYR_SYS; 29 = SE|1NU|LYR_SYS; 30 = EARhb|1U|CR_NANO; 31 = SEhb|1U|CR_NANO; 32 = SE|1NU|CMP; 33 = EAR|2NU|CMP; 34 = SelEse|2NU|CR_CH; 35 = SE|1U|CMP; 36 = EAR|1NU|CR_MF; 37 = SE|2NU|CR_MF; 38 = SE|1U|CR_FLW; 39 = SelE|1U|CR_FLW; 40 = EAR|1U|CR_FLW; 41 = EAR|3NU|CR_FLW; 42 = SE|2NU|CMP; 43 = SelEse|1NU|CR_NANO; 44 = SelEse|2NU|CR_NANO; 45 = SEeb|2NU|CR_MF; 46 = SelEse|2NU|CR_MF; 47 = SelEseB|2NU|CR_MF; 48 = SE|2U|CR_NANO; 49 = SelEse|1U|CR_FLW; 50 = SelE|1U|CR_NANO; 51 = SE|2NU|SIL. (*) denotes that the corresponding outcome was not assessed in that treatment group.

A total of 51 distinct treatment combinations were identified and ranked across the outcomes included in connected NMAs using P-scores derived from the frequentist network meta-analysis (Figure 5). Overall, treatment 28 (SelEse|1NU|LYR_SYS) consistently ranked favorably across most outcomes in the P-score framework. Nevertheless, these findings should be interpreted cautiously, as P-scores reflect relative ranking probabilities rather than definitive evidence of superiority.

For the marginal-adaptation outcome, treatments 4, 17, 18, 27, 46, and 48 achieved P-scores above 90%, indicating a high probability of being among the best-performing interventions for this outcome. In contrast, for retention loss, the most favorably ranked treatments were 18, 28, 30, 33, 40, and 43–47, all of which also exhibited P-scores exceeding 90%, suggesting a lower relative risk of failure within the ranking framework.

Despite these favorable rankings, P-scores should not be overinterpreted. They do not account for the magnitude of effect sizes, the overlap of confidence intervals, or the statistical significance of individual comparisons. Accordingly, the ranking results should be considered complementary and exploratory and always interpreted in conjunction with the corresponding forest plots and network estimates, which provide the definitive basis for comparative inference.

### 3.6. Confidence Assessment in the Network Using CINeMA

**Figure 6:**
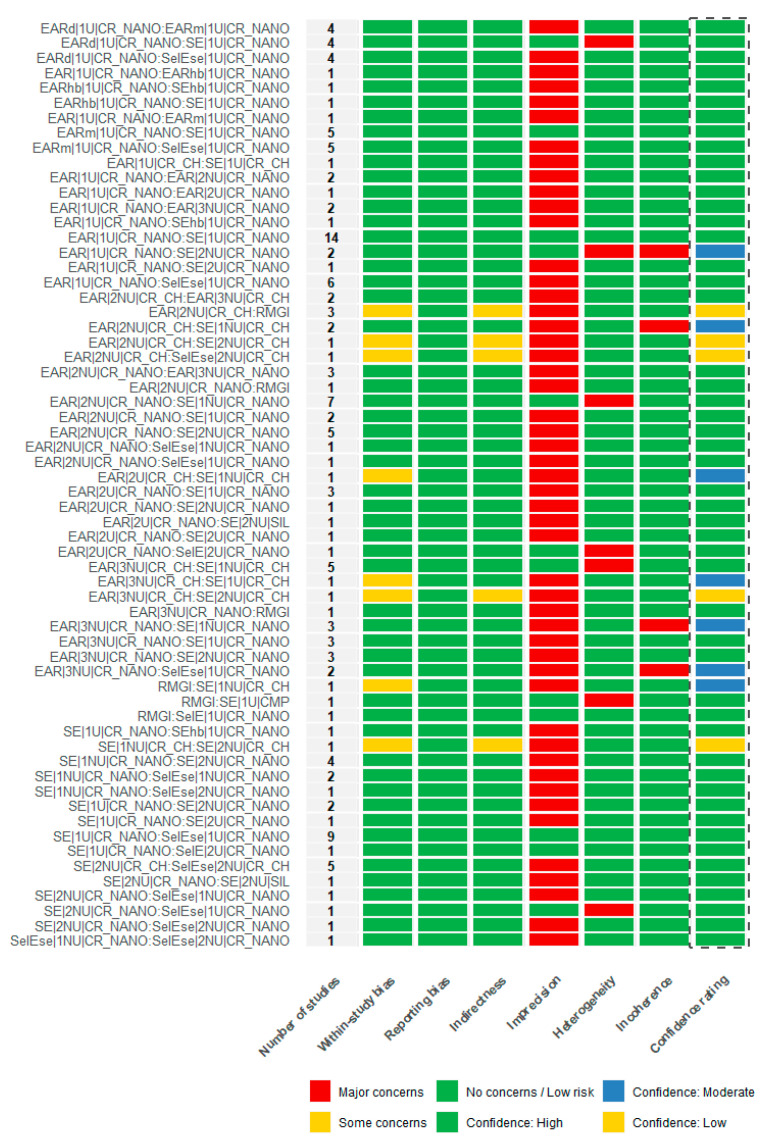
CINeMA confidence assessment for treatment comparisons evaluating marginal adaptation, based on the random-effects network meta-analysis. The figure summarizes judgments across the CINeMA domains (within-study bias, reporting bias, indirectness, imprecision, heterogeneity, and incoherence) and the resulting overall confidence ratings (dashed-line box) for each comparison, supporting the internal consistency and robustness of the network estimates for the marginal adaptation outcome.

**Figure 7:**
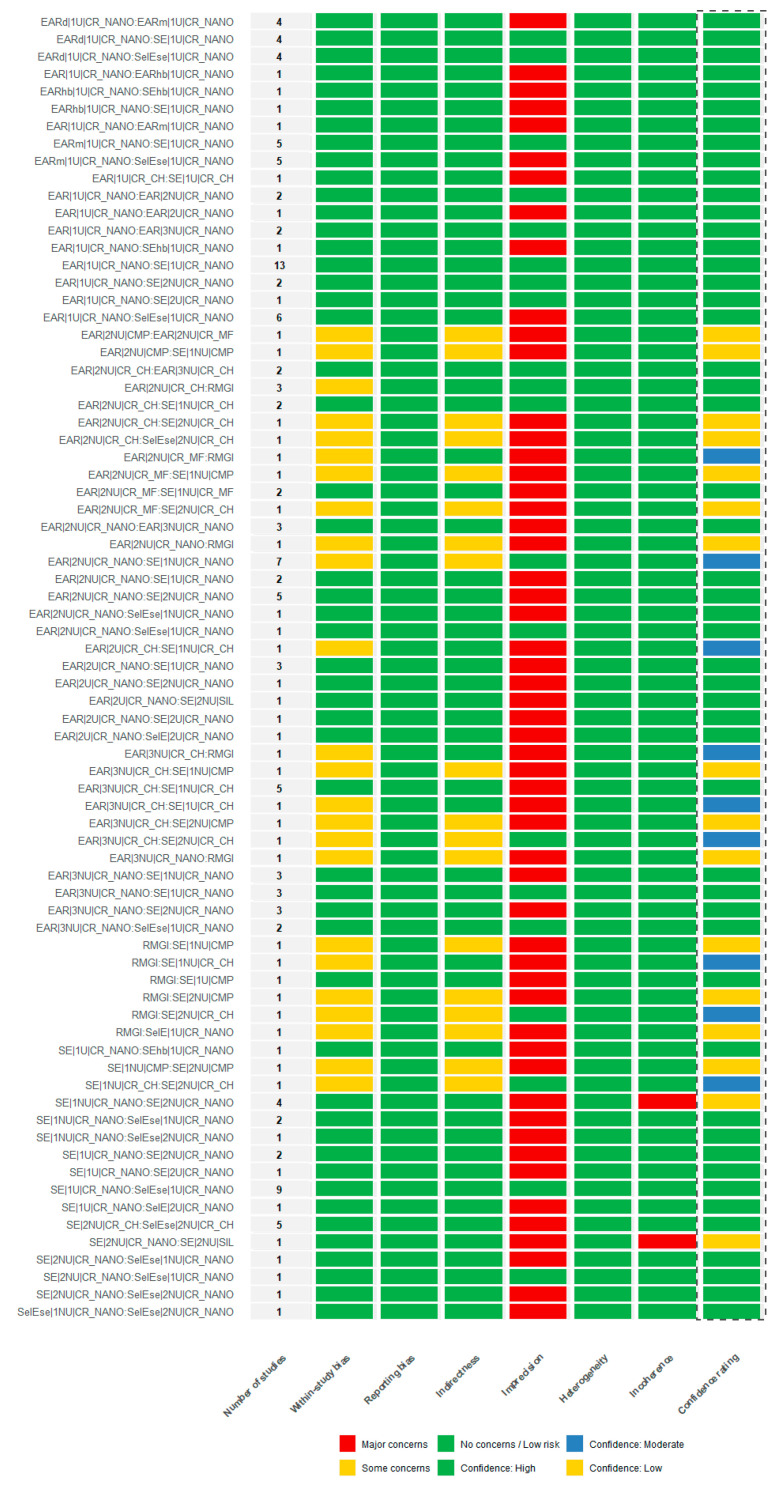
CINeMA confidence assessment for treatment comparisons evaluating retention loss, based on the random-effects network meta-analysis. The figure summarizes judgments across the CINeMA domains (within-study bias, reporting bias, indirectness, imprecision, heterogeneity, and incoherence) and the resulting overall confidence ratings (dashed-line box) for each comparison, supporting the network’s internal consistency and robustness for the retention loss outcome.

The certainty of evidence for the network meta-analyses was assessed using the CINeMA framework applied to the random-effects risk ratio models for the marginal adaptation and retention loss outcomes. Overall confidence judgments across CINeMA domains are summarized in Figure 6 and Figure 7, respectively. In addition, league tables reporting the estimated relative treatment effects alongside their corresponding CINeMA confidence ratings are provided in Supplementary File S6, allowing interpretation of effect magnitude in conjunction with the certainty of the evidence for each pairwise comparison.

Across both networks, the majority of treatment comparisons were judged as having *no concerns* in the domains of within-study bias and reporting bias, indicating that the methodological quality of the contributing trials was generally adequate and that selective outcome reporting was unlikely to have influenced the estimated relative effects.

No relevant concerns were identified regarding indirectness for either outcome, reflecting a close alignment between the populations, interventions, comparators, and outcomes assessed in the primary studies and those defined in the respective network questions. This supported the clinical applicability of the relative treatment effect estimates for both marginal adaptation and retention loss.

The main limitations across the two outcomes were related to imprecision, with several comparisons rated as having *major concerns*. These judgments were primarily driven by wide confidence intervals and a limited number of studies informing specific comparisons, particularly those supported by sparse evidence or small sample sizes. In addition, *major concerns* related to heterogeneity were identified for a subset of comparisons in both networks, indicating variability in clinical or methodological characteristics across studies that could affect the stability of the relative effect estimates.

No relevant concerns were identified in the incoherence domain for either outcome. Clinical interpretation of treatment effects was primarily based on the magnitude and precision of the estimated risk ratios rather than on deviation from the null value alone. Relative risks were interpreted in conjunction with their 95% confidence intervals and the clinical plausibility of the observed differences, recognizing that statistically non-significant estimates may still reflect clinically relevant trends when supported by consistent patterns across studies.

Under this framework, agreement between direct and indirect evidence was supported by the global incoherence tests based on random-effects design-by-treatment interaction models, which showed no evidence of inconsistency for marginal adaptation (χ^2^ = 31.478, 40 degrees of freedom; *p* = 0.830) or for retention loss (χ^2^ = 42.481, 43 degrees of freedom; *p* = 0.494). These findings indicated that any variability between direct and indirect estimates was small in relation to a clinically important effect size and did not threaten the internal consistency of either network.

Overall, the integration of all CINeMA domains resulted in a high confidence rating for the majority of treatment comparisons across both outcomes (Figure 6 and Figure 7).

Across both outcomes, the integration of CINeMA domains resulted in predominantly high confidence ratings for most treatment comparisons, while several comparisons were downgraded due to imprecision or heterogeneity.

## 4. Discussion

### 4.1. Summary of Main Findings

Two outcome-specific networks were constructed to address the primary research question, corresponding to marginal adaptation and retention loss. These networks integrated a large and methodologically comparable body of evidence, comprising randomized clinical trials with similar eligibility criteria, outcome definitions based on validated clinical indices (USPHS/FDI), and broadly overlapping follow-up ranges. Although variability existed across studies in terms of follow-up duration, operator setting, and restorative protocols, the overall distribution of clinical and methodological characteristics was balanced across treatment comparisons, resulting in limited between-study heterogeneity within the connected networks. This evidence structure supported stable comparative estimates while allowing the identification of differential performance among restorative strategies.

For marginal adaptation, the most competitive performance was observed for selective-etch strategies (SelE) combined with 1- or 2-step universal adhesive systems (1U or 2U) and nanocomposite resins (CR_NANO), which demonstrated a statistically significant reduction in the risk of marginal adaptation failure compared with resin-modified glass-ionomer cement (RMGI). In contrast, a self-etch strategy (SE) combined with a compomer material (CMP) was associated with a significantly higher risk of marginal adaptation failure, indicating inferior performance for this outcome. All other restorative strategies showed no statistically significant differences relative to the reference comparator.

For retention loss, superior clinical performance was primarily associated with etch-and-rinse strategies (EAR, including EARd and EARm) and selective-etch strategies (SelE or SelEse) applied with universal adhesive systems (1U or 2U) and nanocomposite resins (CR_NANO), which were linked to a statistically significant lower risk of restoration loss when compared with RMGI. Conversely, self-etch (SE) and etch-and-rinse (EAR) strategies combined with non-universal adhesive systems (1NU or 2NU) and alternative restorative materials, including CMP and microfilled composite resins (CR_MF), demonstrated a significantly higher risk of retention loss relative to the reference treatment.

Although several strategies demonstrated relative risk reductions compared with the reference treatment, the absolute clinical magnitude of these differences should be interpreted cautiously. In many cases, the baseline failure rates reported in NCCL trials are relatively low, meaning that even moderate relative risk reductions may translate into modest absolute differences in clinical practice. Consequently, the practical significance of these findings should be interpreted in conjunction with operator factors, substrate conditions, and patient-specific characteristics rather than relying exclusively on relative effect estimates.

The interpretation of these findings is primarily supported by the network forest plots, which provide the most direct and quantitative evidence through relative effect estimates and confidence intervals. The P-score–based ranking wheel offers a complementary, descriptive summary of the relative positioning of treatments within the network; however, P-scores are derived from point estimates and their variance structure and do not account for the magnitude of effect sizes, overlap of confidence intervals, or statistical significance of individual comparisons [22]. Importantly, treatment rankings derived from P-scores should not be interpreted as evidence of superiority unless supported by statistically significant and precise effect estimates. Rankings primarily reflect the relative ordering of point estimates within the network and do not account for the uncertainty surrounding those estimates. Accordingly, ranking results should be viewed as an auxiliary tool to facilitate pattern recognition across multiple interventions, rather than as a precise or definitive source of comparative evidence.

The exclusion of the remaining outcomes from quantitative synthesis was primarily driven by sparse data, fragmented network structures, and limited overlap between interventions, which constrained the feasibility of reliable network modeling.

### 4.2. Comparison with Previous Evidence

The present findings were compared with conceptually similar syntheses [23,24,25,26,27] that identify convergences and plausible sources of divergence regarding adhesive strategy, comparator choice, and outcome definition.

Overall, a consistent pattern across the external evidence was that differences in clinical performance tended to be outcome-dependent and frequently modest, with clearer signals emerging for retention under specific application modes and for marginal parameters under protocols incorporating more extensive enamel management. In this context, the present network meta-analysis, which classified interventions as composite strategies (etching approach × adhesive system × restorative material) and used the resin-modified glass ionomer (RMGI) as the reference comparator, expanded previous category-level comparisons by enabling discrimination among clinically relevant protocol combinations.

Regarding retention-related performance, the present analysis showed statistically significant reductions in retention loss versus RMGI for several etch-and-rinse–based strategies combined with nano-filled composites (EARd|1U|CR_NANO; EARm|1U|CR_NANO; EAR|1U|CR_NANO) and for a selective-etch/self-etch–related protocol (SelEse|1U|CR_NANO). This directionality was coherent with syntheses focused on universal adhesives in studies from Assis et al. [23] and Josic et al. [26], in which the application mode was reported to influence retention more consistently than marginal outcomes. In Josic et al. [26], self-etch application of universal adhesives was associated with a higher risk of retention loss than etch-and-rinse at 12 and 18/24 months, whereas marginal outcomes did not show statistically significant differences. In Assis et al. [23], etch-and-rinse application was similarly associated with improved retention, while self-etch application was discussed as potentially advantageous for postoperative sensitivity. Although the comparator structure differed (mode-to-mode contrasts in Assis et al. [23] and Josic et al. [26] versus RMGI-anchored contrasts in the present network), both lines of evidence were compatible with the interpretation that protocols incorporating more intensive conditioning could yield more stable retention under certain clinical contexts.

For marginal adaptation, the present network identified selective-etch strategies combined with nano-filled composite restorations (SelE|2U|CR_NANO and SelE|1U|CR_NANO) as significantly reducing the risk of marginal adaptation failure compared with RMGI, whereas SE|1U|CMP was associated with significantly poorer performance than the RMGI. This outcome-specific profile was broadly consistent with the emphasis in Dreweck et al. [25] that marginal parameters could favor more robust etching approaches, despite an absence of clear retention superiority for three-step etch-and-rinse systems over one-step self-etch adhesives. In other words, while Dreweck et al. [25] did not support a categorical retention advantage for three-step etch-and-rinse adhesives, it did indicate that marginal integrity outcomes could be more sensitive to the etching/conditioning approach, which aligned with the present observation that selective enamel etching protocols were among the best-performing strategies for marginal adaptation relative to the RMGI.

Apparent divergences across studies were plausibly attributable to differences in analytical granularity and comparator choice. The synthesis in de Assis et al. [24] evaluated one-step versus two-step self-etch adhesives and reported no statistically significant difference in retention while indicating a difference in marginal adaptation. In contrast, the present analysis compared multi-component strategies against an RMGI anchor and therefore addressed a different inferential target (relative performance versus a clinically relevant non-resin comparator rather than optimization within self-etch subclasses). Consequently, the present findings were not interpreted as contradicting de Assis et al. [24] study, but rather as extending the evidentiary framework by evaluating a broader intervention space and by distinguishing protocol-level combinations that could be masked when adhesive categories were pooled.

Finally, Liu et al. [27] was not NCCL-specific, as it synthesized evidence on occlusal cavities; however, it reported that an etch-and-rinse approach improved marginal adaptation/marginal discoloration at two years when compared with alternatives in that context. This external finding was treated as mechanistic-supportive rather than direct corroboration, but it remained compatible with the present observation that protocols incorporating targeted etching could influence marginal outcomes.

Beyond the comparisons already discussed, additional insights emerge when the present findings are contrasted with systematic reviews that examined the clinical performance of adhesive strategies in NCCLs from different analytical perspectives. For instance, the updated systematic review and meta-analysis evaluating universal adhesives in NCCLs reported that the adhesive strategy itself, self-etch versus total-etch, did not significantly influence clinical performance across outcomes such as retention, marginal adaptation, marginal discoloration, or postoperative sensitivity when assessed at follow-up periods up to 36 months [28]. These conclusions partially converge with the present results in the sense that most treatment comparisons did not demonstrate statistically significant differences relative to the RMGI reference, suggesting that many contemporary adhesive approaches may provide clinically acceptable performance under controlled conditions. However, the present network analysis extends those findings by demonstrating that specific protocol-level combinations, particularly those incorporating selective enamel etching combined with nano-filled composites, can still show measurable advantages for certain outcomes, particularly marginal adaptation.

From a mechanistic standpoint, the results reported in the universal-adhesive literature help contextualize these outcome-specific patterns. Universal adhesives incorporate functional monomers such as 10-MDP, which can chemically interact with hydroxyapatite and contribute to durable bonding through nano-layering and hydrophobic chain interactions [28]. Such chemical bonding mechanisms have been proposed to stabilize the adhesive interface and may partly explain why several adhesive strategies yield broadly comparable clinical outcomes in NCCLs despite differences in etching protocols. This biochemical bonding capability may attenuate the clinical differences that would otherwise be expected between etch-and-rinse and self-etch approaches, particularly in dentin-dominant substrates.

In parallel, evidence from meta-analyses comparing restorative materials in NCCLs has highlighted the importance of retention as a primary determinant of restoration longevity. For example, a systematic review comparing glass ionomer cements and composite resins reported that retention was the only clinical parameter showing a statistically significant difference between materials, underscoring its central role in the evaluation of NCCL restorations [29]. This observation is conceptually consistent with the present findings, where retention loss constituted one of the two outcomes for which a sufficiently connected evidence network could be constructed and where distinct adhesive-restorative combinations demonstrated differential performance relative to the RMGI comparator. Taken together, these convergent observations reinforce the notion that retention-related endpoints often provide the most sensitive indicator of clinical differences among adhesive strategies.

Additional contextualization can be drawn from recent umbrella-level evidence on restorative materials and adhesive protocols. High-level syntheses have consistently emphasized that adhesive strategy and clinical context act as major prognostic modifiers of restoration survival, with multi-step etch-and-rinse systems frequently demonstrating superior marginal integrity and retention under challenging clinical conditions such as cervical or root lesions [30]. While the present analysis did not directly compare adhesive systems at the category level, the protocol-based classification employed in this network meta-analysis indirectly supports this interpretation, as several of the most favorably ranked treatment combinations incorporated etch-and-rinse or selective-etch components within their protocol structure.

Finally, several recent syntheses have highlighted the influence of enamel conditioning on restoration success. Evidence suggests that failure rates increase when enamel etching is omitted in self-etch systems, whereas selective phosphoric acid etching can significantly improve clinical outcomes in carious cervical restorations [31]. These observations align with the present finding that selective-etch protocols combined with nano-filled composites were among the best-performing strategies for marginal adaptation. Consequently, although simplified adhesive systems continue to gain popularity due to their ease of application, the cumulative evidence suggests that targeted enamel conditioning may still provide measurable clinical advantages in NCCL restorations.

### 4.3. Methodological Strengths and Innovations

A key methodological strength of this study lies in the use of a network meta-analytic framework specifically tailored to complex restorative strategies, allowing the simultaneous comparison of multiple interventions within a unified analytical structure. Another important innovation was the a priori definition of network nodes based on a standardized coding scheme that integrated etching approach, adhesive system category, and restorative material. This strategy-oriented classification reduced ambiguity in intervention definitions, improved reproducibility, and avoided post hoc aggregation of heterogeneous protocols, which represents a frequent limitation in previous evidence syntheses on adhesive systems.

The application of the CINeMA framework further contributed to methodological transparency by explicitly evaluating confidence across multiple domains, including risk of bias, indirectness, heterogeneity, imprecision, and incoherence. This structured appraisal facilitated a nuanced interpretation of network estimates and ensured that conclusions were supported not only by statistical significance but also by the overall certainty of the evidence.

Collectively, these methodological features distinguish the present review from earlier analyses focused on isolated adhesive categories or pairwise comparisons, providing a reproducible and extensible framework for comparative evaluation of restorative strategies in non-carious cervical lesions.

### 4.4. Limitations

Several limitations of the present study should be acknowledged when interpreting the findings. First, although the systematic review included a large number of clinical studies, only a subset of outcomes yielded sufficiently connected networks to support quantitative network meta-analysis. For outcomes other than marginal adaptation and retention loss, sparse data and fragmented network structures precluded reliable comparative synthesis, limiting the scope of quantitative inference.

Second, the unit of analysis in most included trials was the restoration rather than the patient, and information on intra-patient correlation or clustering of multiple restorations within the same individual was generally not reported. As a result, statistical adjustment for within-patient dependence was not feasible, which may have led to an overestimation of precision for some comparisons. This limitation was explicitly considered during interpretation and contributed to downgrading decisions within the imprecision domain of the CINeMA framework.

Third, despite overall clinical and methodological comparability across studies, heterogeneity remained present in aspects such as follow-up duration, operator setting, and restorative protocols. Although no major imbalances were identified that would invalidate the assumption of transitivity, this residual variability may have influenced effect estimates for specific strategy combinations, particularly those informed by a limited number of studies.

Fourth, the relative performance of certain restorative strategies, most notably the RMGI, was informed by comparatively fewer trials within the connected networks than composite-based strategies. Consequently, estimates involving these interventions should be interpreted with caution, as they may be more sensitive to small-study effects or sparse data.

Finally, treatment ranking based on P-scores should be interpreted as descriptive and complementary. Rankings do not account for the magnitude of effect sizes, overlap of confidence intervals, or statistical uncertainty, and therefore should not be considered definitive evidence of superiority when evaluated independently of the corresponding network effect estimates.

In addition, the absence of statistical adjustment for clustering may affect clinical outcomes differently. Retention loss, which reflects complete restoration failure, may be less sensitive to intra-patient dependence because such events are relatively rare and more strongly related to restoration-specific factors. In contrast, marginal adaptation may be more susceptible to patient-level characteristics such as occlusal stress distribution or oral hygiene habits, which could introduce additional correlation among restorations placed in the same individual. Future clinical trials could mitigate this limitation by adopting analytical approaches that account for clustering at the patient level or by designing studies in which the patient, rather than the restoration, constitutes the primary unit of analysis.

### 4.5. Clinical Implications

From a clinical standpoint, the present findings support a strategy-oriented approach to the restoration of non-carious cervical lesions, emphasizing the combined selection of etching protocol, adhesive system category, and restorative material rather than reliance on any single component in isolation. The observed differences across restorative strategies highlight the importance of matching the adhesive protocol to the clinical substrate and operative conditions.

In routine clinical practice, selective-etch (SelE or SelEse) and etch-and-rinse approaches (EAR, including EARd and EARm) applied with universal adhesive systems (1U or 2U) appear particularly suitable when predictable enamel conditioning and durable retention are desired. These strategies may be especially relevant in cases with clearly defined enamel margins, where controlled phosphoric acid application can be reliably achieved.

Conversely, restorative protocols based on self-etch approaches (SE) combined with non-universal adhesive systems (1NU or 2NU) and alternative restorative materials, such as CMP or CR_MF, may require greater clinical caution. Their use may be more sensitive to operator technique, substrate variability, or material-specific limitations, particularly when long-term marginal integrity and retention are key treatment objectives.

The inclusion of resin-modified glass-ionomer cement (RMGI) as a material-based comparator provides additional clinical context rather than a direct alternative to adhesive composite strategies. Within the analyzed networks, RMGI may remain a reasonable option in specific clinical scenarios where moisture control is challenging or where fluoride release and chemical adhesion are prioritized; however, its comparative performance should be interpreted cautiously due to the limited amount of within-network evidence informing these estimates.

Finally, clinicians should consider that the available evidence is largely derived from outcomes assessed at the restoration level, without adjustment for clustering of multiple restorations within individual patients. This methodological characteristic may influence the apparent precision of some comparisons and underscores the need for cautious extrapolation of relative effect estimates to individual patient care.

### 4.6. Future Directions and Research Priorities

Future research should primarily aim to strengthen network connectivity and precision for restorative strategies that were underrepresented in the connected networks. Randomized clinical trials using balanced multi-arm designs and directly comparing etch-and-rinse (EAR), selective-etch (SelE/SelEse), and self-etch (SE) strategies across different adhesive system categories would reduce reliance on indirect evidence and improve the stability of comparative estimates.

The present findings also indicate a need for further investigation of resin-modified glass-ionomer cement (RMGI) within contemporary comparative frameworks. Although included as a clinically relevant comparator, the RMGI contributed limited within-network evidence relative to composite-based strategies. Future trials directly comparing the RMGI with current composite-based protocols using universal adhesives would allow a more informative assessment of its relative performance.

## 5. Conclusions

The findings of this network meta-analysis suggest that restorative strategies incorporating selective enamel etching or etch-and-rinse approaches combined with universal adhesive systems and nano-filled composite resins tended to demonstrate favorable performance for the outcomes of marginal adaptation and retention loss when compared with the reference treatment. However, statistically significant differences were limited to a subset of comparisons, and many estimates showed overlapping confidence intervals. Consequently, the observed patterns should be interpreted cautiously and primarily as indicative of potential differences in clinical performance rather than definitive evidence of superiority among restorative strategies.

Clinically, these findings support protocol-level selection for direct NCCL restorations with the goal of improving function-oriented longevity and minimizing maintenance attributable to marginal deterioration or restoration loss. Nevertheless, inferences should remain proportional to the certainty of evidence, and treatment selection should be interpreted in conjunction with the CINeMA judgments, particularly where imprecision or sparse data limited confidence in specific comparisons.

These conclusions should therefore be interpreted in light of the remaining uncertainty related to heterogeneity and imprecision within the available clinical evidence.

## Figures and Tables

**Table 1:** Coding scheme for etching strategies, adhesive systems, and restorative materials.

Etching Approach	Code
Etch-and-rinse	EAR
Self-etch (without prior etching)	SE
Selective-etch (enamel only phosphoric acid)	SelE
Etch-and-rinse (dry dentin)	EARd
Etch-and-rinse + moist dentin	EARm
Selective-etch (enamel only phosphoric acid) + self-etch on dentin	SelEse
Etch-and-rinse + extra hydrophobic bonding resin	EARhb
Self-etch + extra hydrophobic bonding resin	SEhb
Self-etch + enamel bevel	SEeb
Selective-etch (enamel only phosphoric acid) + self-etch on dentin + bevel	SelEseB
**Adhesive system category**	
1-step NOT universal adhesive	1NU
2-step NOT universal adhesive	2NU
3-step or more, NOT universal adhesive	3NU
1-step universal adhesive	1U
2-step universal adhesive	2U
**Restorative material**	
Conventional/microhybrid/hybrid composite resins	CR_CH
Nanofilled/nanocomposite/nanohybrid composite resins	CR_NANO
Microfilled composite resins	CR_MF
Flowable composite resins	CR_FLW
Layered restorative systems (layering technique)	LYR-SYS
Ormocer-based restorative materials	ORM
Silorane-based composite resins	SIL
Self-adhesive restorative materials	SADH
Resin-modified glass-ionomer cements (RMGIC)	RMGI
Glass-hybrid restorative systems	GH
Compomers (polyacid-modified resin composites)	CMP
Self-adhesive flowable composite	CR_saFLW

**Table 2:** Operationalization of clinical scores into failure events.

Clinical Evaluation System	Acceptable Outcome	Failure
USPHS (Ryge criteria)	Alpha/Bravo	Charlie
FDI criteria	Scores 1–3	Scores 4–5

## Data Availability

The original data presented in the study are openly available in Zenodo at https://doi.org/10.5281/zenodo.19131928.

## Supplementary Materials

The following supporting information can be downloaded at: https://www.mdpi.com/article/10.3390/jfb17040160/s1, **Supplementary File S1**. Search strategies and database-specific query formulations; **Supplementary File S2**. Full Reference List of Included Studies and Studies Excluded after Full-Text Assessment with Reasons; **Supplementary File S3**. Detailed Characteristics and Clinical Outcomes of the Included Randomized Controlled Trials; **Supplementary File S4**. Summary of risk of bias assessment across included studies using the Cochrane RoB 2 tool; **Supplementary File S5**. Descriptive Reporting of Predefined Outcomes Without Statistically Significant Differences; **Supplementary File S6**. League tables of relative treatment effects for marginal adaptation and retention loss (network meta-analysis).
