# Supplementary material for: Comparison of Adhesive Strategies with Different Etching Approaches on the Clinical Performance of Restorations in Non-Carious Cervical Lesions: A Systematic Review and Network Meta-Analysis
Source: J Funct Biomater. 2026 Mar 25;17(4):160. doi: 10.3390/jfb17040160 (PMC13117247; doi:10.3390/jfb17040160)
Supplement: Supplementary file 1 [file jfb-17-00160-s001.zip › jfb-4192088-Supplementary File S1.pdf]

Supplementary file S1. Search strategies and database-specific query formulations.

Database	Formulation/query
Pubmed	(<br>("noncarious cervical lesion"[tiab] OR "non-carious cervical lesion"[tiab] OR NCCL[tiab] OR "cervical lesion"[tiab] OR "cervical lesions"[tiab] OR "cervical abrasion"[tiab] OR "cervical erosion"[tiab] OR "cervical abfraction"[tiab]<br>OR "Tooth Abrasion"[MeSH] OR "Tooth Erosion"[MeSH])<br>)<br>AND<br>(<br>("direct composite resin restoration"[tiab] OR "direct resin composite"[tiab] OR "composite resin"[tiab] OR "resin composite"[tiab] OR "nanocomposite"[tiab] OR "bulk-fill composite"[tiab] OR "microhybrid composite"[tiab] OR "nanohybrid composite"[tiab] OR "flowable composite"[tiab] OR "packable composite"[tiab]<br>OR "resin-modified glass ionomer"[tiab] OR RMGIC[tiab] OR RMGI[tiab] OR "glass ionomer"[tiab] OR "glass ionomer cement"[tiab]<br>OR "Dental Restoration, Permanent"[MeSH] OR "Composite Resins"[MeSH] OR "Glass Ionomer Cements"[MeSH])<br>)<br>AND<br>(<br>(etch*[tiab] OR "etch and rinse"[tiab] OR "etch-and-rinse"[tiab] OR "total etch*"[tiab] OR "selective etch*"[tiab] OR "self-etch*"[tiab]<br>OR "phosphoric acid"[tiab] OR "phosphoric acid etch*"[tiab]<br>OR adhesive*[tiab] OR "universal adhesive*"[tiab] OR "multi-mode adhesive*"[tiab] OR "bonding agent*"[tiab] OR "adhesive system*"[tiab]<br>OR "Acids, Phosphoric"[MeSH] OR "Etching"[MeSH] OR "Dental Bonding"[MeSH])<br>)<br>AND<br>(<br>(retention[tiab] OR "retention rate"[tiab] OR debond*[tiab] OR "loss of restoration"[tiab]<br>OR "marginal adaptation"[tiab] OR "marginal integrity"[tiab] OR "marginal discoloration"[tiab]<br>OR fracture[tiab] OR "restoration fracture"[tiab] OR "fracture of the restoration"[tiab]<br>OR "United States Public Health Service"[tiab] OR USPHS[tiab] OR FDI[tiab] OR "FDI criteria"[tiab]<br>OR "Treatment Outcome"[MeSH] OR "Dental Restoration Failure"[MeSH])<br>)<br>AND<br>(<br>randomized[tiab] OR randomised[tiab] OR randomly[tiab] OR RCT[tiab]<br>OR "randomized controlled trial"[pt] OR "controlled clinical trial"[pt] OR "clinical trial"[pt]<br>OR "Randomized Controlled Trials as Topic"[MeSH] OR "Clinical Trials as Topic"[MeSH]

	)
Scopus	TITLE-ABS-KEY(<br>"noncarious cervical lesion" OR "non-carious cervical lesion" OR NCCL OR<br>"cervical lesion" OR "cervical lesions" OR "cervical abrasion" OR<br>"cervical erosion" OR "cervical abfraction"<br>)<br>AND<br>TITLE-ABS-KEY(<br>"direct composite resin restoration" OR "direct resin composite" OR<br>"composite resin" OR "resin composite" OR nanocomposite OR<br>"bulk-fill composite" OR "microhybrid composite" OR<br>"nanohybrid composite" OR "flowable composite" OR "packable composite" OR<br>"resin-modified glass ionomer" OR RMGIC OR RMGI OR<br>"glass ionomer" OR "glass ionomer cement"<br>)<br>AND<br>TITLE-ABS-KEY(<br>etch* OR "etch and rinse" OR "etch-and-rinse" OR<br>"total etch*" OR "selective etch*" OR "self-etch*" OR<br>"phosphoric acid" OR "phosphoric acid etch*" OR<br>adhesive* OR "universal adhesive*" OR "multi-mode adhesive*" OR<br>"bonding agent*" OR "adhesive system*"<br>)<br>AND<br>TITLE-ABS-KEY(<br>retention OR "retention rate" OR debond* OR "loss of restoration" OR<br>"marginal adaptation" OR "marginal integrity" OR "marginal discoloration" OR<br>fracture OR "restoration fracture" OR<br>USPHS OR FDI OR "FDI criteria"<br>)<br>AND<br>TITLE-ABS-KEY(<br>randomized OR randomised OR randomly OR RCT OR<br>"randomized controlled trial" OR "controlled clinical trial" OR<br>"clinical trial"<br>)
Embase	(('noncarious cervical lesion':ti,ab,kw OR 'non-carious cervical lesion':ti,ab,kw OR 'nccl':ti,ab,kw OR 'cervical lesion':ti,ab,kw OR 'cervical lesions':ti,ab,kw OR 'cervical abrasion':ti,ab,kw OR 'cervical erosion':ti,ab,kw OR 'cervical abfraction':ti,ab,kw OR 'dental abrasion'/exp OR 'dental erosion'/exp)) AND (('direct composite resin restoration':ti,ab,kw OR 'direct resin composite':ti,ab,kw OR 'composite resin':ti,ab,kw OR 'resin composite':ti,ab,kw OR 'nanocomposite':ti,ab,kw OR 'bulk-fill composite':ti,ab,kw OR 'microhybrid composite':ti,ab,kw OR 'nanohybrid composite':ti,ab,kw OR 'flowable composite':ti,ab,kw OR 'packable composite':ti,ab,kw OR 'resin-modified glass ionomer':ti,ab,kw OR 'rmgic':ti,ab,kw OR 'rmgi':ti,ab,kw OR 'glass ionomer':ti,ab,kw OR 'glass ionomer cement':ti,ab,kw OR 'dental restoration'/exp OR 'resin'/exp OR 'glass ionomer'/exp)) AND (('etch*':ti,ab,kw OR 'etch and rinse':ti,ab,kw OR 'etch-

	and-rinse':ti,ab,kw OR 'total etch*':ti,ab,kw OR 'selective etch*':ti,ab,kw OR 'self-etch*':ti,ab,kw OR 'phosphoric acid':ti,ab,kw OR 'phosphoric acid etch*':ti,ab,kw OR 'adhesive*':ti,ab,kw OR 'universal adhesive*':ti,ab,kw OR 'multi-mode adhesive*':ti,ab,kw OR 'bonding agent*':ti,ab,kw OR 'adhesive system*':ti,ab,kw OR 'acids, phosphoric'/exp OR 'etching'/exp OR 'dental bonding'/exp)) AND (('retention':ti,ab,kw OR 'retention rate':ti,ab,kw OR 'debond*':ti,ab,kw OR 'loss of restoration':ti,ab,kw OR 'marginal adaptation':ti,ab,kw OR 'marginal integrity':ti,ab,kw OR 'marginal discoloration':ti,ab,kw OR 'fracture':ti,ab,kw OR 'restoration fracture':ti,ab,kw OR 'fracture of the restoration':ti,ab,kw OR 'united states public health service':ti,ab,kw OR 'usphs':ti,ab,kw OR 'fdi':ti,ab,kw OR 'fdi criteria':ti,ab,kw OR 'treatment outcome'/exp OR 'dental restoration failure'/exp)) AND ('randomized':ti,ab,kw OR 'randomised':ti,ab,kw OR 'randomly':ti,ab,kw OR 'rct':ti,ab,kw OR 'randomized controlled trial':it OR 'controlled clinical trial':it OR 'clinical trial':it OR 'randomized controlled trial (topic)'/exp OR 'clinical trial (topic)'/exp)
WoS	TS=(<br>("noncarious cervical lesion" OR "non-carious cervical lesion" OR NCCL OR<br>"cervical lesion" OR "cervical lesions" OR "cervical abrasion" OR<br>"cervical erosion" OR "cervical abfraction")<br>)<br>AND<br>TS=(<br>"direct composite resin restoration" OR "direct resin composite" OR<br>"composite resin" OR "resin composite" OR nanocomposite OR<br>"bulk-fill composite" OR "microhybrid composite" OR<br>"nanohybrid composite" OR "flowable composite" OR "packable composite" OR<br>"resin-modified glass ionomer" OR RMGIC OR RMGI OR<br>"glass ionomer" OR "glass ionomer cement"<br>)<br>AND<br>TS=(<br>etch* OR "etch and rinse" OR "etch-and-rinse" OR<br>"total etch*" OR "selective etch*" OR "self-etch*" OR<br>"phosphoric acid" OR "phosphoric acid etch*" OR<br>adhesive* OR "universal adhesive*" OR "multi-mode adhesive*" OR<br>"bonding agent*" OR "adhesive system*"<br>)<br>AND<br>TS=(<br>retention OR "retention rate" OR debond* OR "loss of restoration" OR<br>"marginal adaptation" OR "marginal integrity" OR "marginal discoloration" OR<br>fracture OR "restoration fracture" OR<br>USPHS OR FDI OR "FDI criteria"<br>)<br>AND<br>TS=(<br>randomized OR randomised OR randomly OR RCT OR<br>"randomized controlled trial" OR "controlled clinical trial" OR

	"clinical trial"<br>)
Cochrane Library	(<br>"noncarious cervical lesion" OR "non-carious cervical lesion" OR NCCL OR<br>"cervical abrasion" OR "cervical erosion" OR "cervical abfraction"<br>)<br>AND<br>(<br>"direct composite resin restoration" OR "direct resin composite" OR<br>"composite resin" OR "resin composite" OR<br>"resin-modified glass ionomer" OR RMGIC OR RMGI OR<br>"glass ionomer cement"<br>)<br>AND<br>(<br>etch* OR "etch and rinse" OR "etch-and-rinse" OR<br>"selective etch*" OR "self-etch*" OR<br>"phosphoric acid" OR adhesive* OR<br>"universal adhesive*" OR "bonding agent*"<br>)<br>AND<br>(<br>retention OR "retention rate" OR debond* OR<br>"marginal adaptation" OR "marginal integrity" OR<br>fracture OR "restoration fracture" OR<br>USPHS OR FDI<br>)<br>AND<br>(<br>randomized OR randomised OR randomly OR RCT OR<br>"controlled clinical trial"<br>)
