# Supplementary material for: Comparison of Adhesive Strategies with Different Etching Approaches on the Clinical Performance of Restorations in Non-Carious Cervical Lesions: A Systematic Review and Network Meta-Analysis
Source: J Funct Biomater. 2026 Mar 25;17(4):160. doi: 10.3390/jfb17040160 (PMC13117247; doi:10.3390/jfb17040160)
Supplement: Supplementary file 1 [file jfb-17-00160-s001.zip › jfb-4192088-Supplementary File S2. References.pdf]

Supplementary File S2. **Full Reference List of Included Studies and Studies Excluded after Full-Text Assessment with Reasons.**

Reports excluded due to insufficient outcome reporting

van Dijken J, Pallesen U. Long-term dentin retention of etch-and-rinse and self-etch adhesives and a resin-modified glass ionomer cement in non-carious cervical lesions. Dent Mater. 2008;24(7):915–22. doi: https://doi.org/10.1016/j.dental.2007.11.008.

van Dijken J, Sunnegårdh-Grönberg K, Lindberg A. Clinical long-term retention of etch-and-rinse and self-etch adhesive systems in non-carious cervical lesions. A 13 years evaluation. Dent Mater. 2007;23(9):1101–7. doi: https://doi.org/10.1016/j.dental.2006.10.005.

Reports excluded due to wrong comparison

de Oliveira FG, Machado LS, Rocha EP, de Alexandre RS, Briso AL, Sundefeld MLM, et al. Clinical evaluation of a composite resin and a resin-modified glass-ionomer cement in non-carious cervical lesions: One-year results. International Journal of Clinical Dentistry. 2012;5(2):155–66.

Ermiş R. Two-year clinical evaluation of four polyacid-modified resin composites and a resin-modified glass-ionomer cement in Class V lesions. Quintessence Int. 2002;33(7):542–8.

Hosaka K, Kubo S, Tichý A, Ikeda M, Shinkai K, Maseki T, et al. Clinical effectiveness of direct resin composite restorations bonded using one-step or two-step self-etch adhesive systems: A three-year multicenter study. Dental Materials Journal. 2021;40(5):1151–9. doi: https://doi.org/10.4012/dmj.2020-428.

Türkün L. The clinical performance of one- and two-step self-etching adhesive systems at one year. The Journal of the American Dental Association. 2005;136(5):656–64; quiz 83. doi: https://doi.org/10.14219/jada.archive.2005.0239.

Vural U, Meral E, Ergin E, Gurgan S, Vural UK, Meral E, et al. Sixty-month comperative evaluation of a glass hybrid restorative and a composite resin in non-carious cervical lesions of bruxist individuals. CLINICAL ORAL INVESTIGATIONS. 2024;28(3). doi: https://doi.org/10.1007/s00784-024-05570-0.

Reports excluded due to wrong intervention

Folwaczny M, Mehl A, Kunzelmann K-H, Hickel R. Clinical performance of a resin-modified glass-ionomer and a compomer in restoring non-carious cervical lesions. 5-year results. American Journal of Dentistry. 2001;14(3):153–6.

Loguercio A, Luque-Martinez I, Fuentes S, Reis A, Muñoz M. Effect of dentin roughness on the adhesive performance in non-carious cervical lesions: A double-blind randomized clinical trial. Journal of dentistry. 2018;69:60–9. doi: https://doi.org/10.1016/j.jdent.2017.09.011.

Omoto É, Dos Santos P, Shinohara M, de Andrade Carvalho P, Catelan A, Fagundes T. Clinical performance of different adhesion strategies in non-carious cervical lesion restorations: A four-year randomized clinical trial. Journal of dentistry. 2025;153:105529. doi: https://doi.org/10.1016/j.jdent.2024.105529.

Pollington S, van Noort R. A clinical evaluation of a resin composite and a compomer in non-carious Class V lesions. A 3-year follow-up. Am J Dent. 2008;21(1):49–52.

Reis A, Mânica D, Ferneda F, Amaral R, Stanislawczuk R, Manso A, et al. A 24-month randomized clinical trial of a two- and three-step etch-and-rinse technique. Am J Dent. 2010;23(4):231–6.

Shinohara MS, de Andrade Carvalho PRM, Marcon LN, Gonçalves DFM, de Souza e Silva Ramos F, Fagundes TC. Randomized clinical trial of different adhesion strategies in noncarious cervical lesion restorations: 1-year follow-up. Quintessence Int. 2020;51(5):352–63. doi: https://doi.org/10.3290/j.qi.a44367.

Reports excluded due to use of an inappropriate restorative material

Çelik E, Tunac A, Yilmaz F. A Randomized, Controlled, Split-mouth Trial Evaluating the Clinical Performance of High-viscosity Glass-ionomer Restorations in Noncarious Cervical Lesions: Two-year Results. The journal of adhesive dentistry. 2018;20(4):299–305. doi: https://doi.org/10.3290/j.jad.a40985.

Studies included

de Carvalho LD, Gondo R, Lopes GC. One-year Clinical Evaluation of Resin Composite Restorations of Noncarious Cervical Lesions in Smokers. Journal of Adhesive Dentistry. 2015;17(5):405–11. doi: https://doi.org/10.3290/j.jad.a35009.

Peumans M, De Munck J, Van Landuyt K, Van Meerbeek B. Thirteen-year randomized controlled clinical trial of a two-step self-etch adhesive in non-carious cervical lesions. Dental Materials. 2015;31(3):308–14. doi: https://doi.org/10.1016/j.dental.2015.01.005.

Abdalla A, García-Godoy F. Clinical evaluation of self-etch adhesives in Class V non-carious lesions. Am J Dent. 2006;19(5):289–92.

Atalay C, Ozgunaltay G, Yazici A, Atalay C, Ozgunaltay G, Yazici AR. Thirty-six-month clinical evaluation of different adhesive strategies of a universal adhesive. CLINICAL ORAL INVESTIGATIONS. 2020;24(4):1569–78. doi: https://doi.org/10.1007/s00784-019-03052-2.

Barceleiro M, Lopes L, Tardem C, Calazans F, Matos T, Reis A, et al. Thirty-six-month follow-up of cervical composite restorations placed with an MDP-free universal adhesive system using different adhesive protocols: a randomized clinical trial. Clin Oral Investig. 2022;26(6):4337–50. doi: https://doi.org/10.1007/s00784-022-04397-x.

Bhat P, Patil SA, Thimmaiah C, Shetty K, Thomas NA, Jayalakshmi PA. Evaluation of Efficacy of Self-etch Adhesives in the Restoration of Noncarious Cervical Lesions: A Controlled Clinical Trial. World Journal of Dentistry. 2024;15(2):95–101. doi: https://doi.org/10.5005/jp-journals-10015-2376.

Boushell L, Heymann H, Ritter A, Sturdevant J, Swift EJ, Wilder AJ, et al. Six-year clinical performance of etch-and-rinse and self-etch adhesives. Dental materials : official publication of the Academy of Dental Materials. 2016;32(9):1065–72. doi: https://doi.org/10.1016/j.dental.2016.06.003.

Brackett W, Brackett M, Dib A, Franco G, Estudillo H. Eighteen-month clinical performance of a self-etching primer in unprepared class V resin restorations. Oper Dent. 2005;30(4):424–9.

Brackett W, Dib A, Brackett M, Reyes A, Estrada B. Two-year clinical performance of Class V resin-modified glass-lonomer and resin composite restorations. Oper Dent. 2003;28(5):477–81.

Burgess J, Sadid-Zadeh R, Cakir D, Ramp L. Clinical evaluation of self-etch and total-etch adhesive systems in noncarious cervical lesions: a two-year report. Operative dentistry. 2013;38(5):477–87. doi: https://doi.org/10.2341/12-355-CR.

Burrow M, Tyas M. Clinical evaluation of three adhesive systems for the restoration of non-carious cervical lesions. Oper Dent. 2007;32(1):11–5. doi: https://doi.org/10.2341/06-50.

Çelik E, Aka B, Yilmaz F. Six-month Clinical Evaluation of a Self-adhesive Flowable Composite in Noncarious Cervical Lesions. J Adhes Dent. 2015;17(4):361–8. doi: https://doi.org/10.3290/j.jad.a34556.

Cruz J, Silva A, Eira R, Coito C, Sousa B, Lopes M, et al. 24-Month Clinical Performance of a Universal Adhesive on Non-Carious Cervical Lesions: Self-Etch and Etch-and Rinse Techniques. JOURNAL OF ADHESIVE DENTISTRY. 2021;23(5):379–87. doi: https://doi.org/10.3290/j.jad.b2000173.

Dalton Bittencourt D, Ezecelevski I, Reis A, Van Dijken J, Loguercio A. An 18-months' evaluation of self-etch and etch & rinse adhesive in non-carious cervical lesions. Acta odontologica Scandinavica. 2005;63(3):173–8. doi: https://doi.org/10.1080/00016350510019874.

de Albuquerque E, Warol F, Calazans F, Poubel L, Marins S, Matos T, et al. A New Dual-cure Universal Simplified Adhesive: 18-month Randomized Multicenter Clinical Trial. Operative dentistry. 2020;45(5):E255–E70. doi: https://doi.org/10.2341/19-144-C.

de Albuquerque E, Warol F, Tardem C, Calazans F, Poubel L, Matos T, et al. Universal Simplified Adhesive applied under different bonding technique's: 36-month Randomized Multicentre Clinical Trial. Journal of dentistry. 2022;122:104120. doi: https://doi.org/10.1016/j.jdent.2022.104120.

de Almeida R, Lima S, Nassif M, Mattos N, de Matos T, de Jesus Tavarez R, et al. Eighteen-month clinical evaluation of a new universal adhesive applied in the "no-waiting" technique: a randomized clinical trial. Clinical oral investigations. 2023;27(1):151–63. doi: https://doi.org/10.1007/s00784-022-04703-7.

de Almeida R, Siqueira F, Cantanhede L, Carrillo K, Carpio-Salvatierra B, Ñaupari-Villasante R, et al. Thirty-six months of clinical evaluation of a new universal adhesive applied in the "no waiting" technique: A randomized clinical trial. J Dent. 2026;164:106222. doi: https://doi.org/10.1016/j.jdent.2025.106222.

de Paris Matos T, Perdigão J, de Paula E, Coppla F, Hass V, Scheffer R, et al. Five-year clinical evaluation of a universal adhesive: A randomized double-blind trial. Dental materials : official publication of the Academy of Dental Materials. 2020;36(11):1474–85. doi: https://doi.org/10.1016/j.dental.2020.08.007.

Digole V, Warhadpande M, Dua P, Dakshindas D. Comparative evaluation of clinical performance of two self-etch adhesive systems with total-etch adhesive system in noncarious cervical lesions: An in vivo study. Journal of Conservative Dentistry. 2020;23(2):190–5. doi: https://doi.org/10.4103/JCD.JCD_166_20.

Dutra-Correa M, Kiyan V, Ciaramicoli M, Pecorari V, Rodrigues F, Coury Saraceni C. Randomized clinical trial of four adhesion strategies: A 42 month study. Indian journal of dental research : official publication of Indian Society for Dental Research. 2019;30(4):487–95. doi: https://doi.org/10.4103/ijdr.IJDR_466_16.

Ermis R, Van Landuyt K, Cardoso M, De Munck J, Van Meerbeek B, Peumans M. Clinical effectiveness of a one-step self-etch adhesive in non-carious cervical lesions at 2 years. Clinical oral investigations. 2012;16(3):889–97. doi: https://doi.org/10.1007/s00784-011-0565-4.

Follak A, Ilha B, Oling J, Savian T, Rocha R, Soares F. Clinical behavior of universal adhesives in non-carious cervical lesions: A randomized clinical trial. Journal of dentistry. 2021;113:103747. doi: https://doi.org/10.1016/j.jdent.2021.103747.

Franco E, Benetti A, Ishikiriama S, Santiago S, Lauris J, Jorge M, et al. 5-year clinical performance of resin composite versus resin modified glass ionomer restorative system in

non-carious cervical lesions. Oper Dent. 2006;31(4):403–8. doi: https://doi.org/10.2341/05-87.

Fron H, Vergnes J, Moussally C, Cazier S, Simon A, Chieze J, et al. Effectiveness of a new one-step self-etch adhesive in the restoration of non-carious cervical lesions: 2-year results of a randomized controlled practice-based study. Dental materials : official publication of the Academy of Dental Materials. 2011;27(3):304–12. doi: https://doi.org/10.1016/j.dental.2010.11.006.

Fuentes M, Perdigão J, Baracco B, Giráldez I, Ceballos L. Effect of an additional bonding resin on the 5-year performance of a universal adhesive: a randomized clinical trial. Clinical oral investigations. 2023;27(2):837–48. doi: https://doi.org/10.1007/s00784-022-04613-8.

Gallo J, Burgess J, Ripps A, Walker R, Ireland E, Mercante D, et al. Three-year clinical evaluation of a compomer and a resin composite as Class V filling materials. Oper Dent. 2005;30(3):275–81.

Haak R, Hähnel M, Schneider H, Rosolowski M, Park K, Ziebolz D, et al. Clinical and OCT outcomes of a universal adhesive in a randomized clinical trial after 12 months. Journal of dentistry. 2019;90:103200. doi: https://doi.org/10.1016/j.jdent.2019.103200.

Haak R, Werner MS, Schneider H, Häfer M, Schulz-Kornas E. Clinical Outcomes and Quantitative Margin Analysis of a Universal Adhesive Using a Randomized Clinical Trial over Three Years. J Clin Med. 2022;11(23). doi: https://doi.org/10.3390/jcm11236910

Häfer M, Jentsch H, Haak R, Schneider H. A three-year clinical evaluation of a one-step self-etch and a two-step etch-and-rinse adhesive in non-carious cervical lesions. Journal of dentistry. 2015;43(3):350–61. doi: https://doi.org/10.1016/j.jdent.2014.12.009.

Jassal M, Mittal S, Tewari S. Clinical Effectiveness of a Resin-modified Glass Ionomer Cement and a Mild One-step Self-etch Adhesive Applied Actively and Passively in Noncarious Cervical Lesions: An 18-Month Clinical Trial. Oper Dent. 2018;43(6):581–92. doi: https://doi.org/10.2341/17-147-c.

Kemaloğlu H, Atalayin Ozkaya C, Ergucu Z, Onal B. Follow-up of flowable resin composites performed with a universal adhesive system in non-carious cervical lesions: A randomized, controlled 24-month clinical trial. American journal of dentistry. 2020;33(1):39–42.

Kubo S, Kawasaki K, Yokota H, Hayashi Y. Five-year clinical evaluation of two adhesive systems in non-carious cervical lesions. Journal of dentistry. 2006;34(2):97–105. doi: https://doi.org/10.1016/j.jdent.2005.04.003.

Lawson N, Robles A, Fu C, Lin C, Sawlani K, Burgess J. Two-year clinical trial of a universal adhesive in total-etch and self-etch mode in non-carious cervical lesions. Journal of dentistry. 2015;43(10):1229–34. doi: https://doi.org/10.1016/j.jdent.2015.07.009.

Loguercio A, Bittencourt D, Baratieri L, Reis A. A 36-month evaluation of self-etch and etch-and-rinse adhesives in noncarious cervical lesions. Journal of the American Dental Association (1939). 2007;138(4):507–14; quiz 35–7. doi: https://doi.org/10.14219/jada.archive.2007.0204.

Loguercio A, de Paula E, Hass V, Luque-Martinez I, Reis A, Perdigão J. A new universal simplified adhesive: 36-Month randomized double-blind clinical trial. Journal of dentistry. 2015;43(9):1083–92. doi: https://doi.org/10.1016/j.jdent.2015.07.005.

Loguercio A, Reis A. Application of a dental adhesive using the self-etch and etch-and-rinse approaches: an 18-month clinical evaluation. Journal of the American Dental Association (1939). 2008;139(1):53–61. doi: https://doi.org/10.14219/jada.archive.2008.0021.

Loguercio A, Reis A, Barbosa A, Roulet J. Five-year double-blind randomized clinical evaluation of a resin-modified glass ionomer and a polyacid-modified resin in noncarious cervical lesions. J Adhes Dent. 2003;5(4):323–32.

Lopes L, Calazans F, Hidalgo R, Buitrago L, Gutierrez F, Reis A, et al. Six-month Follow-up of Cervical Composite Restorations Placed With a New Universal Adhesive System: A Randomized Clinical Trial. Operative dentistry. 2016;41(5):465–80. doi: https://doi.org/10.2341/15-309-C.

Manarte-Monteiro P, Domingues J, Teixeira L, Gavinha S, Manso MC. Universal adhesives and adhesion modes in non-carious cervical restorations: 2-year randomised clinical trial. Polymers. 2022;14(1). doi: https://doi.org/10.3390/polym14010033.

Merle C, Fortenbacher M, Schneider H, Schmalz G, Challakh N, Park K, et al. Clinical and OCT assessment of application modes of a universal adhesive in a 12-month RCT. Journal of dentistry. 2022;119:104068. doi: https://doi.org/10.1016/j.jdent.2022.104068.

Moosavi H, Kimyai S, Forghani M, Khodadadi R. The clinical effectiveness of various adhesive systems: an 18-month evaluation. Oper Dent. 2013;38(2):134–41. doi: https://doi.org/10.2341/12-110-cr.

Ñaupari-Villasante R, Carpio-Salvatierra B, Matos TP, Tardem C, Calazans FS, Binz Ordóñez MCR, et al. Longevity of a single-dose, dual-cure universal adhesive: A 7.5-year double-blind split-mouth two-center randomized trial. Dent Mater. 2025. doi: https://doi.org/10.1016/j.dental.2025.11.015

Ñaupari-Villasante R, Matos T, de Albuquerque E, Warol F, Tardem C, Calazans F, et al. Five-year clinical evaluation of universal adhesive applied following different bonding techniques: A randomized multicenter clinical trial. Dental materials : official publication of the Academy of Dental Materials. 2023;39(6):586–94. doi: https://doi.org/10.1016/j.dental.2023.04.007.

Onal B, Pamir T. The two-year clinical performance of esthetic restorative materials in noncarious cervical lesions. J Am Dent Assoc. 2005;136(11):1547–55. doi: https://doi.org/10.14219/jada.archive.2005.0085.

Oz F, Dursun M, Ergin E. Clinical Performance of a "No Wait" Universal Adhesive in Noncarious Cervical Lesions: A Two-year Randomized Controlled Clinical Trial. The journal of adhesive dentistry. 2022;24:313–23. doi: https://doi.org/10.3290/j.jad.b3240675.

Oz F, Ergin E, Canatan S. Twenty-four-month clinical performance of different universal adhesives in etch-and-rinse, selective etching and self-etch application modes in NCCL - a randomized controlled clinical trial. Journal of applied oral science : revista FOB. 2019;27:e20180358. doi: https://doi.org/10.1590/1678-7757-2018-0358.

Ozel E, Say E, Yurdaguven H, Soyman M. One-year clinical evaluation of a two-step self-etch adhesive with and without additional enamel etching technique in cervical lesions. Aust Dent J. 2010;55(2):156–61. doi: https://doi.org/10.1111/j.1834-7819.2010.01218.x.

Pappa E, Gkavela G, Sampri I, Masouras K, Rahiotis C, Kakaboura A. A 2-Year Randomized Clinical Trial of Three Bonding Techniques in Non-Carious Cervical Lesions. Medicina (Kaunas, Lithuania). 2024;60(6). doi: https://doi.org/10.3390/medicina60061005.

Pena C, Rodrigues J, Ely C, Giannini M, Reis A. Two-year Randomized Clinical Trial of Self-etching Adhesives and Selective Enamel Etching. Operative dentistry. 2016;41(3):249–57. doi: https://doi.org/10.2341/15-130-C.

Perdigão J, Carmo A, Anauate-Netto C, Amore R, Lewgoy H, Cordeiro H, et al. Clinical performance of a self-etching adhesive at 18 months. Am J Dent. 2005;18(2):135–40.

Perdigão J, Ceballos L, Giráldez I, Baracco B, Fuentes M. Effect of a hydrophobic bonding resin on the 36-month performance of a universal adhesive-a randomized clinical trial. Clinical oral investigations. 2020;24(2):765–76. doi: https://doi.org/10.1007/s00784-019-02940-x.

Perdigão J, Dutra-Corrêa M, Saraceni C, Ciaramicoli M, Kiyan V, Queiroz C. Randomized clinical trial of four adhesion strategies: 18-month results. Oper Dent. 2012;37(1):3–11. doi: https://doi.org/10.2341/11-222-c.

Perdigão J, Dutra-Corrêa M, Saraceni S, Ciaramicoli M, Kiyan V. Randomized clinical trial of two resin-modified glass ionomer materials: 1-year results. Oper Dent. 2012;37(6):591–601. doi: https://doi.org/10.2341/11-415-c.

Perdigão J, Kose C, Mena-Serrano A, De Paula E, Tay L, Reis A, et al. A new universal simplified adhesive: 18-month clinical evaluation. Oper Dent. 2014;39(2):113–27. doi: https://doi.org/10.2341/13-045-c.

Peumans M, Munck J, Van Landuyt K, Lambrechts P, Van Meerbeek B. Three-year clinical effectiveness of a two-step self-etch adhesive in cervical lesions. European journal of oral sciences. 2005;113(6):512–8. doi: https://doi.org/10.1111/j.1600-0722.2005.00256.x.

Peumans M, Vandormael S, De Coster I, De Munck J, Van Meerbeek B. Three-year Clinical Performance of a Universal Adhesive in Non-Carious Cervical Lesions. The journal of adhesive dentistry. 2023;25:133–46. doi: https://doi.org/10.3290/j.jad.b4186751.

Peumans M, Vandormael S, Heeren A, De Munck J, Van Meerbeek B. Six-year Clinical Performance of a 2-step Self-etch Adhesive in Noncarious Cervical Lesions. The journal of adhesive dentistry. 2021;23(3):201–15. doi: https://doi.org/10.3290/j.jad.b1367831.

Peumans M, Wouters L, De Munck J, Van Meerbeek B, Van Landuyt K. Nine-year Clinical Performance of a HEMA-free One-step Self-etch Adhesive in Noncarious Cervical Lesions. The journal of adhesive dentistry. 2018;20(3):195–203. doi: https://doi.org/10.3290/j.jad.a40630.

Ranjitha G, Vikram R, Meena N, Vijayalakshmi L, Murthy C. Clinical efficacy of universal adhesives for the restoration of noncarious cervical lesions: A randomized clinical trial. Journal of conservative dentistry : JCD. 2020;23(3):227–32. doi: https://doi.org/10.4103/JCD.JCD_51_20.

Ruschel V, Shibata S, Stolf S, Chung Y, Baratieri L, Heymann H, et al. Eighteen-month Clinical Study of Universal Adhesives in Noncarious Cervical Lesions. Operative dentistry. 2018;43(3):241–9. doi: https://doi.org/10.2341/16-320-C.

Ruschel V, Stolf S, da Luz Baratieri C, Chung Y, Boushell L, Baratieri L, et al. Five-year Clinical Evaluation of Universal Adhesives in Noncarious Cervical Lesions. Operative dentistry. 2023;48(4):364–72. doi: https://doi.org/10.2341/21-132-C.

Ruschel V, Stolf S, Shibata S, Chung Y, Boushell L, Baratieri L, et al. Three-year clinical evaluation of universal adhesives in non-carious cervical lesions. American journal of dentistry. 2019;32(5):223–8.

Santiago S, Passos V, Vieira A, Navarro M, Lauris J, Franco E. Two-year clinical evaluation of resinous restorative systems in non-carious cervical lesions. Braz Dent J. 2010;21(3):229–34. doi: https://doi.org/10.1590/s0103-64402010000300010.

Schwendicke F, Müller A, Seifert T, Jeggle-Engbert L, Paris S, Göstemeyer G, et al. Glass hybrid versus composite for non-carious cervical lesions: Survival, restoration quality and costs in randomized controlled trial after 3 years. JOURNAL OF DENTISTRY. 2021;110. doi: https://doi.org/10.1016/j.jdent.2021.103689 EA MAY 2021 EY 2021.

Tian F, Wang X, Gao X. [Clinical evaluation of a two-step etch-and-rinse adhesive and a one-step self-etch adhesive in non-carious cervical lesion]. Beijing Da Xue Xue Bao Yi Xue Ban. 2014;46(1):58–61.

Tuncer D, Yazici A, Özgünaltay G, Dayangac B. Clinical evaluation of different adhesives used in the restoration of non-carious cervical lesions: 24-month results. Aust Dent J. 2013;58(1):94–100. doi: https://doi.org/10.1111/adj.12028.

van Dijken J. A randomized controlled 5-year prospective study of two HEMA-free adhesives, a 1-step self etching and a 3-step etch-and-rinse, in non-carious cervical lesions. Dental materials : official publication of the Academy of Dental Materials. 2013;29(11):e271–80. doi: https://doi.org/10.1016/j.dental.2013.08.203.

Van Landuyt K, Peumans M, De Munck J, Cardoso M, Ermis B, Van Meerbeek B. Three-year clinical performance of a HEMA-free one-step self-etch adhesive in non-carious cervical lesions. European journal of oral sciences. 2011;119(6):511–6. doi: https://doi.org/10.1111/j.1600-0722.2011.00855.x.

van Meerbeek B, Kanumilli P, de Munck J, van Landuyt K, Lambrechts P, Peumans M. A randomized controlled study evaluating the effectiveness of a two-step self-etch adhesive with and without selective phosphoric-acid etching of enamel. Dental Materials. 2005;21(4):375–83. doi: https://doi.org/10.1016/j.dental.2004.05.008.

Vural U, Meral E, Ergin E, Gürgan S, Koc Vural U, Meral E, et al. Twenty-four-month clinical performance of a glass hybrid restorative in non-carious cervical lesions of patients with bruxism: a split-mouth, randomized clinical trial. CLINICAL ORAL INVESTIGATIONS. 2020;24(3):1229–38. doi: https://doi.org/10.1007/s00784-019-02986-x.

Yaman B, Doğruer I, Gümüştaş B, Efes B. Three-year randomized clinical evaluation of a low-shrinkage silorane-based resin composite in non-carious cervical lesions. Clinical oral investigations. 2014;18(4):1071–9. doi: https://doi.org/10.1007/s00784-013-1079-z.

Zanatta R, Silva T, Esper M, Bresciani E, Gonçalves S, Caneppele T. Bonding Performance of Simplified Adhesive Systems in Noncarious Cervical Lesions at 2-year Follow-up: A Double-blind Randomized Clinical Trial. Operative dentistry. 2019;44(5):476–87. doi: https://doi.org/10.2341/18-049-C.

Studie included from supplementary retrieval

Peumans M, De Munck J, Van Landuyt K, Poitevin A, Lambrechts P, Van Meerbeek B. Eight-year clinical evaluation of a 2-step self-etch adhesive with and without selective enamel etching. Dental materials : official publication of the Academy of Dental Materials. 2010;26(12):1176–84. doi: https://doi.org/10.1016/j.dental.2010.08.190.
