# Supplementary material for: Comparison of Adhesive Strategies with Different Etching Approaches on the Clinical Performance of Restorations in Non-Carious Cervical Lesions: A Systematic Review and Network Meta-Analysis
Source: J Funct Biomater. 2026 Mar 25;17(4):160. doi: 10.3390/jfb17040160 (PMC13117247; doi:10.3390/jfb17040160)
Supplement: Supplementary file 1 [file jfb-17-00160-s001.zip › jfb-4192088-Supplementary File S3_Overview.pdf]

Supplementary File S3. Detailed Characteristics and Clinical Outcomes of the Included Randomized Controlled Trials.

Study	Country	Inclusion criteria	Treatment Methods (n)	Follow up	Conclusions
Abdalla_2006	Egypt	Humans with wedge-shaped NCCLs (Class V) in maxillary/mandibular premolars; ≥2 restorations per subject; excluded: compromised medical history, severe/chronic periodontitis, extreme dental caries sensitivity, abnormal occlusion	3 arms: Admira Bond (etch-and-rinse) vs Clearfil SE Bond (2-step self-etch) vs Hybrid Bond (1-step self-etch)	Baseline; 12 months; 24 months.	Admira Bond and Clearfil SE Bond showed excellent 2-year performance; Hybrid Bond deteriorated in marginal adaptation/discoloration; no restorations were lost.
Atalay_2019	Turkey	Adults >18; good general health & oral hygiene; ≥20 teeth under occlusion; ≥3 NCCLs; lesions with Tooth Wear Index score 3–4; ≤50% enamel at cavosurface margin; exclusions: systemic/local disorders preventing treatment, poor oral hygiene, bruxism, severe/chronic periodontitis, non-vital teeth, or other-surface restorations	3 arms (n=55 each at placement): SU selective-etch vs SU etch-and-rinse vs SU self-etch	Baseline; 6; 12; 18; 24; 36 months	All adhesive modes showed similar retention rates; the self-etch mode showed less satisfactory marginal staining/adaptation, though clinically acceptable at 36 months.
Barceleiro_2022	Brazil	Adults (≥18y) in good general health with acceptable oral hygiene and ≥20 teeth under occlusion; required ≥4 NCCLs in four different teeth. Lesions: non-carious, non-retentive, depth >1 mm, involving enamel and dentin of vital teeth without mobility; ≤50% enamel at cavosurface margin. Excluded: extremely poor oral hygiene, severe/chronic periodontitis, heavy bruxism.	4 arms (31 restorations each; N=124; Np=31): ER-D (etch-and-rinse, dry dentin) vs ER-M (etch-and-rinse, moist dentin) vs SE-et (self-etch with selective enamel etching) vs SET (self-etch without selective enamel etching)	Baseline; 6; 18; 36 months	The clinical behavior of the MDP-free universal adhesive depended on bonding strategy, with overall poor performance, particularly in self-etch application; etch-and-rinse showed better retention.
Bhat_2024	India	Adults 20–60y; vital posterior teeth; fair oral hygiene; healthy periodontium; in occlusion with opposing teeth; normal occlusal relationship; NCCL duration <2y; lesion depth 1–2 mm. Excluded: severe periodontal disease, rampant caries, poor oral hygiene, pregnant/lactating, allergy to materials.	3 parallel arms (n=20 restorations/group; total=60): Group I Adper Single Bond 2 total-etch; Group II Clearfil SE (2-step self-etch); Group III G-bond (1-step self-etch). All restored with Filtek Supreme nanocomposite.	Baseline; 6 months; 12 months	Self-etch adhesives (Clearfil SE, G-bond) showed comparable 1-year clinical performance to the etch-and-rinse control; longer follow-up is required.

Boushell_2016	USA	Adults (30–75 years) with notch-shaped non-carious cervical lesions (NCCLs) in permanent teeth; lesions roughened with diamond; no enamel bevel or mechanical retention; informed consent.	Xeno IV self-etch (n=40); Xeno III self-etch (n=39); XP Bond etch-and-rinse (n=41); restorations with TPH3 composite resin.	Baseline, 6, 18, 36, and 72 months	At 6 years, no statistically significant differences among adhesives for retention, marginal discoloration, or marginal adaptation; failures mainly due to loss of retention.
Brackett_2003	Mexico	Human adults with caries-free cervical erosion/abfraction NCCLs in permanent teeth; restorations placed without tooth preparation.	Paired RCT split-mouth: 37 pairs of NCCL restorations in 24 patients; randomized assignment per pair to Fuji II LC vs Z250/Single Bond; outcomes by modified Ryge/USPHS.	Baseline; 6; 12; 18; 24 months	No statistically significant difference in overall clinical performance between materials over 2 years; composite had better color match, while RMGI had higher retention.
Brackett_2005	Mexico	Healthy adults with caries-free cervical erosion/abfraction NCCLs in permanent teeth; lesion axial depth 1–2 mm; no mechanical preparation/abrasion (pumice cleaning only); paired comparable lesions per patient.	Paired split-mouth trial: 38 pairs (n=38 restorations/arm at baseline) in 25 patients; each pair randomized to Tyrian SPE protocol vs acid-etch + One-Step.	Baseline; 6; 12; 18 months	Under this protocol, neither adhesive strategy was effective for retaining unprepared Class V resin composite restorations; no significant difference between strategies.
Burgess_2013	USA	Age ≥19; ≥3 NCCLs (Class V) with depth >1.5 mm; regular attendee/able to return; good general health; no rampant caries; no chronic periodontitis; normal salivary function.	52 patients; 156 NCCL restorations (52 per adhesive). Rubber dam isolation; 0.5-mm enamel bevel; pumice cleaning; adhesives applied per manufacturer; restored with Filtek Supreme Plus (incremental).	Baseline (1 week), 6 months, 12 months, 24 months.	At 24 months, the three adhesives showed acceptable clinical performance with no statistically significant differences among them.
Burrow_2007	Australia	Human patients with NCCLs in permanent teeth; excluded severe periodontal disease/poor gingival health, medical problems preventing attendance, or rampant caries; lesions ~1–3 mm depth.	Randomized consecutive placement in NCCLs: total 92 restorations/20 patients; ~30–31 restorations per material (Fuji II LC n=31; SE	6, 12, 24, 36 months	RM-GIC showed best performance; self-etch primer + resin composite also good; phosphoric acid–etch single-bottle system showed higher early failure and then stabilized.

			Bond/Clearfil ST n=31; Single Bond/Filtek A110 n=30). Recall at 6 months, 1, 2, 3 years.		
Çelik_2015	Turkey	≥2 symmetrical NCCLs; permanent dentition; cervical margins in dentin; antagonist + adjacent tooth; vital pulp and no pulpitis symptoms; available for follow-up; exclusions included severe periodontal/systemic disease, allergy, very poor oral hygiene, pregnancy/breast-feeding, overloading/crowding, severe sclerotic dentin degree 4, orthodontics, fluoride supplements/desensitizers.	Randomized controlled single-center split-mouth trial; 19 patients; 80 NCCLs; 40 restorations/group: SAFC (Fusio Liquid Dentin) vs nanohybrid composite (G-aenial) + Optibond FL (3-step E&R) with selective enamel etching; 1–2 mm enamel bevel; evaluated by FDI criteria.	Baseline (1 week) and 6 months	SAFC showed clinically unacceptable performance at 6 months due to high early retention failures; control arm remained clinically acceptable.
Cruz_2021	Portugal	Adults ≥18y; good general health; acceptable oral hygiene; ≥20 teeth in occlusion; ≥2 NCCLs (incisors/canines/premolars); non-carious, non-retentive, depth >1 mm; involves enamel+dentin; vital teeth; no mobility; cavosurface margin ≤50% enamel; tooth in occlusion and proximal contact; exclusions: severe xerostomia, bruxism, poor oral hygiene, severe/chronic periodontitis, allergies, recent orthodontics, long-term anti-inflammatory/analgesic/psychotropic drugs, abutment teeth for prostheses, symptomatic pathology, smoking.	Randomized double-blind clinical trial; 117 restorations/26 patients: ADH self-etch (n=58) vs ADH etch-and-rinse (n=59); same composite in all arms (Tetric EvoCeram).	Baseline and 24 months	Self-etch mode showed better clinical performance than etch-and-rinse, particularly for fractures/retention at 24 months.
Dalton_2005	Brazil	Healthy; ≥20 teeth; ≥2 pairs of equal-sized NCCLs without undercuts; Duke sclerosis 2–3; all teeth with occlusal contact; ≤50% cavosurface margin in enamel; excluded extremely poor oral hygiene, heavy bruxism, hypersensitivity lesions.	Randomized intra-individual (paired) controlled prospective study; 25 patients; 78 restorations (39/group); no bevel/retention; rubber dam; adhesives per	Baseline, 6, 12, 18 months	Both adhesives met ADA retention acceptance at 18 months; self-etch showed faster progressive marginal degradation.

			manufacturer; Filtek A110 in 1–3 increments.		
deAlbuquerque_2020	Brazil	≥18y; good general health; acceptable oral hygiene; ≥20 teeth in occlusion; ≥4 NCCLs in different teeth; lesions nonretentive, depth >1 mm, enamel+dentin, vital teeth, no mobility; ≤50% enamel at cavosurface margin; exclusions: extremely poor hygiene, orthodontic devices, severe/chronic periodontitis, heavy bruxism, allergy to materials, pregnant/breastfeeding, chronic anti-inflammatory/analgesic/psychotropic drug use.	Multicenter, double-blind, split-mouth RCT; 50 patients; 200 restorations; 4 arms (n=50 restorations/arm): SEE, SET, ERDry, ERWet; all restored with Admira Fusion.	Baseline; 6, 12, 18 months	Clinical performance of Futurabond U was not dependent on bonding strategy up to 18 months; self-etch showed more marginal discrepancy.
deAlbuquerque_2022	Brazil	≥18y; good general health; acceptable oral hygiene; ≥20 teeth in occlusion; ≥4 NCCLs in different teeth; lesions non-retentive, depth >1 mm, enamel+dentin, vital teeth, no mobility; ≤50% enamel at cavosurface margin; exclusions: extremely poor hygiene, orthodontic devices, severe/chronic periodontitis, >10 teeth with occlusal facets, allergy to materials, pregnant/breastfeeding, chronic anti-inflammatory/analgesic/psychotropic drug use.	Multicenter, double-blind, split-mouth RCT; 50 patients; 200 restorations; 4 arms (n=50 restorations/arm): SEE, SET, ERDry, ERWet; all restored with Admira Fusion.	Baseline; 6, 12, 18, 36 months	Clinical performance of Futurabond U did not depend on bonding strategy after 36 months; greater marginal discrepancy observed in self-etch groups.
deAlmeida_2023	Brazil	Good oral and general health; ≥18y; ≥20 teeth under occlusion; ≥4 NCCLs in 4 separate teeth; lesions depth/extent ≥1 mm involving enamel+dentin in vital non-mobile teeth; ≥50% margins devoid of enamel; exclusions: inadequate oral hygiene, severe/chronic periodontitis, xerostomia, braces, heavy bruxism.	Randomized clinical trial; 24 participants; 176 restorations; 4 arms (n=44 restorations/arm): PB-ER, PB-SE, CQ-ER, CQ-SE; two universal adhesives (Prime&Bond Active and Clearfil Universal Bond Quick), each applied in ER or SE strategy; restorations placed with	Baseline; 6 months; 18 months (no 12-month exam due SARS-CoV-2 restrictions)	After 6 and 18 months, CQ applied with the no-waiting technique showed similar clinical performance to PB used with standard application time, across ER and SE strategies.

			Filtek Z-350 XT using incremental technique.		
deAlmeida_2026	Brazil	Good oral and general health; ≥18 years; ≥20 occluded teeth; ≥4 NCCLs on distinct teeth; lesions ≥1 mm depth involving enamel and dentin in stable teeth, with ≥50% of margins lacking enamel; received oral hygiene instructions; exclusions: inadequate oral hygiene, severe/chronic periodontitis, xerostomia, braces, heavy bruxism.	Double-blind split-mouth RCT; 24 participants (12 men/12 women); 176 NCCL restorations (44/group): PBEr, PBSe, CQEr, CQSe; adhesives applied in etch-and-rinse or self-etch modes; restored with Filtek Z-350 XT.	Baseline; 6, 18, 36 months	CQ applied with the "no-waiting" technique showed lower fracture/retention with the self-etch strategy but overall clinical performance comparable to PB; etch-and-rinse yielded fewer marginal discrepancies for both adhesives.
deCarvalho_2015	Brazil	>20 natural teeth; ≥2 cervical NCCLs in different quadrants (1.0–3.5 mm occluso-gingival; <2 mm depth); nonretentive NCCL with <50% margin in enamel and >50% area in dentin; 50% smokers (20–30 cigarettes/day) and 50% non-smokers; Exclusions: medically compromised; severe bruxism; severe tooth sensitivity; active periodontal disease; poor oral hygiene; carious lesions associated with cervical lesions; pregnancy; debilitated (physical/mental); orthodontic treatment or bleaching; nonvital teeth	Randomized double-blind clinical study (bonding strategy); single operator; 26 patients; 152 restorations total; 4 groups (n=38 restorations/group): G1 ER non-smokers; G2 selective enamel etch non-smokers; G3 ER smokers; G4 selective enamel etch smokers; all restored with Filtek Z350 XT	Baseline (1 week); 6 months; 12 months	Neither cigarette smoking habit nor adhesive strategy influenced the clinical performance of resin composite cervical restorations over 12 months.
deParisMatos_2020	Brazil	≥18y; good general health; acceptable oral hygiene; ≥20 teeth under occlusion; ≥4 NCCLs in different teeth; lesions non-carious, non-retentive, depth >1 mm, enamel+dentin, vital teeth, no mobility; ≤50% enamel at cavosurface margin; exclusions: extremely poor hygiene, orthodontic devices, severe/chronic periodontitis, heavy bruxism, allergy to materials, pregnant/breastfeeding,	Randomized, double-blind clinical trial; convenience sample; 39 patients; 200 restorations allocated to 4 groups (n=50 restorations/group): ERm, ERd, SEet, SE; all restored with Filtek Supreme Ultra	Baseline; 6, 18, 36, 60 months	After 5 years, etch-and-rinse performed better than self-etch; selective enamel etching is recommended when using self-etch; FDI and USPHS showed similar results at 5 years.

		chronic anti-inflammatory/analgesic/psychotropic drug use			
Digole_2020	India	Age 20–50y; ≥3 NCCLs; cervico-incisal height 1–2 mm; mean depth ~1 mm; healthy periodontium; antagonist teeth; exclusions: extremely poor oral hygiene, severe/chronic periodontitis, heavy bruxism, nonvital teeth, caries/periapical lesions.	Prospective double-blind randomized controlled clinical trial; within-patient allocation of 3 teeth per patient to 3 groups; 75 restorations total (25/group); single operator; all restored with Filtek Z350XT.	Baseline (1 week); 6 months; 18 months	No significant differences among total-etch and self-etch systems for retention, marginal integrity, and postoperative sensitivity up to 18 months.
DutraCorrea_2019	Brazil	Need of NCCLs; 18–80y; vital teeth; normal occlusion; ≥1 adjacent contact; healthy periodontal tissues; exclusions: caries/fractured teeth, hypersensitivity, worn parafunction facets, noncommitment for recalls, long-term drug therapies/chronic diseases, pregnancy, allergies, orthodontic treatment, indirect restorations, periodontal diseases, lesions with no need for restoration, participation in another trial.	Randomized clinical trial; 39 patients; 125 NCCLs; 4 groups (restorations): MP n=29; SB n=32; SE n=30; EB n=34; all restored with Filtek Supreme Plus; nonbeveled, no grooves; evaluated with UNC-modified USPHS.	Baseline; 6, 18, 36, 42 months	Adhesive strategies showed broadly similar clinical performance up to 42 months for key parameters (including retention and marginal adaptation); EB showed earlier marginal deterioration; wear increased over time for MP.
Ermis_2012	Turkey	Non-hospitalized adults with NCCLs (abrasion/erosion/abfraction) requiring restoration; cervical margin in dentin and incisal margin in enamel; exclusions: complex medical history, severe/chronic periodontitis, extreme caries sensitivity, heavy bruxism (>50% tooth structure lost by attrition), carious lesions.	Randomized controlled prospective Class V trial; 26 patients; 161 NCCLs; 2 arms: Clearfil S3 Bond n=81 restorations; Optibond FL n=80 restorations; all restored with Clearfil AP-X.	Baseline; 6, 12, 24 months	Both adhesives were clinically successful at 2 years; Clearfil S3 Bond and Optibond FL had similar overall success, though marginal integrity degraded over time and Clearfil S3 Bond showed more small enamel marginal defects/discolorations.
Follak_2021	Brazil	≥18y; ASA I–II; ≥20 teeth; ≥1 NCCL on buccal surface of incisors/canines/premolars; lesion depth ≥1 mm; no caries or prior restoration in selected NCCL; exclusions: non-vital tooth or no antagonist, active orthodontics, smokers,	Randomized double-blind (participant/examiner) multi-arm trial; 54 participants; 211 NCCLs; 4 groups: PB-ER n=53, PB-SE n=50, SBU-ER n=54,	Baseline (7 days after placement/finishing); 6 months	Bonding strategy affected Prime & Bond Elect: self-etch showed worse clinical performance; etch-and-rinse recommended for Prime & Bond Elect; Scotchbond Universal performed similarly in ER and SE at 6 months.

		heavy bruxism, poor oral hygiene (GBI>20%), severe/chronic periodontitis.	SBU-SE n=54; all restored with Filtek Z250.		
Franco_2006	Brazil	Adults 18–50y; good oral hygiene; no periodontal disease or deleterious habits; ≥2 NCCLs; lesion depth ≥1 mm.	Prospective clinical trial; 30 patients; 70 restorations; 2 arms: RC n=35 restorations, RMGI n=35 restorations; one operator; no mechanical prep; rubber dam.	Baseline; 6, 12, 24, 60 months	After 5 years, resin-modified glass ionomer restorations showed superior clinical performance vs 1-bottle adhesive/resin composite, mainly due to higher retention.
Fron_2011	France	≥18y; affiliated to social welfare organization; acceptable oral hygiene; ≥2 NCCLs on different teeth; non-carious, >1 mm deep, enamel+dentin; vital incisor/canine/premolar without mobility; exclusions: compromised medical history, severe/active periodontal or caries disease, heavy bruxism/traumatic occlusion.	Practice-based paired-tooth (split-mouth) RCT; 28 patients; 56 NCCL restorations; 2 arms: Bond Force self-etch vs selective enamel etch (40% phosphoric acid) + Bond Force; all restored with Estelite Flow Quick (flowable) + Estelite Sigma Quick (microhybrid).	Baseline; 6, 12, 24 months	After 2 years, retention was excellent in both groups; selective enamel etching reduced marginal staining at enamel and minor marginal defects compared with no selective etching.
Fuentes_2023	Spain	≥18y; ≥20 teeth in occlusion; ≥2 NCCLs requiring restoration; exclusions: poor oral hygiene, uncontrolled caries/periodontal disease, xerostomia, severe bruxism, bleaching/orthodontic treatment, non-vital/fractured/supporting teeth for prostheses, periodontal surgery in last 3 months; serious medical problems, resin allergy, pregnant/breastfeeding.	Randomized clinical trial with within-subject allocation; 39 patients; 134 restorations; 4 arms: 3-ER (n=34), 2-ER (n=34), 2-SE (n=31), 1-SE (n=35); all restored with Filtek Supreme XTE.	Baseline; 18, 36, 60 months	Additional hydrophobic bonding resin did not improve clinical performance of Scotchbond Universal after 5 years; higher retention with etch-and-rinse strategies.
Gallo_2005	USA	NCCLs present; absence of severe medical complications, rampant caries, xerostomia, chronic periodontitis; restored teeth in occlusion with ≥1 proximal contact.	Clinical trial (split-mouth/within-subject); 30 patients; 90 restorations; 3 groups: F2000SE (n≈29), F2000SB (n≈31), SiluxSB (n≈31).	Baseline (1 week), 6 months, 12, 24, 36 months	Silux Plus and F2000 performed satisfactorily over 3 years; Silux retained surface finish better; F2000 performed successfully with either self-etch primer/adhesive or total-etch adhesive.

Haak_2019	Germany	Adults with NCCLs in permanent teeth; vital teeth; restorations placed under standardized clinical protocol; exclusions not reported here in detail.	Double-blind randomized clinical trial; 55 patients; 165 NCCL restorations; 3 parallel arms (n=55 restorations/arm): SBU-SE, SBU-SEE, Optibond FL ER; all restored with Filtek Supreme XTE.	Baseline (≈14 days); 6 months; 12 months	Scotchbond Universal showed better clinical performance vs Optibond FL at 1 year; selective-enamel-etch with SBU recommended; OCT may predict impending retention loss.
Haak_2022	Germany	Adults (≥18y) with 4 NCCLs in permanent teeth (incisors/canines/premolars) per patient; 22 patients (88 lesions) restored with direct composite restorations.	Randomized split-mouth clinical trial; 22 patients; 88 NCCLs; 4 arms (n=22 restorations/arm): SBU-SE, SBU-SEE, SBU-ER, OFL-ER; composite Filtek Supreme XTE in all groups; FDI criteria; QMA subset (11 patients).	BL (14 days), 6, 12, 24, 36 months	Selective-enamel-etch with Scotchbond Universal (SBU-SEE) showed the most favorable performance; OptiBond FL (OFL) exhibited higher cumulative failure and more marginal gaps; QMA detected group differences earlier than clinical evaluation.
Haefer_2015	Germany	Adults (18–66y); ≥2 NCCLs requiring restorations; positive pulp status (CO_2-snow) on trial tooth; physiological occlusion with natural dentition; exclusions: <20 teeth, heavy bruxism, allergies to ingredients, abutment to fixed/removable prosthesis	Randomized prospective clinical trial; 40 patients; 110 restorations; arms: 1-SE n=40, 2-ER n=40, 4-ER control n=30	Baseline (14 days); 6, 12, 24, 36 months	One-step self-etch Futurabond M/Amaris was clinically comparable to 4-step ER control and outperformed 2-step ER; recommended for NCCLs.
Jassal_2018	India	>18y; acceptable oral hygiene; ≥20 teeth in occlusion; ≥3 NCCLs in different teeth; lesions noncarious, nonretentive, depth≈1 mm; ≤50% enamel at cavosurface margin; enamel+dentin; vital teeth; no mobility. Exclusions: extremely poor hygiene, severe periodontal disease, rampant caries, heavy bruxism.	Randomized double-blind clinical trial; 56 patients; 294 restorations total; 3 groups (98 restorations/group): A-1SEA (G-Bond active + Solare-X), P-1SEA (G-Bond passive + Solare-X), RMGIC (GC II LC).	Baseline; 6, 12, 18 months	Mild one-step self-etch adhesive + composite performed similarly to RMGIC for retention; agitation may improve performance but without statistically significant confirmation; RMGIC showed more progressive marginal staining over time.
Kemalogu_2020	Turkey	≥18y; good general health; available for follow-ups; ≥4 NCCLs in vital canines/premolars with North Carolina Dentin Sclerosis Scale score 3–	Randomized clinical trial; 25 patients; 100 restorations; 4 arms (n=25	Baseline; 6, 12, 24 months	After 24 months, etch-and-rinse and self-etch modes showed similar overall clinical performance; marginal

		4; exclusions: compromised medical history, severe/active periodontal disease, caries, heavy bruxism, traumatic occlusion.	restorations/arm): SEE+Charisma; ER+Charisma; SEE+G-aenial; ER+G-aenial; Single Bond Universal used in SEE vs ER mode.		discoloration was higher with self-etch; selective-etch may be favorable.
Kubo_2006	Japan	Human patients with NCCLs restored clinically; consent obtained; 8 patients (45–78y) with 72 NCCLs.	Controlled clinical trial with within-patient allocation by jaw quadrants; 72 restorations total: LB n=37, SB n=35; one operator; enamel bevel placed (except 3 lesions/group); no retention grooves; all restored with Clearfil AP-X.	Baseline and annual recalls up to 5 years (patients attended regular recalls)	Both adhesive systems showed excellent 5-year clinical performance with no significant between-group differences; retention remained 100% in evaluated restorations; marginal discoloration was the main minor finding.
Lawson_2015	USA	Patients: ≥19y; good general health; available for follow-up; ≥28 teeth. Exclusions: rampant uncontrolled caries; advanced untreated periodontal disease; >2 packs/day smoking or equivalent; systemic/local disorders contraindicating procedures; xerostomia; severe bruxing/clenching/TMD; pregnancy at screening/restoration; known sensitivity to acrylates. Restorations: ≥3 NCCLs, depth ≥1.5 mm, extending to dentin. Tooth exclusions: periapical/pulpal pathology, non-vital/RCT, previous pulp cap, tooth hypersensitivity, near exposures, caries/previous restoration.	Single-center, randomized, comparator-controlled, parallel RCT; 37 adults; 126 NCCLs; 3 arms (n=42 restorations/arm): (1) Scotchbond Universal total-etch, (2) Scotchbond Universal self-etch, (3) Scotchbond Multi-Purpose total-etch; bevel 0.5 mm; rubber dam; Filtek Supreme Ultra placed in ≤2 mm increments; evaluators blinded; modified USPHS/Cvar & Ryge criteria.	Baseline (1 week), 6, 12, 24 months	Scotchbond Universal (total-etch and self-etch) performed similar to or better than Scotchbond Multi-Purpose at 24 months; retention highest for Universal total-etch (100%).

Loguercio_2003	Brazil	Healthy; ≥20 teeth; no poor hygiene or bruxism; ≥1 pair of equal-sized NCCLs under occlusion; expulsive/no undercuts; ≤50% enamel at cavosurface margin; cervical wall in cementum; exclude dentin sclerosis not class 2–3 and lesions with hypersensitivity.	Double-blind randomized clinical trial; 12 patients; 32 restorations total (16 Vitremer, 16 Dyract) placed by 2 calibrated operators; USPHS (Barnes et al adapted) by 2 blinded examiners at baseline and 5 years.	Baseline; 5 years (60 months)	Vitremer showed better marginal adaptation and less marginal discoloration than Dyract; retention numerically higher (not significant); both materials acceptable options at 5 years.
Loguercio_2007	Brazil	Healthy; ≥20 teeth; oral hygiene instructions; exclude poor hygiene, severe/chronic periodontitis, heavy bruxism; ≥2 pairs of similarly sized NCCLs under occlusion; lesions nonretentive/no undercuts; ≤50% enamel at cavosurface margin; cervical wall in cementum; exclude sclerotic dentin degree 1 or 4 and hypersensitive lesions.	Paired-tooth randomized controlled clinical trial; 25 patients; 78 restorations total (39 ER, 39 SE); rubber dam isolation; no bevel/retention; incremental placement of Filtek A110; USPHS evaluations by 2 blinded examiners.	Baseline; 6, 12, 18, 36 months	ER and SE showed similar retention after 36 months; SE exhibited faster/progressive enamel marginal degradation and more marginal discoloration.
Loguercio_2008	Brazil	Healthy; ≥20 teeth; oral hygiene instruction; exclude extremely poor hygiene, severe/chronic periodontitis, heavy bruxism; ≥2 pairs of similarly sized NCCLs without undercuts; dentin sclerosis categories 2–3; all teeth with occlusal contact; ≤50% enamel at cavosurface margin; exclude lesions with hypersensitivity.	Randomized controlled prospective trial; 29 patients; 116 restorations (29/group): OS2 (phosphoric acid + One-Step Plus, 2 coats), OS4 (phosphoric acid + One-Step Plus, 4 coats), TY2 (Tyrian SPE + One-Step Plus, 2 coats), TY4 (Tyrian SPE + One-Step Plus, 4 coats); all restored with Micronew.	Baseline; 6, 12, 18 months	Multiple adhesive coats improved retention; TY2 had the lowest retention; OS4 was the most stable; none met ADA retention guidelines at 18 months.
Loguercio_2015	Brazil	≥18y; good general health; acceptable oral hygiene; ≥20 teeth in occlusion; ≥4 NCCLs in different teeth needing restoration; lesions non-carious, non-retentive, depth >1 mm,	Randomized double-blind clinical trial; 39 patients; 200 restorations; 4 arms (n=50 restorations/arm):	Baseline; 6, 18, 36 months	Clinical behavior did not depend on bonding strategy up to 36 months; slight degradation signs when used in

		enamel+dentin, vital teeth, no mobility; ≤50% enamel at cavosurface margin; exclusions: extremely poor hygiene, severe/chronic periodontitis, heavy bruxism.	ERm, ERd, Set, SE; all restored with Filtek Supreme Ultra.		self-etch mode; FDI more sensitive than USPHS.
Lopes_2016	Brazil	≥18y; good general health; acceptable oral hygiene; ≥20 teeth in occlusion; ≥4 NCCLs in different teeth; lesions noncarious, nonretentive, depth >1 mm, enamel+dentin, vital teeth, no mobility; ≤50% enamel at cavosurface margin; exclusions: extremely poor hygiene, severe/chronic periodontitis, heavy bruxism.	Double-blind randomized split-mouth RCT; 31 patients; 124 restorations; 4 arms (n=31 restorations/arm): ER-D, ER-M, SE-et, SET; EvoluX placed incrementally.	Baseline (1 week); 6 months	At 6 months, clinical behavior depended on bonding strategy; Xeno Select did not meet ADA full approval criteria when used in self-etch mode.
Manarte-Monteiro_2022	Portugal	Need to perform ≥1 and ≤6 NCCLs (>1.5 mm; enamel+dentin) in vital premolars/molars; include only very good/good oral hygiene (OHI-S 0.0–3.0); exclude: no consent, <20 teeth in occlusion, orthodontic treatment, pregnancy, severe/chronic periodontitis or periodontal surgery in prior 3 months, severe bruxism, compromised medical/psychiatric/pharmacotherapy history, allergies/idiosyncratic reactions, teeth supporting fixed/removable prosthesis, poor hygiene (OHI-S 3.1–6.0), high caries/pulp injury risk (ICDAS II ≠00 with active lesions).	Randomized clinical trial; 35 participants; 210 restorations; 6 arms (n=35 restorations/arm): FBDC-SE, FBDC-SE-EE, FBU-ER, FBU-SE, ADU-ER, ADU-SE; all restored with Admira Fusion.	Baseline; 12 and 24 months (2-year)	At 2 years, retention loss occurred in all groups without significant between-group differences; overall, FBDC and FBU showed worse performance than ADU in SE mode; age and dentin sclerosis influenced performance.
Merle_2022	Germany	≥18y; vital teeth with natural antagonist in occlusion; 3–4 NCCLs in incisors/canines/premolars; exclusions: inability to control contamination, lesion contacting existing restorations, periodontal inflammation/PPD>4 mm/mobility>1, <20 teeth, removable dentures, bruxism/habits, alcohol/drug dependence, pregnancy, allergies.	Four-arm parallel-group RCT; 50 patients; 179 NCCLs/restorations randomized by lesion; restored with Venus Diamond Flow; groups: iBU-SE (n=50 restorations), iBU-SEE (n=29), iBU-ER (n=50), OFL (n=50).	Baseline (14 days), 6 months, 12 months	iBU showed lower cumulative failures than OFL at 12 months; recommended mode: selective enamel etching (SEE).

Moosavi_2013	Iran	Patients 20–50y; ≥3 noncarious, nonsclerotic NCCLs; healthy periodontium; normal occlusion; ≤50% enamel at cavosurface margin; buccal lesions only; no prior restorations/caries on other surfaces; cavity depth ≤2 mm; isolation possible; exclusions: severe medical complications, poor oral hygiene, extreme caries susceptibility, heavy bruxism.	30 patients; 90 NCCLs restored with Herculite XRV; 3 arms: OptiBond FL (3-step E&R), OptiBond Solo Plus (2-step E&R), OptiBond All-In-One (1-step SE); one operator; randomized allocation; no bevel/grooves; cotton roll + retraction cord isolation; USPHS evaluation by 2 blinded examiners.	Baseline (1 week); 6, 12, 18 months	Three adhesives showed acceptable 18-month clinical effectiveness in NCCLs; one-step self-etch may lead to marginal discoloration; overall performance comparable.
Naupari-Villasante_2025	Brazil	≥18y; good general health (ASA I–II); acceptable oral hygiene (OHI-S <3); minimum of four comparable non-retentive NCCLs (>1 mm depth) on four different teeth involving enamel+dentin in vital teeth; cavo-surface margin ≤50% enamel; exclusions: severe/chronic periodontitis; orthodontic appliances; >10 teeth with occlusal wear facets; allergies to materials; pregnant/breastfeeding; anti-inflammatory/analgesic/psychotropic drug use.	Double-blind split-mouth two-center RCT; 50 participants; 200 restorations; 4 arms (n=50 restorations/arm): SEE, SET, ERDry, ERWet; all restored with Admira Fusion.	Baseline; 6 months; 1, 3, 5, 7.5 years (0, 6, 12, 36, 60, 90 months)	After 7.5 years, the clinical performance of Futurabond U in NCCLs was not significantly influenced by adhesive strategy; retention and secondary outcomes were comparable across groups.
ÑaupariVillasante_2023	Brazil	>18y; good general health; acceptable oral hygiene (OHI-S); ≥20 teeth in occlusion; ≥4 comparable NCCLs in 4 different teeth requiring restoration; lesions non-retentive, depth >1 mm, enamel+dentin, vital teeth; cavosurface margin ≤50% enamel; exclusions: OHI-S>3, severe/chronic periodontitis, orthodontic devices, >10 teeth with occlusal facets, allergy to materials, pregnant/breastfeeding, anti-inflammatories/analgesics/psychotropic drugs use.	Multicenter, double-blind, split-mouth RCT; 50 participants; 200 restorations; 4 arms (n=50 restorations/arm): SE, SET+SE, ERD, ERW; all restored with Admira Fusion; evaluated with FDI and modified USPHS.	Baseline; 12, 36, 60 months (1, 3, 5 years)	After 5 years, clinical performance (including retention) was satisfactory and not influenced by bonding strategy.

Onal_2005	Turkey	Age 27–64y; ≥3 cervical lesions; vital pulps required; exclusions: severe medical complications, rampant caries or severe chronic periodontitis, severe active bruxism, xerostomia, pulpitis, apical periodontitis.	Double-blind randomized clinical trial; 30 patients; 130 restorations: Vitremer (n=24), F2000 Compomer (n=38), Dyract AP (n=46), Valux Plus (n=22); no enamel bevel; no mechanical retention; evaluated by 2 calibrated examiners.	Baseline (≤2 weeks); 1 year; 2 years	Vitremer had 100% retention at 2 years and was significantly higher than other materials; Valux Plus showed the most favorable performance in other criteria; no secondary caries detected. :contentReference[oaicite:0]{index=0}
Oz_2019	Turkey	≥18y; able to attend recalls; ≥7 NCCLs; antagonist and adjacent teeth present; lesion depth 1–3 mm; exclusions: poor gingival health, uncontrolled/rampant caries, bruxism, removable partial denture, xerostomia; severe hypersensitivity excluded (pain score 7–10).	Randomized controlled clinical trial with within-patient allocation (each patient received all 7 groups); 20 patients; 155 NCCL restorations; 7 arms with Tetric N-Ceram composite: GSE n=21, GSL n=20, GER n=22, ASE n=20, ASL n=21, AER n=22, SBE n=29.	Baseline (1 week); 6, 12, 24 months	Universal adhesives (GLUMA Universal, All-Bond Universal) performed better in etch-and-rinse and selective-etch modes than in self-etch mode regarding retention; other USPHS criteria showed no significant between-group differences over 24 months, except retention.
Oz_2022	Turkey	≥18y; ≥20 teeth in occlusion; ≥5 NCCLs requiring restoration in different teeth, similar size/depth 1–3 mm. Exclusions: uncontrolled caries; xerostomia; medical problems preventing review visits; poor gingival health; bruxism; removable partial dentures; severe hypersensitivity (cold test).	Randomized controlled clinical trial (within-patient allocation of ≥5 NCCLs to 5 protocols); 34 patients (mean age 49y; 12 males/22 females); 234 NCCLs. Baseline restorations/arm: CUQ-SE n=46; CUQ-SLE n=47; CUQ-ER n=47; CSEB n=47; TBU-ER n=47. All restored with Tetric N-Ceram.	Baseline; 6, 12, 24 months	The 24-month survival of Clearfil Universal Bond Quick used in self-etch mode was not acceptable; CUQ in selective enamel-etch and etch-and-rinse modes showed clinically acceptable performance per USPHS criteria.
Ozel_2010	Turkey	Patients with at least two pairs of NCCLs; Class I occlusion without absence of ≥2 units	Paired clinical trial; 22 patients; 104 NCCL	Baseline; 12 months	At 1 year, both techniques showed excellent clinical performance;

		in molar region; no removable prostheses; good oral hygiene and periodontal health; NCCLs with advanced dentine sclerosis category 3–4 (North Carolina Dentine Sclerosis Scale); lesion depth >2 mm; incisal/occlusal margin in enamel and gingival margin in dentine/cementum.	restorations; 2 arms (n=52 restorations/arm): AdheSE non-etch (SEE on enamel+dentine) vs AdheSE etch (selective enamel etch with 37% phosphoric acid 30 s + AdheSE on enamel+dentine); restorations placed by one operator; Point 4 used in all restorations.		additional enamel etching did not improve overall short-term clinical quality.
Pappa_2024	Greece	≥19y; good general health; available for follow-up; ≥20 teeth; exclusions: rampant uncontrolled caries/high caries activity; advanced untreated periodontal disease/periodontal treatment; >2 cigarette packs/day or chewing tobacco equivalent; systemic/local disorders contraindicating dental procedures; xerostomia; severe bruxism/clenching/TMD; orthodontic treatment. NCCL-level: ≥3 NCCLs on incisors/canines/premolars; buccal cervical third; cervical wall on dentin; not extending to adjacent surfaces; ≤5 mm length, ≤3 mm height, ≤1.5 mm depth.	Split-mouth, single-center randomized controlled clinical trial; 32 patients; 96 NCCLs; 3 arms (one lesion per arm per patient): Method A ER/total-etch (37% phosphoric acid: enamel 30 s, dentin 15 s) + ExciTE F; Method B self-etch AdheSE One F without prior etching; Method C selective enamel etch (37% phosphoric acid 30 s) + AdheSE One F; all restored with Tetric EvoCeram; sclerotic dentin superficially ground and enamel beveled before bonding.	Baseline; 6, 12, 18, 24 months	All three adhesive strategies yielded clinically acceptable restorations at 2 years with no significant differences in retention or marginal adaptation; total-etch showed better marginal staining performance.
Pena_2016	Brazil	Patients needed ≥4 NCCLs; excluded if compromised medical history, severe/chronic periodontitis, extreme caries sensitivity, heavy	Randomized controlled prospective clinical trial; 25 patients; 112 NCCLs; 4 arms (n=28	Baseline; 3, 6, 12, 18, 24 months	Overall clinical effectiveness acceptable at 24 months; selective enamel etching did not affect overall

		bruxism, orthodontic treatment, poor oral hygiene, smokers.	restorations/arm): CSE-NE (self-etch), CSE-E (selective enamel etch + self-etch), XV-NE (self-etch), XV-E (selective enamel etch + self-etch). All restored with Esthet.X HD.		performance; marginal staining increased in CSE-NE and XV-E.
Perdigao_2005	USA	Adults (22–55y); ≥24 teeth; NCCLs without undercuts; ≤50% cavosurface angle in enamel; ≤4 restorations/patient; periodontal health considered normal. Exclusions: active caries lesion; tooth restored in prior 3 months; non-vital tooth; fractured/cracked tooth; abutment teeth for fixed/removable prostheses; teeth/supporting tissues with painful pathology; bruxism; xerostomia or periodontal disease; orthodontic treatment in preceding 3 months; allergies/idiosyncratic response to materials; inability to return for recalls; pregnant/breastfeeding; periodontal surgery in prior 3 months.	Randomized clinical trial; 34 patients; 120 NCCL restorations; 4 arms (n=30 restorations/arm): Group1 CSEB no bevel/no etch; Group2 bevel + CSEB; Group3 enamel etch (35% phosphoric acid 15 s) + CSEB; Group4 bevel + enamel etch + CSEB; all restored with Filtek A110.	Baseline; 6, 18 months	Beveling and/or enamel etching did not affect 18-month clinical performance of the self-etch adhesive; overall marginal discoloration and marginal adaptation worsened at 18 months vs baseline, while sensitivity to air decreased from baseline to 18 months.
Perdigao_2014	Brazil	≥18y; good general health; acceptable oral hygiene; ≥20 teeth in occlusion; ≥4 NCCLs in different teeth; lesions noncarious, nonretentive, depth >1 mm, enamel+dentin, vital teeth, no mobility; ≤50% enamel at cavosurface margin; exclusions: extremely poor hygiene, severe/chronic periodontitis, heavy bruxism.	Double-blind, randomized, split-mouth clinical trial; 39 patients; 200 NCCL restorations; 4 arms (n=50 restorations/arm): ERm, ERd, Set, SE; all restored with Filtek Supreme Ultra.	Baseline; 6, 18 months	At 18 months, Scotchbond Universal showed reliable clinical performance in NCCLs and met ADA acceptance criteria; outcomes were not significantly influenced by bonding strategy.
Perdigao_2019	Spain	≥18y; ≥20 teeth in occlusion; ≥2 NCCLs requiring restoration; exclusions: poor oral hygiene; uncontrolled caries/periodontal disease; xerostomia; serious medical problems; severe bruxism; resin allergy; pregnant/breastfeeding; bleaching/orthodontic	Randomized clinical trial; 39 patients; 134 NCCL restorations; 4 strategies with baseline restorations/group: 3-ER n=34, 2-ER n=34, 2-SE	Baseline; 6, 18, 36 months	At 36 months, etch-and-rinse strategies showed superior clinical performance (higher retention); phosphoric acid etching recommended to provide retention in NCCLs.

		treatment; non-vital/fractured teeth; teeth with prostheses; periodontal surgery in previous 3 months.	n=31, 1-SE n=35; same composite used in all groups.		
Perdigao_a 2012	Brazil	Adults needing restoration of NCCLs in permanent teeth; ≥20 teeth; vital teeth with NCCLs without undercuts; exclusions: history of existing tooth sensitivity; bruxism/visible wear facets; inability to return for recalls; fractured or cracked candidate tooth; current desensitizing therapy (including desensitizing dentifrices/OTC products); long-term use of anti-inflammatory/analgesic/psychotropic drugs; pregnancy/breastfeeding; allergy to resin-based materials; orthodontic appliance treatment within previous 3 months; abutment teeth for fixed/removable prostheses; teeth/supporting structures with symptomatic pathology; existing periodontal disease or periodontal surgery within previous 3 months; caries exclusion criterion applied.	Randomized clinical trial; 39 patients; 125 NCCL restorations randomized to 4 adhesive strategies (baseline restorations/group: EB n=34, SE n=30, SB n=32, MP n=29); composite placed incrementally with cotton-roll isolation; evaluated using UNC-modified USPHS criteria.	Baseline; 6 months; 18 months	At 18 months, retention was similar across etch-and-rinse and self-etch strategies; however, enamel marginal deterioration (e.g., marginal adaptation/interfacial staining) was more prevalent for self-etch adhesives.
Perdigao_b 2012	Brazil	Adults (30–79y) with NCCLs (Class V) in permanent teeth; vital teeth; ≥20 teeth; NCCL without undercuts; exclusions: caries, chronic sensitivity, bruxism/wear facets, inability to return, cracked/fractured tooth, current desensitizing therapy, chronic anti-inflammatory/analgesic/psychotropic use, pregnancy/breastfeeding, allergy to resin materials, recent orthodontic treatment, abutment teeth, symptomatic pathology, periodontal disease/surgery.	Randomized clinical trial; 33 patients; 92 NCCLs; 3 arms with baseline restorations: Ambar+Filtek Supreme Plus (n=31), Fuji II LC (n=31), Ketac Nano (n=30).	Baseline; 6 months; 12 months (1 year)	One-year retention similar among groups; enamel marginal deficiencies and color mismatch more prevalent for Ketac Nano; surface texture of Fuji II LC deteriorated quickly.
Peumans_2005	Belgium	Non-hospitalized adults needing treatment of non-carious Class V cervical lesions; cervical margin in dentin and incisal margin in enamel; no labial restoration; small interproximal restoration allowed; exclusions: complex	Paired-tooth randomized controlled prospective clinical trial; 29 patients; 100 NCCLs; 2 arms (n=50 restorations/arm): C-SE	Baseline; 6 months; 12 months; 24 months; 36 months	Clearfil SE showed excellent 3-year clinical performance; prior selective enamel etching was not critical, although non-etch showed more small

		medical history, severe/chronic periodontitis, extreme caries sensitivity, heavy bruxism (>50% tooth structure lost by attrition).	non-etch vs C-SE etch (selective enamel phosphoric acid) using same adhesive and same composite.		enamel marginal defects and related superficial discoloration.
Peumans_2010	Belgium	Non-carious Class-V (cervical) lesions in 29 patients (mean age 58y); detailed selection criteria reported in the 3-year report (Peumans et al., 2005) and not fully reiterated here.	Randomized, double-blind, split-mouth RCT; 29 patients; 100 restorations; 2 arms (n=50 restorations/arm): C-SE non-etch vs C-SE etch; all restored with Clearfil AP-X.	6 months; 1, 2, 3, 5, and 8 years	At 8 years, Clearfil SE showed excellent clinical effectiveness; selective enamel etching provided only minor improvements in enamel marginal integrity/discoloration without affecting overall success.
Peumans_2015	Belgium	Non-hospitalized patients needing cervical restorations; exclusions: compromised medical history, severe/chronic periodontitis, extreme caries sensitivity, heavy bruxism.	Paired-tooth randomized controlled clinical trial; 29 patients; 100 NCCLs; 2 arms (n=50 restorations/arm): CSE-NE vs CSE-E; rubber dam; dentin walls roughened; 1–2 mm enamel bevel; Clearfil AP-X used in both arms.	Baseline; 6, 12, 24, 36, 60, 96, 156 months	After 13 years, Clearfil SE Bond showed excellent clinical performance; selective enamel etching had only minor positive effects on marginal integrity/discoloration.
Peumans_2018	Belgium	52 patients with at least two non-carious cervical lesions (NCCLs) were included.	Randomized clinical trial; 52 patients; 267 NCCLs; 2 arms: GB (n=133 restorations) vs OFL (n=134 restorations); all lesions restored with Gradia Direct after standardized cavity cleaning, superficial dentin roughening, and 1–2 mm coronal enamel bevel; adhesives applied	Baseline; 6, 12, 24, 36, 60, 108 months	After 9 years, the HEMA-free one-step self-etch adhesive performed similarly to the 3-step etch-and-rinse adhesive for overall clinical success and retention; marginal deterioration at the incisal enamel side was more pronounced for the one-step self-etch adhesive.

			per manufacturer instructions.		
Peumans_2021	Belgium	Human patients with NCCLs in permanent teeth; lesion margins with incisal margin in enamel and cervical margin in dentin; exclusions included complex medical history, severe/chronic periodontitis, high caries risk, and severe bruxism.	Randomized, double-blind, split-mouth clinical trial; 50 patients; 239 NCCL restorations; 2 arms (OptiBond XTR vs OptiBond FL); restorations placed with Herculite XRV Ultra.	Baseline (1 week); 1 year (12 mo); 2 years (24 mo); 6 years (72 mo)	Within the limitations, the 2-step self-etch adhesive (OptiBond XTR) showed good and comparable clinical performance to the 3-step etch-and-rinse control (OptiBond FL) over 6 years.
Peumans_2023	Belgium	Non-hospitalized patients needing restorative treatment of NCCLs (reasons: sensitivity, prevention of further tooth wear, and/or esthetic complaints); exclusions: complex medical history, severe/chronic periodontitis, high caries risk, severe bruxism.	Paired-tooth RCT (split-mouth); 51 patients; 251 restorations; 2 arms: CUBQ-ER (n=122 restorations) vs CUBQ-SEE (n=129 restorations); all restored with Clearfil Majesty ES-2.	Baseline; 1 year; 3 years	After 3 years, Clearfil Universal Bond Quick performed similarly in ER and self-etch mode with prior selective enamel etching; no significant differences between groups for evaluated parameters.
Ranjitha_2020	India	Adults 35–55y; minimum ≥2 NCCLs; posterior permanent teeth; healthy periodontium; in occlusion with opposing teeth; normal occlusal relationship; buccal cervical lesions with <50% enamel at cavosurface margin; ethical approval and informed consent.	Prospective randomized double-blind parallel-group clinical trial; 100 posterior NCCL restorations; 2 arms: SET (n=50 restorations) vs self-etch (n=50 restorations); adhesive: G-Premio Bond; restorative: Genial Flow.	1 week (baseline); 6 months; 12 months	At 12 months, overall clinical performance was comparable between selective-etch and self-etch strategies with the same universal adhesive; failures were due to debonding (total 3).
Ruschel_2018	Brazil	Adults with ≥1 notch-shaped NCCL; ≥20 teeth; acceptable oral hygiene; exclusions: poor hygiene, uncontrolled periodontal disease, xerostomia, allergy to resin materials, medically compromised, pregnant/breast-feeding; teeth excluded if saucer-shaped, nonvital, not in occlusion, previously restored.	Randomized controlled clinical trial; 63 subjects; 203 NCCLs; 4 groups: SU_E&R (n=52), SU_SE (n=50), PBE_E&R (n=50), PBE_SE (n=51); operator placed all restorations;	Baseline; 18 months	Scotchbond Universal and Prime & Bond Elect showed acceptable clinical performance at 18 months; SU self-etch showed relatively high marginal discoloration compared with other groups.

			evaluators calibrated; criteria modified USPHS.		
Ruschel_2019	Brazil	Adults needing restoration of ≥1 notch-shaped NCCL; ≥20 teeth; acceptable oral hygiene; exclusions: poor hygiene, uncontrolled periodontal disease, xerostomia, allergy to resin-based materials, medically compromised, pregnant/breast-feeding; tooth exclusions: saucer-shaped lesions, nonvital, not in occlusion, previously restored.	Randomized controlled clinical trial; 63 subjects; 203 NCCLs; 4 arms: SU_ER (n=52 restorations), SU_SE (n=50), PBE_ER (n=50), PBE_SE (n=51); restorative material Kalore for all groups; outcomes assessed with modified USPHS.	Baseline (1 week); 18 months; 36 months	At 36 months, SU (both modes) and PBE in ER showed 100% retention among recalled restorations; PBE in SE showed lower retention; marginal degradation occurred in >20% of restorations; acid effect appeared material-dependent.
Ruschel_2023	Brazil	Adults needing restoration of ≥1 notch-shaped NCCL; all lesions with cavosurface margins including enamel and dentin/cementum; subject exclusions: <20 teeth, poor oral hygiene, uncontrolled periodontal disease, xerostomia, allergy to resin-based materials, medically compromised, pregnant/breast-feeding; tooth exclusions: saucer-shaped lesions, nonvital, not in occlusion, previously restored.	Randomized controlled clinical trial; 63 patients; 203 NCCLs; 4 arms: SU_ER, SU_SE, PBE_ER, PBE_SE; restorations placed with Kalore; modified USPHS evaluation (Alfa vs Bravo+Charlie).	Baseline; 18, 36, 60 months	SU and PBE showed acceptable 5-year retention; few failures overall; phosphoric-acid etching improved PBE performance for marginal discoloration. Marginal degradation (discoloration/adaptation) may occur within 5 years; PBE performed better for marginal discoloration when used with phosphoric-acid etching.
Santiago_2010	Brazil	Age 18–50y; good oral hygiene; low decay index; no periodontal disease or deleterious habits; no wear facets; ≥2 NCCLs; all lesions ≥1 mm deep; permanent teeth.	Controlled clinical trial; 30 patients; 70 NCCLs; 2 arms: Excite/Tetric Ceram (n=35 restorations) vs Vitremer RMGIC (n=35); single operator; rubber dam; no bevel/prep/retention; double-blind evaluation; modified USPHS.	Baseline; 6, 12, 24 months	After 2 years, one-bottle ER adhesive + hybrid composite showed inferior retention versus RMGIC; other criteria similar between materials.
Schwendicke_2021	Germany	50–70y; sclerotic NCCL requiring restoration; sclerosis degree 3–4; lesions accessible (no	Cluster-parallel RCT; 88 patients; 175 sNCCLs;	Baseline (1 week); 18 months; 36 months	Survival not significantly different between GH and RC; GH significantly

		proximal/subgingival extension); cervical margin in dentin and coronal margin in enamel; no inflamed adjacent gingiva; no material allergy; no severe systemic disorders/disabilities.	randomized by patient to GH (43 patients; 83 restorations) vs RC (45; 92); no mechanical prep; cotton roll isolation; FDI evaluation.	(mean 36; range 31–55 due to COVID-19)	less costly; RC superior for surface luster.
Tian_2014	China	50 patients; each with ≥2 wedge-shaped NCCLs; lesions depth and width >1 mm; normal dentition/occlusion; no obvious gingival inflammation; no reported bruxism/clenching; medically fit; ethics approval and informed consent.	Single-center split-mouth RCT; 50 patients; 100 restorations; 2 arms (n=50 restorations/arm): Group α ER (Tetric N-bond + 37% phosphoric acid), Group β self-etch (Tetric N-bond self-etch); restored with Tetric N-Ceram.	Baseline (1 week); 6 months; 18 months	Within 18 months, two-step etch-and-rinse and one-step self-etch adhesives showed similar clinical performance; longer follow-up warranted.
Tuncer_2013	Turkey	Patients with NCCLs; exclusions: extremely poor oral hygiene; history of bruxism or xerostomia; severe medical complications; severe chronic periodontitis. Lesions wedge-/saucer-shaped; cervical margin in dentin and incisal margin in enamel; depth ≤2 mm; teeth in occlusion and proximal contact.	Double-blind split-mouth RCT; 24 patients; 123 NCCLs; 2 arms: Solobond M ER (n=62 restorations) vs Futurabond NR all-in-one self-etch (n=61); all restored with Grandio.	Baseline; 6, 12, 24 months	Etch-and-rinse showed higher retention than all-in-one self-etch at 24 months; no significant differences for colour match, marginal staining, or marginal adaptation; no secondary caries or anatomic form loss.
vanDijken_2013	Sweden	Human adults with non-carious cervical lesions (permanent teeth) restored with adhesive resin composite; clinical evaluation using modified USPHS criteria; exclusions not explicitly detailed in the extracted tables; restorative procedures followed ADA clinical guidelines.	Randomized clinical trial; 67 patients; 169 NCCL restorations; 3 arms: G-Bond+Gradia Direct (n=67 restorations), cfm+els (n=51), XP Bond+els (n=51).	Baseline; 6, 12, 18, 24 months; then annually to 5 years	At 5 years, the HEMA-free 1-step self-etch adhesive (G-Bond) and the 3-step etch-and-rinse system (cfm) showed significantly higher retention than XP Bond; loss of retention was the only failure mode; no postoperative sensitivity reported.
VanLanduyt_2011	Belgium|Turkey	Patients with non-carious cervical lesions (NCCLs) in permanent teeth; all lesions per enrolled patient meeting study inclusion criteria (detailed in the 1-year report) were restored with direct composite restorations.	Randomized controlled clinical trial with multiple NCCLs per patient (within-patient allocation); 52 patients; 267 restorations (G-Bond n=133; Optibond	Baseline; 6, 12, 24, 36 months	After 3 years, the HEMA-free one-step self-etch adhesive showed similar overall clinical success to the 3-step etch-and-rinse control, but exhibited more (mostly small) enamel marginal defects and marginal discolorations;

			FL n=134); all restored with Gradia Direct micro-hybrid composite.		large/sclerotic lesions were more prone to retention loss.
VanMeerbeek_2005	Belgium	Non-hospitalized patients needing cervical restorations; NCCLs in permanent teeth; paired equal teeth; exclusions: compromised medical history, severe/chronic periodontitis, extreme caries sensitivity, heavy bruxism.	Paired-tooth RCT; 29 patients; 100 restorations (50 pairs); 2 arms (n=50 restorations/arm at baseline).	Baseline; 6 months; 1 year; 2 years	No restorations lost up to 2 years; overall clinical success excellent; selective enamel etching reduced small enamel marginal defects.
Vural_2019	Turkey	Patients with NCCLs and chronic bruxism; age 42–72y; ≥2 lesions/patient; all teeth with antagonistic contact; exclusions: intensive medical history, chronic periodontitis, high caries activity, extremely poor oral hygiene, teeth with existing restorations, mobility due to periodontal disease, contact with removable prosthetic construction.	Double-blind split-mouth RCT; 25 patients; 148 NCCLs; 2 arms: GH (n=74 restorations) vs RBC (n=74 restorations).	Baseline (1 week); 6, 12, 24 months	At 24 months, no significant difference in retention; nano-ceramic RBC showed better marginal adaptation; both materials clinically acceptable; no secondary caries or postoperative sensitivity observed.
Yaman_2014	Turkey	Healthy adults; ≥20 teeth; complex medical history/severe or chronic periodontitis/extreme caries/heavy bruxism excluded; ≥6 pairs of equally sized NCCLs under occlusion; lesion depth >1 mm and <2 mm; cervicoincisal height >1.5 mm and <2.5 mm.	Double-blind randomized clinical trial; 24 patients; 144 NCCLs; 3 arms (n=48 restorations/arm): FS/SSA (SE), CXM/CSE (SE), CXM/XPB (ER).	Baseline (1 week); 6, 12, 24, 36 months	At 36 months, no significant intergroup differences; no postoperative sensitivity or secondary caries; overall 3-year performance of silorane vs methacrylate composite systems was comparable.
Zanatta_2019	Brazil	≥18y; good general health; acceptable oral hygiene; ≥20 teeth in occlusion; NCCLs depth ≥1 mm; enamel+dentin margins with ≥50% enamel at cavosurface margin; vital and non-mobile teeth; exclusions: poor oral hygiene, severe bruxism, severe/chronic periodontitis.	Double-blind randomized clinical trial; 34 participants; 152 NCCL restorations; 4 arms at baseline (n=38 restorations/arm): SU/ER, SU/SE, SB/ER, CF/SE; all restored with nanocomposite resin (Filtek Supreme XT).	Baseline; 6, 12, 24 months	Scotchbond Universal performed similarly to conventional etch-and-rinse and self-etch systems across all adhesion strategies over the evaluated follow-up periods.
