# Supplementary material for: Comparison of Adhesive Strategies with Different Etching Approaches on the Clinical Performance of Restorations in Non-Carious Cervical Lesions: A Systematic Review and Network Meta-Analysis
Source: J Funct Biomater. 2026 Mar 25;17(4):160. doi: 10.3390/jfb17040160 (PMC13117247; doi:10.3390/jfb17040160)
Supplement: Supplementary file 1 [file jfb-17-00160-s001.zip › jfb-4192088-Supplementary File S4.pdf]

Supplementary File S4. Summary of risk of bias assessment across included studies using the Cochrane RoB 2 tool.

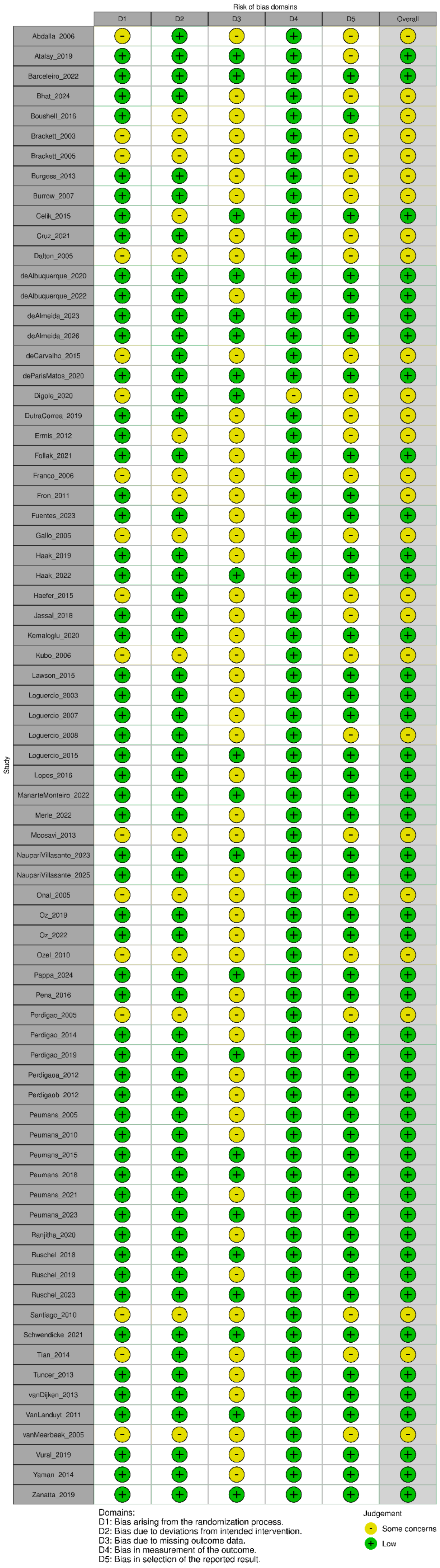

The figure displays the judgment for each study across the five RoB 2 domains: D1, bias arising from the randomization process; D2, bias due to deviations from intended interventions; D3, bias due to missing outcome data; D4, bias in measurement of the outcome; and D5, bias in selection of the reported result. The overall risk of bias judgment for each study is presented in the rightmost column. Green symbols indicate low risk of bias, while yellow symbols indicate some concerns.
