# Supplementary material for: Comparison of Adhesive Strategies with Different Etching Approaches on the Clinical Performance of Restorations in Non-Carious Cervical Lesions: A Systematic Review and Network Meta-Analysis
Source: J Funct Biomater. 2026 Mar 25;17(4):160. doi: 10.3390/jfb17040160 (PMC13117247; doi:10.3390/jfb17040160)
Supplement: Supplementary file 1 [file jfb-17-00160-s001.zip › jfb-4192088-Supplementary File S5.pdf]

Supplementary File S5. Descriptive Reporting of Predefined Outcomes Without Statistically Significant Differences.

This supplementary file reports the results of predefined clinical outcomes assessed in the systematic review that did not demonstrate statistically significant differences between interventions. These outcomes were evaluated as planned and are reported transparently; however, they were not prioritized in the main text due to their limited discriminatory capacity and are therefore presented in detail in the supplementary material.

SP4.1 Outcome Anatomic form

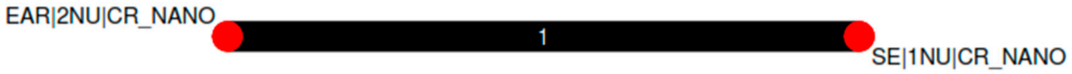

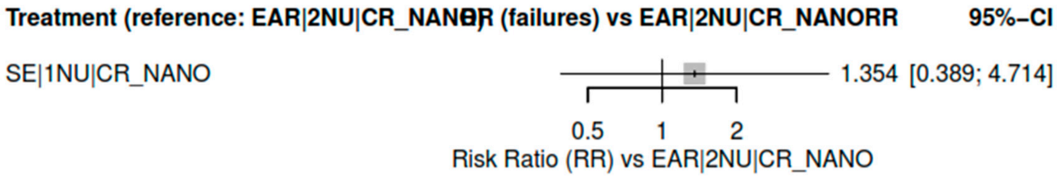

Burgess_2013

SP4.2 Outcome Fracture

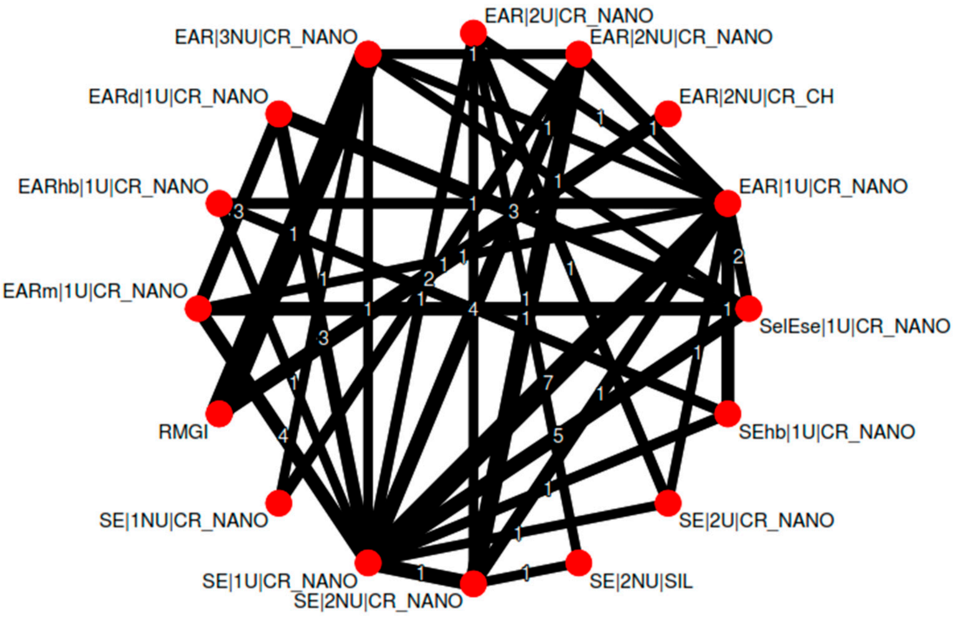

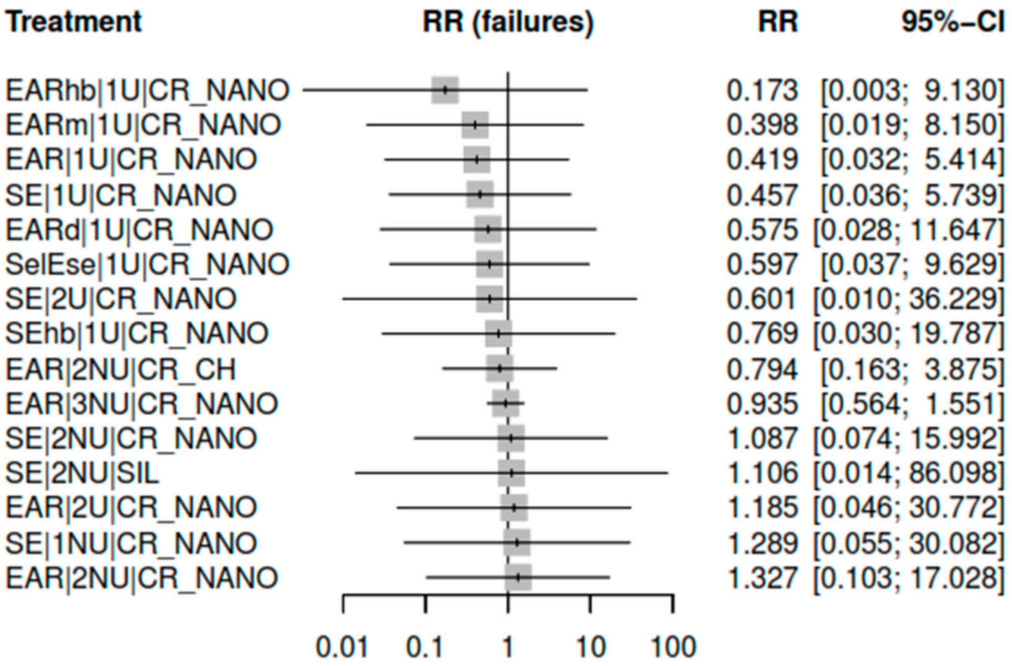

Cruz_2021

Franco_2006

Fuentes_2023

Haak_2022

Haefer_2015

Loguercio_2015

Lopes_2016

Perdigao_2014

Perdigao_2019

Schwendicke_2021

Tuncer_2013

Yaman_2014

Zanatta_2019

deAlmeida_2026

deParisMatos_2020

SP4.3 Outcome Marginal discoloration

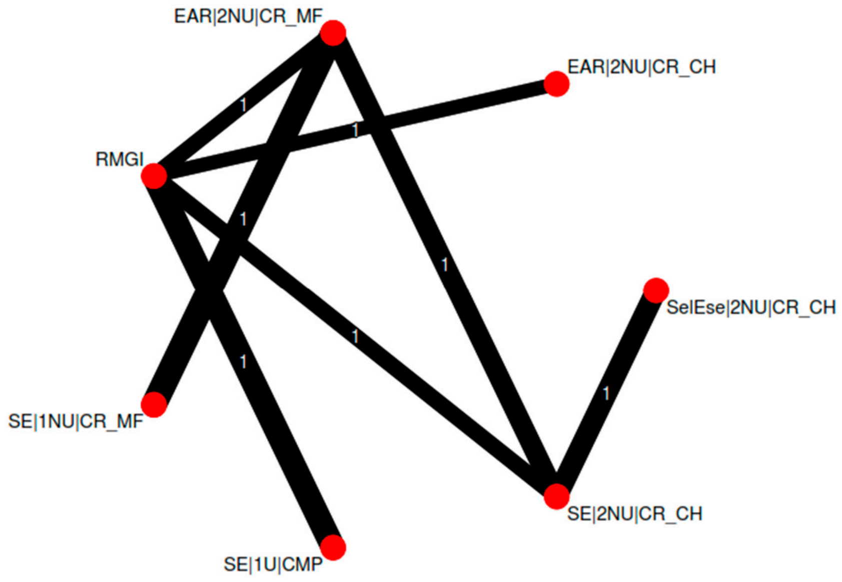

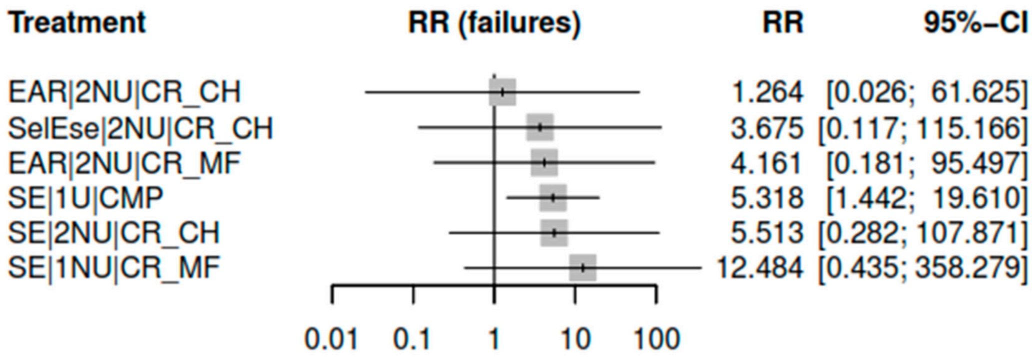

Burrow_2007

Dalton_2005

Loguercio_2003

Santiago_2010

VanMeerbeek_2005

SP4.4 Outcome Postoperative sensitivity

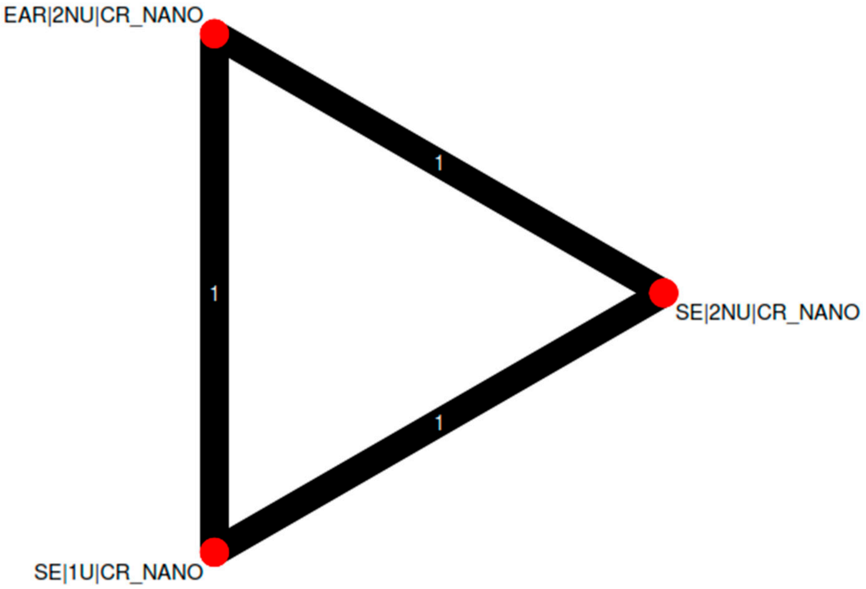

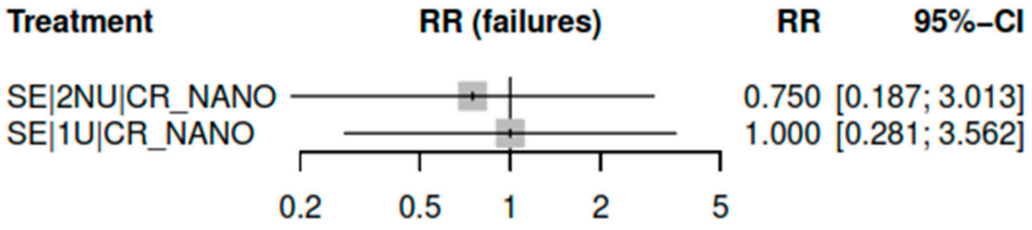

Digole_2020

SP4.5 Outcome Secondary caries

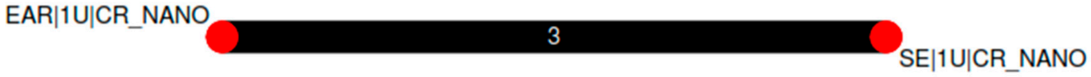

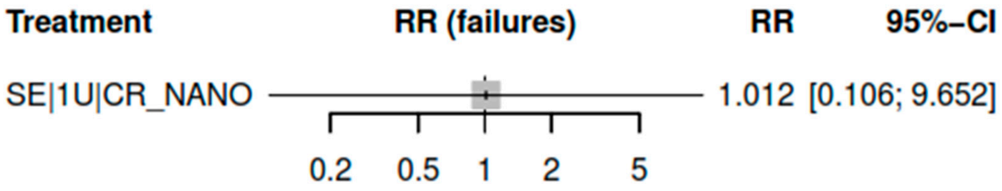

Ruschel_2018

Ruschel_2019

Ruschel_2023

SP4.6 Outcome Surface texture

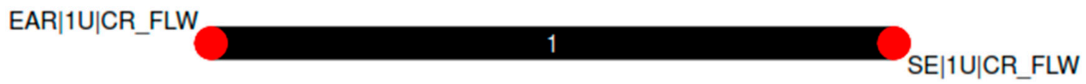

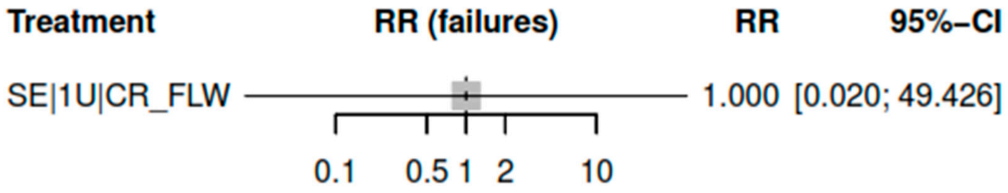

Kemaloglu_2020
