# Supplementary material for: Comparison of Adhesive Strategies with Different Etching Approaches on the Clinical Performance of Restorations in Non-Carious Cervical Lesions: A Systematic Review and Network Meta-Analysis
Source: J Funct Biomater. 2026 Mar 25;17(4):160. doi: 10.3390/jfb17040160 (PMC13117247; doi:10.3390/jfb17040160)
Supplement: Supplementary file 1 [file jfb-17-00160-s001.zip › jfb-4192088-Supplementary File S6.pdf]

Supplementary File S6. League tables of relative treatment effects for marginal adaptation and retention loss (network meta-analysis)

Table SF5.1. League table of relative treatment effects for marginal adaptation.
The table reports pairwise relative risks (RRs) with corresponding 95% confidence intervals derived from a random-effects network meta-analysis. Each cell compares the treatment listed in the row against the treatment listed in the column. Relative risks below 1 indicate a lower risk of marginal adaptation failure and therefore a more favorable clinical performance. Estimates are based on the largest connected component of the evidence network.

<table>
<tr><td>EAR|1U|CR_CH</td><td>1.624 (0.157, 16.842)</td><td>0.730 (0.093, 5.736)</td><td>1.335 (0.128, 13.888)</td><td>0.803 (0.075, 8.569)</td><td>1.946 (0.172, 22.034)</td><td>1.143 (0.154, 8.504)</td><td>1.319 (0.130, 13.401)</td><td>1.924 (0.174, 21.308)</td><td>1.018 (0.045, 23.139)</td><td>1.990 (0.184, 21.482)</td><td>1.500 (0.152, 14.757)</td><td>0.857 (0.113, 6.475)</td><td>0.917 (0.087, 9.614)</td><td>0.363 (0.025, 5.279)</td><td>0.915 (0.127, 6.571)</td><td>1.016 (0.098, 10.495)</td><td>1.182 (0.134, 10.396)</td><td>1.096 (0.105, 11.427)</td><td>1.764 (0.127, 24.476)</td><td>1.060 (0.020, 54.839)</td><td>0.561 (0.025, 12.491)</td><td>6.451 (0.513, 81.118)</td><td>15.789 (0.728, 342.647)</td><td>1.136 (0.106, 12.206)</td><td>1.760 (0.169, 18.318)</td><td>1.419 (0.160, 12.572)</td><td>0.893 (0.044, 18.122)</td></tr>
<tr><td>0.616 (0.059, 6.388)</td><td>EAR|1U|CR_NANO</td><td>0.450 (0.146, 1.382)</td><td>0.822 (0.571, 1.185)</td><td>0.495 (0.089, 2.757)</td><td>1.199 (0.593, 2.423)</td><td>0.704 (0.212, 2.340)</td><td>0.812 (0.583, 1.132)</td><td>1.185 (0.645, 2.176)</td><td>0.627 (0.078, 5.028)</td><td>1.226 (0.750, 2.004)</td><td>0.923 (0.564, 1.513)</td><td>0.528 (0.159, 1.750)</td><td>0.565 (0.370, 0.862)</td><td>0.223 (0.051, 0.980)</td><td>0.564 (0.160, 1.985)</td><td>0.625 (0.502, 0.778)</td><td>0.728 (0.186, 2.849)</td><td>0.675 (0.463, 0.985)</td><td>1.086 (0.314, 3.760)</td><td>0.653 (0.027, 15.786)</td><td>0.345 (0.044, 2.686)</td><td>3.973 (1.204, 13.106)</td><td>9.723 (1.294, 73.047)</td><td>0.699 (0.407, 1.201)</td><td>1.084 (0.842, 1.395)</td><td>0.874 (0.221, 3.457)</td><td>0.550 (0.080, 3.779)</td></tr>
<tr><td>1.369 (0.174, 10.756)</td><td>2.224 (0.724, 6.835)</td><td>EAR|2NU|CR_CH</td><td>1.829 (0.592, 5.652)</td><td>1.100 (0.295, 4.104)</td><td>2.666 (0.730, 9.734)</td><td>1.565 (0.979, 2.502)</td><td>1.807 (0.614, 5.312)</td><td>2.635 (0.752, 9.231)</td><td>1.394 (0.132, 14.692)</td><td>2.726 (0.818, 9.080)</td><td>2.054 (0.749, 5.630)</td><td>1.174 (0.734, 1.876)</td><td>1.256 (0.400, 3.946)</td><td>0.496 (0.089, 2.776)</td><td>1.253 (0.687, 2.287)</td><td>1.391 (0.456, 4.242)</td><td>1.618 (0.737, 3.551)</td><td>1.501 (0.483, 4.663)</td><td>2.416 (0.466, 12.518)</td><td>1.451 (0.050, 42.237)</td><td>0.768 (0.075, 7.877)</td><td>8.835 (2.006, 38.912)</td><td>21.622 (2.182, 214.241)</td><td>1.555 (0.471, 5.138)</td><td>2.410 (0.779, 7.460)</td><td>1.943 (0.870, 4.341)</td><td>1.223 (0.135, 11.062)</td></tr>
<tr><td>0.749 (0.072, 7.789)</td><td>1.216 (0.844, 1.752)</td><td>0.547 (0.177, 1.690)</td><td>EAR|2NU|CR_NANO</td><td>0.602 (0.108, 3.365)</td><td>1.458 (0.697, 3.046)</td><td>0.856 (0.256, 2.860)</td><td>0.988 (0.679, 1.437)</td><td>1.441 (0.739, 2.807)</td><td>0.762 (0.093, 6.239)</td><td>1.490 (0.846, 2.627)</td><td>1.123 (0.677, 1.863)</td><td>0.642 (0.193, 2.140)</td><td>0.687 (0.513, 0.919)</td><td>0.271 (0.062, 1.197)</td><td>0.685 (0.194, 2.426)</td><td>0.760 (0.540, 1.072)</td><td>0.885 (0.225, 3.481)</td><td>0.821 (0.573, 1.175)</td><td>1.321 (0.379, 4.611)</td><td>0.794 (0.032, 19.449)</td><td>0.420 (0.053, 3.334)</td><td>4.831 (1.457, 16.023)</td><td>11.824 (1.549, 90.261)</td><td>0.851 (0.555, 1.304)</td><td>1.318 (0.898, 1.935)</td><td>1.062 (0.267, 4.223)</td><td>0.669 (0.100, 4.489)</td></tr>
<tr><td>1.245 (0.117, 13.275)</td><td>2.021 (0.363, 11.260)</td><td>0.909 (0.244, 3.390)</td><td>1.662 (0.297, 9.294)</td><td>EAR|2U|CR_CH</td><td>2.422 (0.387, 15.179)</td><td>1.422 (0.406, 4.989)</td><td>1.642 (0.303, 8.891)</td><td>2.395 (0.393, 14.575)</td><td>1.267 (0.086, 18.666)</td><td>2.477 (0.421, 14.565)</td><td>1.866 (0.360, 9.673)</td><td>1.067 (0.312, 3.650)</td><td>1.141 (0.202, 6.452)</td><td>0.451 (0.052, 3.901)</td><td>1.139 (0.307, 4.222)</td><td>1.264 (0.228, 7.008)</td><td>1.471 (0.334, 6.473)</td><td>1.364 (0.243, 7.655)</td><td>2.196 (0.270, 17.871)</td><td>1.319 (0.036, 48.896)</td><td>0.698 (0.049, 10.042)</td><td>8.029 (1.118, 57.679)</td><td>19.652 (1.408, 274.321)</td><td>1.414 (0.242, 8.263)</td><td>2.191 (0.391, 12.262)</td><td>1.766 (0.397, 7.855)</td><td>1.112 (0.086, 14.339)</td></tr>
<tr><td>0.514 (0.045, 5.817)</td><td>0.834 (0.413, 1.686)</td><td>0.375 (0.103, 1.370)</td><td>0.686 (0.328, 1.434)</td><td>0.413 (0.066, 2.586)</td><td>EAR|2U|CR_NANO</td><td>0.587 (0.150, 2.297)</td><td>0.678 (0.328, 1.402)</td><td>0.988 (0.406, 2.408)</td><td>0.523 (0.059, 4.640)</td><td>1.023 (0.451, 2.319)</td><td>0.770 (0.342, 1.737)</td><td>0.440 (0.113, 1.718)</td><td>0.471 (0.220, 1.010)</td><td>0.186 (0.037, 0.936)</td><td>0.470 (0.114, 1.936)</td><td>0.522 (0.265, 1.026)</td><td>0.607 (0.134, 2.748)</td><td>0.563 (0.275, 1.153)</td><td>0.906 (0.274, 2.994)</td><td>0.544 (0.022, 13.454)</td><td>0.288 (0.033, 2.483)</td><td>3.314 (0.853, 12.878)</td><td>8.112 (1.026, 64.155)</td><td>0.584 (0.253, 1.345)</td><td>0.904 (0.442, 1.850)</td><td>0.729 (0.160, 3.331)</td><td>0.459 (0.060, 3.488)</td></tr>
<tr><td>0.875 (0.118, 6.512)</td><td>1.421 (0.427, 4.724)</td><td>0.639 (0.400, 1.021)</td><td>1.168 (0.350, 3.906)</td><td>0.703 (0.200, 2.466)</td><td>1.703 (0.435, 6.663)</td><td>EAR|3NU|CR_CH</td><td>1.154 (0.362, 3.683)</td><td>1.684 (0.448, 6.332)</td><td>0.891 (0.081, 9.756)</td><td>1.742 (0.486, 6.246)</td><td>1.312 (0.439, 3.924)</td><td>0.750 (0.585, 0.961)</td><td>0.803 (0.236, 2.724)</td><td>0.317 (0.054, 1.869)</td><td>0.801 (0.549, 1.167)</td><td>0.889 (0.269, 2.934)</td><td>1.034 (0.448, 2.388)</td><td>0.959 (0.286, 3.221)</td><td>1.544 (0.282, 8.448)</td><td>0.927 (0.031, 27.727)</td><td>0.491 (0.046, 5.233)</td><td>5.645 (1.206, 26.414)</td><td>13.816 (1.340, 142.409)</td><td>0.994 (0.279, 3.536)</td><td>1.540 (0.460, 5.155)</td><td>1.241 (0.528, 2.921)</td><td>0.782 (0.083, 7.365)</td></tr>
<tr><td>0.758 (0.075, 7.699)</td><td>1.231 (0.883, 1.715)</td><td>0.553 (0.188, 1.627)</td><td>1.012 (0.696, 1.472)</td><td>0.609 (0.112, 3.297)</td><td>1.475 (0.713, 3.051)</td><td>0.866 (0.271, 2.764)</td><td>EAR|3NU|CR_NANO</td><td>1.458 (0.762, 2.791)</td><td>0.772 (0.095, 6.280)</td><td>1.509 (0.874, 2.603)</td><td>1.137 (0.776, 1.666)</td><td>0.650 (0.204, 2.067)</td><td>0.695 (0.459, 1.052)</td><td>0.275 (0.065, 1.167)</td><td>0.694 (0.205, 2.349)</td><td>0.770 (0.568, 1.044)</td><td>0.896 (0.237, 3.382)</td><td>0.831 (0.573, 1.204)</td><td>1.337 (0.384, 4.659)</td><td>0.803 (0.033, 19.621)</td><td>0.425 (0.054, 3.355)</td><td>4.890 (1.545, 15.476)</td><td>11.968 (1.576, 90.858)</td><td>0.861 (0.502, 1.477)</td><td>1.334 (0.935, 1.904)</td><td>1.075 (0.282, 4.105)</td><td>0.677 (0.099, 4.646)</td></tr>
<tr><td>0.520 (0.047, 5.757)</td><td>0.844 (0.459, 1.550)</td><td>0.380 (0.108, 1.330)</td><td>0.694 (0.356, 1.352)</td><td>0.418 (0.069, 2.542)</td><td>1.012 (0.415, 2.464)</td><td>0.594 (0.158, 2.234)</td><td>0.686 (0.358, 1.312)</td><td>EARd|1U|CR_NANO</td><td>0.529 (0.061, 4.565)</td><td>1.034 (0.532, 2.012)</td><td>0.779 (0.370, 1.642)</td><td>0.445 (0.119, 1.671)</td><td>0.477 (0.237, 0.960)</td><td>0.188 (0.039, 0.916)</td><td>0.476 (0.120, 1.885)</td><td>0.528 (0.295, 0.944)</td><td>0.614 (0.141, 2.684)</td><td>0.570 (0.290, 1.117)</td><td>0.917 (0.236, 3.567)</td><td>0.551 (0.022, 14.002)</td><td>0.292 (0.035, 2.441)</td><td>3.353 (0.898, 12.522)</td><td>8.206 (1.017, 66.195)</td><td>0.590 (0.271, 1.284)</td><td>0.915 (0.501, 1.671)</td><td>0.737 (0.167, 3.253)</td><td>0.464 (0.062, 3.452)</td></tr>
<tr><td>0.982 (0.043, 22.323)</td><td>1.595 (0.199, 12.789)</td><td>0.717 (0.068, 7.557)</td><td>1.312 (0.160, 10.734)</td><td>0.789 (0.054, 11.623)</td><td>1.912 (0.216, 16.957)</td><td>1.122 (0.102, 12.291)</td><td>1.296 (0.159, 10.543)</td><td>1.890 (0.219, 16.302)</td><td>EARhb|1U|CR_NANO</td><td>1.955 (0.233, 16.387)</td><td>1.473 (0.175, 12.369)</td><td>0.842 (0.077, 9.205)</td><td>0.901 (0.109, 7.450)</td><td>0.356 (0.028, 4.535)</td><td>0.899 (0.080, 10.136)</td><td>0.998 (0.125, 7.951)</td><td>1.161 (0.097, 13.853)</td><td>1.076 (0.131, 8.825)</td><td>1.733 (0.155, 19.327)</td><td>1.041 (0.023, 46.516)</td><td>0.551 (0.062, 4.929)</td><td>6.336 (0.581, 69.117)</td><td>15.507 (0.865, 277.939)</td><td>1.116 (0.131, 9.477)</td><td>1.729 (0.214, 13.954)</td><td>1.393 (0.116, 16.728)</td><td>0.877 (0.052, 14.841)</td></tr>
<tr><td>0.502 (0.047, 5.423)</td><td>0.816 (0.499, 1.334)</td><td>0.367 (0.110, 1.222)</td><td>0.671 (0.381, 1.182)</td><td>0.404 (0.069, 2.373)</td><td>0.978 (0.431, 2.218)</td><td>0.574 (0.160, 2.059)</td><td>0.663 (0.384, 1.144)</td><td>0.967 (0.497, 1.880)</td><td>0.512 (0.061, 4.288)</td><td>EARm|1U|CR_NANO</td><td>0.753 (0.391, 1.453)</td><td>0.431 (0.120, 1.540)</td><td>0.461 (0.252, 0.844)</td><td>0.182 (0.039, 0.851)</td><td>0.460 (0.121, 1.741)</td><td>0.510 (0.320, 0.813)</td><td>0.594 (0.142, 2.486)</td><td>0.551 (0.310, 0.978)</td><td>0.886 (0.239, 3.293)</td><td>0.532 (0.021, 13.278)</td><td>0.282 (0.035, 2.292)</td><td>3.241 (0.910, 11.540)</td><td>7.933 (1.013, 62.141)</td><td>0.571 (0.285, 1.141)</td><td>0.884 (0.543, 1.439)</td><td>0.713 (0.169, 3.015)</td><td>0.449 (0.062, 3.235)</td></tr>
<tr><td>0.667 (0.068, 6.562)</td><td>1.083 (0.661, 1.774)</td><td>0.487 (0.178, 1.335)</td><td>0.890 (0.537, 1.477)</td><td>0.536 (0.103, 2.777)</td><td>1.298 (0.576, 2.926)</td><td>0.762 (0.255, 2.279)</td><td>0.880 (0.600, 1.289)</td><td>1.283 (0.609, 2.702)</td><td>0.679 (0.081, 5.702)</td><td>1.327 (0.688, 2.559)</td><td>RMGI</td><td>0.571 (0.192, 1.704)</td><td>0.612 (0.356, 1.051)</td><td>0.242 (0.060, 0.975)</td><td>0.610 (0.192, 1.943)</td><td>0.677 (0.421, 1.090)</td><td>0.788 (0.221, 2.813)</td><td>0.731 (0.436, 1.226)</td><td>1.176 (0.321, 4.315)</td><td>0.707 (0.028, 17.625)</td><td>0.374 (0.046, 3.048)</td><td>4.302 (1.451, 12.755)</td><td>10.529 (1.342, 82.583)</td><td>0.757 (0.399, 1.437)</td><td>1.174 (0.705, 1.954)</td><td>0.946 (0.262, 3.416)</td><td>0.596 (0.084, 4.218)</td></tr>
<tr><td>1.167 (0.154, 8.816)</td><td>1.895 (0.571, 6.283)</td><td>0.852 (0.533, 1.362)</td><td>1.558 (0.467, 5.194)</td><td>0.938 (0.274, 3.208)</td><td>2.271 (0.582, 8.864)</td><td>1.333 (1.041, 1.708)</td><td>1.539 (0.484, 4.898)</td><td>2.245 (0.598, 8.423)</td><td>1.188 (0.109, 12.992)</td><td>2.322 (0.649, 8.309)</td><td>1.750 (0.587, 5.217)</td><td>SE|1NU|CR_CH</td><td>1.070 (0.316, 3.623)</td><td>0.423 (0.072, 2.488)</td><td>1.068 (0.680, 1.676)</td><td>1.185 (0.360, 3.902)</td><td>1.379 (0.604, 3.150)</td><td>1.279 (0.382, 4.284)</td><td>2.059 (0.377, 11.245)</td><td>1.237 (0.041, 36.940)</td><td>0.655 (0.061, 6.969)</td><td>7.528 (1.612, 35.149)</td><td>18.423 (1.790, 189.635)</td><td>1.325 (0.373, 4.703)</td><td>2.054 (0.615, 6.856)</td><td>1.655 (0.711, 3.855)</td><td>1.042 (0.111, 9.807)</td></tr>
<tr><td>1.090 (0.104, 11.432)</td><td>1.771 (1.161, 2.701)</td><td>0.796 (0.253, 2.502)</td><td>1.456 (1.088, 1.950)</td><td>0.876 (0.155, 4.953)</td><td>2.122 (0.990, 4.549)</td><td>1.246 (0.367, 4.230)</td><td>1.439 (0.950, 2.177)</td><td>2.098 (1.042, 4.224)</td><td>1.110 (0.134, 9.183)</td><td>2.170 (1.185, 3.975)</td><td>1.635 (0.951, 2.811)</td><td>0.935 (0.276, 3.164)</td><td>SE|1NU|CR_NANO</td><td>0.395 (0.089, 1.765)</td><td>0.998 (0.278, 3.584)</td><td>1.107 (0.740, 1.656)</td><td>1.289 (0.323, 5.138)</td><td>1.195 (0.812, 1.758)</td><td>1.924 (0.545, 6.795)</td><td>1.156 (0.047, 28.508)</td><td>0.612 (0.076, 4.908)</td><td>7.034 (2.088, 23.693)</td><td>17.217 (2.232, 132.834)</td><td>1.238 (0.820, 1.871)</td><td>1.919 (1.236, 2.980)</td><td>1.547 (0.384, 6.233)</td><td>0.974 (0.147, 6.470)</td></tr>
<tr><td>2.758 (0.189, 40.165)</td><td>4.479 (1.020, 19.668)</td><td>2.014 (0.360, 11.262)</td><td>3.683 (0.835, 16.242)</td><td>2.216 (0.256, 19.158)</td><td>5.369 (1.068, 26.975)</td><td>3.152 (0.535, 18.571)</td><td>3.639 (0.857, 15.454)</td><td>5.307 (1.092, 25.798)</td><td>2.808 (0.221, 35.766)</td><td>5.490 (1.175, 25.651)</td><td>4.136 (1.025, 16.686)</td><td>2.364 (0.402, 13.903)</td><td>2.529 (0.566, 11.295)</td><td>SE|1U|CMP</td><td>2.524 (0.412, 15.472)</td><td>2.801 (0.642, 12.230)</td><td>3.260 (0.493, 21.535)</td><td>3.023 (0.683, 13.383)</td><td>4.866 (0.723, 32.747)</td><td>2.923 (0.088, 97.368)</td><td>1.547 (0.125, 19.217)</td><td>17.795 (3.036, 104.282)</td><td>43.550 (3.620, 524.004)</td><td>3.133 (0.675, 14.540)</td><td>4.855 (1.100, 21.432)</td><td>3.913 (0.588, 26.052)</td><td>2.464 (0.223, 27.257)</td></tr>
<tr><td>1.093 (0.152, 7.847)</td><td>1.774 (0.504, 6.250)</td><td>0.798 (0.437, 1.456)</td><td>1.459 (0.412, 5.165)</td><td>0.878 (0.237, 3.254)</td><td>2.127 (0.517, 8.756)</td><td>1.249 (0.857, 1.820)</td><td>1.442 (0.426, 4.882)</td><td>2.102 (0.530, 8.334)</td><td>1.113 (0.099, 12.547)</td><td>2.175 (0.574, 8.236)</td><td>1.639 (0.515, 5.218)</td><td>0.937 (0.597, 1.470)</td><td>1.002 (0.279, 3.600)</td><td>0.396 (0.065, 2.428)</td><td>SE|1U|CR_CH</td><td>1.110 (0.317, 3.882)</td><td>1.291 (0.516, 3.233)</td><td>1.198 (0.337, 4.259)</td><td>1.928 (0.338, 10.993)</td><td>1.158 (0.038, 35.354)</td><td>0.613 (0.056, 6.733)</td><td>7.050 (1.440, 34.512)</td><td>17.253 (1.624, 183.296)</td><td>1.241 (0.330, 4.664)</td><td>1.923 (0.543, 6.818)</td><td>1.550 (0.609, 3.948)</td><td>0.976 (0.100, 9.491)</td></tr>
<tr><td>0.985 (0.095, 10.177)</td><td>1.599 (1.285, 1.991)</td><td>0.719 (0.236, 2.193)</td><td>1.315 (0.933, 1.853)</td><td>0.791 (0.143, 4.386)</td><td>1.916 (0.975, 3.769)</td><td>1.125 (0.341, 3.715)</td><td>1.299 (0.958, 1.762)</td><td>1.895 (1.059, 3.389)</td><td>1.003 (0.126, 7.992)</td><td>1.960 (1.230, 3.123)</td><td>1.477 (0.917, 2.377)</td><td>0.844 (0.256, 2.779)</td><td>0.903 (0.604, 1.351)</td><td>0.357 (0.082, 1.559)</td><td>0.901 (0.258, 3.153)</td><td>SE|1U|CR_NANO</td><td>1.164 (0.299, 4.528)</td><td>1.079 (0.758, 1.536)</td><td>1.737 (0.508, 5.942)</td><td>1.044 (0.043, 25.191)</td><td>0.552 (0.071, 4.269)</td><td>6.352 (1.939, 20.809)</td><td>15.548 (2.093, 115.480)</td><td>1.118 (0.661, 1.891)</td><td>1.733 (1.345, 2.233)</td><td>1.397 (0.355, 5.494)</td><td>0.880 (0.129, 6.016)</td></tr>
<tr><td>0.846 (0.096, 7.445)</td><td>1.374 (0.351, 5.381)</td><td>0.618 (0.282, 1.356)</td><td>1.130 (0.287, 4.445)</td><td>0.680 (0.154, 2.992)</td><td>1.647 (0.364, 7.456)</td><td>0.967 (0.419, 2.233)</td><td>1.116 (0.296, 4.216)</td><td>1.628 (0.373, 7.114)</td><td>0.862 (0.072, 10.283)</td><td>1.684 (0.402, 7.053)</td><td>1.269 (0.355, 4.531)</td><td>0.725 (0.317, 1.657)</td><td>0.776 (0.195, 3.094)</td><td>0.307 (0.046, 2.027)</td><td>0.774 (0.309, 1.939)</td><td>0.859 (0.221, 3.344)</td><td>SE|2NU|CR_CH</td><td>0.927 (0.235, 3.664)</td><td>1.493 (0.242, 9.205)</td><td>0.897 (0.028, 28.508)</td><td>0.475 (0.041, 5.521)</td><td>5.459 (1.024, 29.105)</td><td>13.362 (1.187, 150.430)</td><td>0.961 (0.231, 3.996)</td><td>1.489 (0.378, 5.866)</td><td>1.201 (0.946, 1.523)</td><td>0.756 (0.073, 7.807)</td></tr>
<tr><td>0.912 (0.088. 9.513)</td><td>1.482 (1.016, 2.161)</td><td>0.666 (0.214, 2.070)</td><td>1.218 (0.851, 1.744)</td><td>0.733 (0.131, 4.113)</td><td>1.776 (0.867, 3.636)</td><td>1.043 (0.310, 3.502)</td><td>1.204 (0.830, 1.745)</td><td>1.755 (0.895, 3.443)</td><td>0.929 (0.113, 7.616)</td><td>1.816 (1.022, 3.225)</td><td>1.368 (0.815, 2.296)</td><td>0.782 (0.233, 2.619)</td><td>0.837 (0.569, 1.231)</td><td>0.331 (0.075, 1.464)</td><td>0.835 (0.235, 2.969)</td><td>0.927 (0.651, 1.319)</td><td>1.078 (0.273, 4.259)</td><td>SE|2NU|CR_NANO</td><td>1.610 (0.475, 5.453)</td><td>0.967 (0.039, 23.679)</td><td>0.512 (0.064, 4.070)</td><td>5.886 (1.766, 19.617)</td><td>14.405 (1.888, 109.936)</td><td>1.036 (0.615, 1.746)</td><td>1.606 (1.076, 2.396)</td><td>1.294 (0.324, 5.167)</td><td>0.815 (0.120, 5.539)</td></tr>
<tr><td>0.567 (0.041, 7.866)</td><td>0.920 (0.266, 3.186)</td><td>0.414 (0.080, 2.145)</td><td>0.757 (0.217, 2.642)</td><td>0.455 (0.056, 3.707)</td><td>1.103 (0.334, 3.644)</td><td>0.648 (0.118, 3.545)</td><td>0.748 (0.215, 2.605)</td><td>1.091 (0.280, 4.242)</td><td>0.577 (0.052, 6.438)</td><td>1.128 (0.304, 4.191)</td><td>0.850 (0.232, 3.118)</td><td>0.486 (0.089, 2.654)</td><td>0.520 (0.147, 1.836)</td><td>0.206 (0.031, 1.383)</td><td>0.519 (0.091, 2.958)</td><td>0.576 (0.168, 1.969)</td><td>0.670 (0.109, 4.130)</td><td>0.621 (0.183, 2.105)</td><td>SE|2NU|SIL</td><td>0.601 (0.020, 17.743)</td><td>0.318 (0.029, 3.454)</td><td>3.657 (0.672, 19.904)</td><td>8.950 (0.874, 91.689)</td><td>0.644 (0.174, 2.382)</td><td>0.998 (0.286, 3.477)</td><td>0.804 (0.129, 4.998)</td><td>0.506 (0.053, 4.867)</td></tr>
<tr><td>0.944 (0.018, 48.833)</td><td>1.532 (0.063, 37.066)</td><td>0.689 (0.024, 20.055)</td><td>1.260 (0.051, 30.883)</td><td>0.758 (0.020, 28.106)</td><td>1.837 (0.074, 45.386)</td><td>1.078 (0.036, 32.243)</td><td>1.245 (0.051, 30.411)</td><td>1.816 (0.071, 46.155)</td><td>0.961 (0.021, 42.936)</td><td>1.878 (0.075, 46.838)</td><td>1.415 (0.057, 35.294)</td><td>0.809 (0.027, 24.158)</td><td>0.865 (0.035, 21.351)</td><td>0.342 (0.010, 11.396)</td><td>0.864 (0.028, 26.364)</td><td>0.958 (0.040, 23.134)</td><td>1.115 (0.035, 35.446)</td><td>1.034 (0.042, 25.328)</td><td>1.665 (0.056, 49.171)</td><td>SE|2U|CR_NANO</td><td>0.529 (0.012, 23.264)</td><td>6.087 (0.204, 181.526)</td><td>14.899 (0.349, 635.874)</td><td>1.072 (0.043, 26.918)</td><td>1.661 (0.068, 40.379)</td><td>1.339 (0.042, 42.734)</td><td>0.843 (0.021, 34.654)</td></tr>
<tr><td>1.782 (0.080, 39.686)</td><td>2.894 (0.372, 22.504)</td><td>1.302 (0.127, 13.346)</td><td>2.380 (0.300, 18.891)</td><td>1.432 (0.100, 20.596)</td><td>3.469 (0.403, 29.880)</td><td>2.037 (0.191, 21.715)</td><td>2.352 (0.298, 18.556)</td><td>3.429 (0.410, 28.714)</td><td>1.815 (0.203, 16.235)</td><td>3.548 (0.436, 28.853)</td><td>2.673 (0.328, 21.780)</td><td>1.528 (0.143, 16.263)</td><td>1.635 (0.204, 13.114)</td><td>0.646 (0.052, 8.025)</td><td>1.631 (0.149, 17.914)</td><td>1.810 (0.234, 13.989)</td><td>2.106 (0.181, 24.496)</td><td>1.954 (0.246, 15.534)</td><td>3.145 (0.289, 34.155)</td><td>1.889 (0.043, 83.005)</td><td>SEhb|1U|CR_NANO</td><td>11.499 (1.083, 122.107)</td><td>28.143 (1.605, 493.341)</td><td>2.024 (0.246, 16.690)</td><td>3.137 (0.401, 24.557)</td><td>2.529 (0.216, 29.583)</td><td>1.592 (0.096, 26.330)</td></tr>
<tr><td>0.155 (0.012, 1.949)</td><td>0.252 (0.076, 0.830)</td><td>0.113 (0.026, 0.499)</td><td>0.207 (0.062, 0.687)</td><td>0.125 (0.017, 0.895)</td><td>0.302 (0.078, 1.172)</td><td>0.177 (0.038, 0.829)</td><td>0.204 (0.065, 0.647)</td><td>0.298 (0.080, 1.114)</td><td>0.158 (0.014, 1.722)</td><td>0.309 (0.087, 1.098)</td><td>0.232 (0.078, 0.689)</td><td>0.133 (0.028, 0.620)</td><td>0.142 (0.042, 0.479)</td><td>0.056 (0.010, 0.329)</td><td>0.142 (0.029, 0.694)</td><td>0.157 (0.048, 0.516)</td><td>0.183 (0.034, 0.976)</td><td>0.170 (0.051, 0.566)</td><td>0.273 (0.050, 1.488)</td><td>0.164 (0.006, 4.898)</td><td>0.087 (0.008, 0.923)</td><td>SelE|1U|CR_NANO</td><td>2.448 (0.238, 25.126)</td><td>0.176 (0.050, 0.622)</td><td>0.273 (0.082, 0.906)</td><td>0.220 (0.041, 1.182)</td><td>0.138 (0.015, 1.299)</td></tr>
</table>

0.063 (0.003, 1.375)	0.103 (0.014, 0.773)	0.046 (0.005, 0.458)	0.085 (0.011, 0.646)	0.051 (0.004, 0.710)	0.123 (0.016, 0.975)	0.072 (0.007, 0.746)	0.084 (0.011, 0.634)	0.122 (0.015, 0.983)	0.064 (0.004, 1.156)	0.126 (0.016, 0.987)	0.095 (0.012, 0.745)	0.054 (0.005, 0.559)	0.058 (0.008, 0.448)	0.023 (0.002, 0.276)	0.058 (0.005, 0.616)	0.064 (0.009, 0.478)	0.075 (0.007, 0.843)	0.069 (0.009, 0.530)	0.112 (0.011, 1.145)	0.067 (0.002, 2.865)	0.036 (0.002, 0.623)	0.409 (0.040, 4.195)	SelE|2U|CR_NANO	0.072 (0.009, 0.571)	0.111 (0.015, 0.841)	0.090 (0.008, 1.018)	0.057 (0.004, 0.909)
0.880 (0.082, 9.462)	1.430 (0.833, 2.454)	0.643 (0.195, 2.124)	1.176 (0.767, 1.803)	0.707 (0.121, 4.135)	1.714 (0.743, 3.951)	1.006 (0.283, 3.579)	1.162 (0.677, 1.993)	1.694 (0.779, 3.684)	0.896 (0.106, 7.616)	1.752 (0.877, 3.503)	1.320 (0.696, 2.506)	0.755 (0.213, 2.678)	0.807 (0.534, 1.220)	0.319 (0.069, 1.481)	0.806 (0.214, 3.027)	0.894 (0.529, 1.512)	1.040 (0.250, 4.325)	0.965 (0.573, 1.626)	1.553 (0.420, 5.747)	0.933 (0.037, 23.434)	0.494 (0.060, 4.072)	5.680 (1.608, 20.057)	13.902 (1.752, 110.278)	SelEse|1NU|CR_NANO	1.550 (0.890, 2.697)	1.249 (0.297, 5.245)	0.787 (0.118, 5.248)
0.568 (0.055, 5.913)	0.923 (0.717, 1.188)	0.415 (0.134, 1.284)	0.759 (0.517, 1.114)	0.456 (0.082, 2.555)	1.106 (0.540, 2.263)	0.649 (0.194, 2.173)	0.750 (0.525, 1.070)	1.093 (0.598, 1.997)	0.578 (0.072, 4.670)	1.131 (0.695, 1.840)	0.852 (0.512, 1.418)	0.487 (0.146, 1.625)	0.521 (0.336, 0.809)	0.206 (0.047, 0.909)	0.520 (0.147, 1.843)	0.577 (0.448, 0.743)	0.671 (0.170, 2.644)	0.623 (0.417, 0.929)	1.002 (0.288, 3.493)	0.602 (0.025, 14.639)	0.319 (0.041, 2.495)	3.665 (1.104, 12.174)	8.971 (1.189, 67.667)	0.645 (0.371, 1.123)	SelEse|1U|CR_NANO	0.806 (0.203, 3.208)	0.508 (0.074, 3.500)
0.705 (0.080, 6.246)	1.145 (0.289, 4.529)	0.515 (0.230, 1.150)	0.941 (0.237, 3.742)	0.566 (0.127, 2.519)	1.372 (0.300, 6.270)	0.805 (0.342, 1.895)	0.930 (0.244, 3.550)	1.356 (0.307, 5.983)	0.718 (0.060, 8.615)	1.403 (0.332, 5.933)	1.057 (0.293, 3.816)	0.604 (0.259, 1.407)	0.646 (0.160, 2.604)	0.256 (0.038, 1.701)	0.645 (0.253, 1.643)	0.716 (0.182, 2.815)	0.833 (0.656, 1.057)	0.773 (0.194, 3.084)	1.243 (0.200, 7.728)	0.747 (0.023, 23.843)	0.395 (0.034, 4.626)	4.547 (0.846, 24.449)	11.130 (0.983, 126.040)	0.801 (0.191, 3.362)	1.241 (0.312, 4.938)	SelEse|2NU|CR_CH	0.630 (0.061, 6.542)
1.119 (0.055, 22.703)	1.818 (0.265, 12.483)	0.817 (0.090, 7.390)	1.495 (0.223, 10.029)	0.899 (0.070, 11.596)	2.179 (0.287, 16.554)	1.279 (0.136, 12.050)	1.477 (0.215, 10.132)	2.154 (0.290, 16.010)	1.140 (0.067, 19.275)	2.228 (0.309, 16.055)	1.678 (0.237, 11.883)	0.959 (0.102, 9.024)	1.026 (0.155, 6.817)	0.406 (0.037, 4.488)	1.024 (0.105, 9.957)	1.137 (0.166, 7.773)	1.323 (0.128, 13.656)	1.227 (0.181, 8.335)	1.974 (0.205, 18.975)	1.186 (0.029, 48.755)	0.628 (0.038, 10.382)	7.220 (0.770, 67.742)	17.672 (1.100, 283.837)	1.271 (0.191, 8.482)	1.970 (0.286, 13.584)	1.588 (0.153, 16.497)	SelEse|2NU|CR_NANO

Table SF5.1. League table of relative treatment effects for retention loss.

The table presents pairwise relative risks (RRs) with 95% confidence intervals obtained from a random-effects network meta-analysis. Comparisons are interpreted as the treatment in the row relative to the treatment in the column. Relative risks below 1 indicate a reduced risk of restoration retention loss. Results correspond to the largest connected component of the network and should be interpreted in conjunction with the associated uncertainty.

EAR|1U|CR_CH	3.605 (0.067, 195.195)	1.571 (0.011, 218.372)	0.132 (0.003, 6.316)	0.252 (0.004, 16.019)	0.921 (0.017, 49.304)	0.756 (0.014, 40.342)	1.385 (0.024, 81.516)	0.417 (0.009, 18.668)	0.594 (0.012, 29.800)	9.222 (0.153, 555.962)	6.234 (0.044, 879.541)	4.078 (0.072, 229.545)	0.600 (0.012, 29.116)	0.368 (0.008, 17.733)	0.404 (0.009, 18.767)	0.050 (0.001, 4.497)	0.472 (0.009, 25.411)	0.200 (0.002, 16.821)	0.139 (0.017, 1.109)	1.245 (0.023, 66.533)	0.377 (0.008, 18.174)	0.159 (0.003, 7.764)	0.801 (0.015, 42.039)	2.797 (0.029, 267.602)	0.578 (0.010, 35.118)	1.315 (0.016, 106.155)	0.999 (0.018, 54.413)	1.067 (0.011, 100.043)	1.610 (0.018, 145.955)	2.560 (0.047, 138.310)	0.156 (0.003, 9.577)	1.327 (0.009, 195.978)
0.277 (0.005, 15.017)	EAR|1U|CR_NANO	0.436 (0.016, 12.201)	0.037 (0.011, 0.122)	0.070 (0.010, 0.508)	0.255 (0.104, 0.626)	0.210 (0.045, 0.980)	0.384 (0.135, 1.093)	0.116 (0.034, 0.389)	0.165 (0.075, 0.362)	2.558 (0.871, 7.512)	1.729 (0.089, 33.778)	1.131 (0.526, 2.433)	0.166 (0.066, 0.420)	0.102 (0.025, 0.410)	0.112 (0.036, 0.350)	0.014 (0.001, 0.192)	0.131 (0.052, 0.330)	0.055 (0.005, 0.570)	0.039 (0.001, 1.163)	0.345 (0.218, 0.548)	0.105 (0.026, 0.421)	0.044 (0.012, 0.156)	0.222 (0.099, 0.497)	0.776 (0.076, 7.930)	0.160 (0.053, 0.486)	0.365 (0.054, 2.445)	0.277 (0.074, 1.044)	0.296 (0.031, 2.818)	0.447 (0.045, 4.443)	0.710 (0.444, 1.135)	0.043 (0.007, 0.276)	0.368 (0.016, 8.580)
0.637 (0.005, 88.500)	2.295 (0.082, 64.270)	EAR|2NU|CMP	0.084 (0.003, 2.047)	0.160 (0.009, 2.941)	0.586 (0.021, 16.197)	0.482 (0.017, 13.539)	0.882 (0.028, 27.276)	0.265 (0.011, 6.158)	0.378 (0.015, 9.660)	5.871 (0.184, 186.868)	3.969 (0.047, 334.722)	2.596 (0.088, 76.165)	0.382 (0.016, 9.376)	0.234 (0.011, 5.137)	0.257 (0.011, 6.126)	0.032 (0.001, 0.938)	0.300 (0.011, 8.358)	0.127 (0.003, 5.978)	0.088 (0.001, 7.767)	0.793 (0.029, 21.848)	0.240 (0.010, 5.533)	0.101 (0.004, 2.509)	0.510 (0.019, 13.758)	1.781 (0.033, 96.902)	0.368 (0.011, 11.819)	0.837 (0.019, 37.483)	0.636 (0.023, 17.938)	0.680 (0.013, 36.122)	1.025 (0.020, 52.442)	1.630 (0.058, 45.522)	0.099 (0.003, 3.240)	0.845 (0.010, 74.963)
7.563 (0.158, 361.261)	27.268 (8.219, 90.450)	11.881 (0.488, 288.963)	EAR|2NU|CR_CH	1.905 (0.335, 10.843)	6.965 (2.181, 22.245)	5.721 (1.686, 19.416)	10.473 (2.447, 44.813)	3.150 (1.566, 6.340)	4.495 (1.806, 11.185)	69.749 (15.267, 318.620)	47.144 (1.995, 1114.098)	30.840 (8.213, 115.816)	4.534 (2.115, 9.722)	2.781 (1.027, 7.529)	3.055 (1.604, 5.819)	0.381 (0.033, 4.372)	3.569 (1.096, 11.625)	1.511 (0.156, 14.636)	1.050 (0.040, 27.371)	9.419 (2.971, 29.862)	2.854 (1.053, 7.735)	1.201 (0.750, 1.925)	6.054 (2.029, 18.069)	21.153 (1.716, 260.708)	4.374 (0.939, 20.375)	9.942 (1.114, 88.695)	7.558 (2.235, 25.554)	8.074 (0.680, 95.813)	12.176 (1.091, 135.870)	19.362 (5.877, 63.784)	1.178 (0.281, 4.936)	10.038 (0.396, 254.576)
3.970 (0.062, 252.497)	14.313 (1.969, 104.022)	6.236 (0.340, 114.388)	0.525 (0.092, 2.988)	EAR|2NU|CR_MF	3.656 (0.515, 25.974)	3.003 (0.407, 22.167)	5.498 (0.642, 47.049)	1.654 (0.312, 8.774)	2.360 (0.381, 14.622)	36.613 (4.090, 327.733)	24.749 (0.721, 848.950)	16.190 (2.062, 127.116)	2.380 (0.412, 13.758)	1.460 (0.299, 7.133)	1.604 (0.291, 8.850)	0.200 (0.036, 1.108)	1.874 (0.261, 13.468)	0.793 (0.050, 12.613)	0.551 (0.015, 20.082)	4.944 (0.699, 34.974)	1.498 (0.282, 7.957)	0.631 (0.107, 3.705)	3.178 (0.465, 21.708)	11.104 (0.571, 215.832)	2.296 (0.253, 20.832)	5.219 (0.351, 77.587)	3.967 (0.540, 29.169)	4.238 (0.225, 79.790)	6.392 (0.358, 114.263)	10.163 (1.404, 73.560)	0.619 (0.066, 5.760)	5.270 (0.144, 192.578)
1.086 (0.020, 58.131)	3.915 (1.598, 9.590)	1.706 (0.062, 47.125)	0.144 (0.045, 0.459)	0.274 (0.038, 1.943)	EAR|2NU|CR_NANO	0.821 (0.181, 3.724)	1.504 (0.448, 5.048)	0.452 (0.140, 1.467)	0.645 (0.308, 1.353)	10.014 (2.740, 36.602)	6.769 (0.317, 144.546)	4.428 (1.535, 12.775)	0.651 (0.271, 1.563)	0.399 (0.103, 1.553)	0.439 (0.146, 1.316)	0.055 (0.004, 0.739)	0.512 (0.372, 0.706)	0.217 (0.022, 2.188)	0.151 (0.005, 4.495)	1.352 (0.585, 3.125)	0.410 (0.105, 1.596)	0.172 (0.051, 0.589)	0.869 (0.437, 1.730)	3.037 (0.282, 32.671)	0.628 (0.168, 2.345)	1.427 (0.186, 10.969)	1.085 (0.298, 3.950)	1.159 (0.112, 12.037)	1.748 (0.206, 14.857)	2.780 (1.138, 6.790)	0.169 (0.027, 1.054)	1.441 (0.068, 30.338)
1.322 (0.025, 70.506)	4.766 (1.021, 22.251)	2.077 (0.074, 58.382)	0.175 (0.052, 0.593)	0.333 (0.045, 2.458)	1.217 (0.269, 5.520)	EAR|2U|CR_CH	1.831 (0.319, 10.495)	0.551 (0.172, 1.762)	0.786 (0.208, 2.969)	12.191 (2.013, 73.840)	8.241 (0.302, 225.067)	5.391 (1.046, 27.768)	0.793 (0.231, 2.717)	0.486 (0.124, 1.912)	0.534 (0.189, 1.508)	0.067 (0.005, 0.926)	0.624 (0.136, 2.872)	0.264 (0.022, 3.117)	0.184 (0.006, 5.448)	1.646 (0.365, 7.422)	0.499 (0.127, 1.960)	0.210 (0.058, 0.765)	1.058 (0.246, 4.557)	3.697 (0.251, 54.554)	0.764 (0.124, 4.707)	1.738 (0.159, 19.018)	1.321 (0.279, 6.260)	1.411 (0.099, 20.100)	2.128 (0.158, 28.628)	3.384 (0.729, 15.715)	0.206 (0.032, 1.340)	1.755 (0.060, 51.270)
0.722 (0.012, 42.513)	2.604 (0.915, 7.412)	1.134 (0.037, 35.100)	0.095 (0.022, 0.409)	0.182 (0.021, 1.557)	0.665 (0.198, 2.233)	0.546 (0.095, 3.132)	EAR|2U|CR_NANO	0.301 (0.069, 1.303)	0.429 (0.138, 1.338)	6.660 (1.676, 26.462)	4.502 (0.203, 99.614)	2.945 (0.921, 9.417)	0.433 (0.126, 1.493)	0.266 (0.053, 1.336)	0.292 (0.072, 1.188)	0.036 (0.002, 0.567)	0.341 (0.099, 1.169)	0.144 (0.012, 1.708)	0.100 (0.003, 3.340)	0.899 (0.347, 2.331)	0.273 (0.054, 1.372)	0.115 (0.025, 0.518)	0.578 (0.188, 1.776)	2.020 (0.230, 17.727)	0.418 (0.109, 1.606)	0.949 (0.117, 7.692)	0.722 (0.152, 3.435)	0.771 (0.094, 6.314)	1.163 (0.102, 13.289)	1.849 (0.661, 5.167)	0.113 (0.015, 0.854)	0.959 (0.037, 24.730)
2.401 (0.054, 107.587)	8.655 (2.571, 29.134)	3.771 (0.162, 87.558)	0.317 (0.158, 0.639)	0.605 (0.114, 3.208)	2.211 (0.682, 7.168)	1.816 (0.567, 5.812)	3.324 (0.768, 14.397)	EAR|3NU|CR_CH	1.427 (0.563, 3.619)	22.138 (4.790, 102.319)	14.965 (0.630, 355.633)	9.789 (2.573, 37.252)	1.439 (0.656, 3.157)	0.883 (0.417, 1.869)	0.970 (0.574, 1.639)	0.121 (0.011, 1.321)	1.133 (0.343, 3.745)	0.480 (0.049, 4.682)	0.333 (0.014, 8.053)	2.990 (0.929, 9.623)	0.906 (0.431, 1.906)	0.381 (0.168, 0.864)	1.922 (0.634, 5.828)	6.714 (0.541, 83.329)	1.388 (0.295, 6.542)	3.156 (0.351, 28.380)	2.399 (0.699, 8.228)	2.563 (0.214, 30.631)	3.865 (0.344, 43.441)	6.145 (1.838, 20.547)	0.374 (0.077, 1.820)	3.186 (0.125, 81.247)
1.683 (0.034, 84.361)	6.066 (2.765, 13.306)	2.643 (0.104, 67.485)	0.222 (0.089, 0.554)	0.424 (0.068, 2.626)	1.549 (0.739, 3.248)	1.273 (0.337, 4.810)	2.330 (0.747, 7.262)	0.701 (0.276, 1.778)	EAR|3NU|CR_NANO	15.516 (4.584, 52.520)	10.489 (0.507, 217.087)	6.861 (2.616, 17.993)	1.009 (0.612, 1.662)	0.619 (0.195, 1.958)	0.680 (0.296, 1.559)	0.085 (0.007, 1.034)	0.794 (0.368, 1.714)	0.336 (0.037, 3.023)	0.234 (0.008, 6.449)	2.096 (1.026, 4.281)	0.635 (0.200, 2.013)	0.267 (0.099, 0.723)	1.347 (0.729, 2.489)	4.706 (0.452, 48.984)	0.973 (0.281, 3.374)	2.212 (0.302, 16.213)	1.681 (0.575, 4.917)	1.796 (0.180, 17.950)	2.709 (0.289, 25.429)	4.307 (1.985, 9.346)	0.262 (0.049, 1.409)	2.233 (0.100, 49.874)
0.108 (0.002, 6.537)	0.391 (0.133, 1.148)	0.170 (0.005, 5.422)	0.014 (0.003, 0.066)	0.027 (0.003, 0.244)	0.100 (0.027, 0.365)	0.082 (0.014, 0.497)	0.150 (0.038, 0.597)	0.045 (0.010, 0.209)	0.064 (0.019, 0.218)	EARd|1U|CR_NANO	0.676 (0.030, 15.174)	0.442 (0.140, 1.398)	0.065 (0.017, 0.242)	0.040 (0.007, 0.213)	0.044 (0.010, 0.191)	0.005 (0.000, 0.088)	0.051 (0.014, 0.191)	0.022 (0.002, 0.267)	0.015 (0.000, 0.516)	0.135 (0.050, 0.367)	0.041 (0.008, 0.219)	0.017 (0.004, 0.083)	0.087 (0.025, 0.299)	0.303 (0.025, 3.679)	0.063 (0.015, 0.263)	0.143 (0.017, 1.180)	0.108 (0.021, 0.548)	0.116 (0.010, 1.308)	0.175 (0.015, 2.087)	0.278 (0.099, 0.776)	0.017 (0.002, 0.135)	0.144 (0.005, 3.845)
0.160 (0.001, 22.635)	0.578 (0.030, 11.298)	0.252 (0.003, 21.253)	0.021 (0.001, 0.501)	0.040 (0.001, 1.386)	0.148 (0.007, 3.155)	0.121 (0.004, 3.314)	0.222 (0.010, 4.915)	0.067 (0.003, 1.588)	0.095 (0.005, 1.973)	1.479 (0.066, 33.208)	EARhb|1U|CR_NANO	0.654 (0.032, 13.415)	0.096 (0.004, 2.071)	0.059 (0.002, 1.506)	0.065 (0.003, 1.497)	0.008 (0.000, 0.411)	0.076 (0.004, 1.631)	0.032 (0.001, 1.351)	0.022 (0.000, 1.989)	0.200 (0.010, 3.807)	0.061 (0.002, 1.547)	0.025 (0.001, 0.617)	0.128 (0.006, 2.676)	0.449 (0.011, 18.711)	0.093 (0.004, 2.101)	0.211 (0.009, 4.856)	0.160 (0.006, 3.984)	0.171 (0.004, 6.811)	0.258 (0.006, 10.671)	0.411 (0.021, 8.012)	0.025 (0.001, 0.798)	0.213 (0.003, 15.680)
0.245 (0.004, 13.805)	0.884 (0.411, 1.901)	0.385 (0.013, 11.301)	0.032 (0.009, 0.122)	0.062 (0.008, 0.485)	0.226 (0.078, 0.652)	0.186 (0.036, 0.956)	0.340 (0.106, 1.086)	0.102 (0.027, 0.389)	0.146 (0.056, 0.382)	2.261 (0.715, 7.149)	1.529 (0.075, 31.350)	EARm|1U|CR_NANO	0.147 (0.050, 0.434)	0.090 (0.020, 0.404)	0.099 (0.028, 0.352)	0.012 (0.001, 0.180)	0.116 (0.039, 0.343)	0.049 (0.004, 0.538)	0.034 (0.001, 1.077)	0.305 (0.156, 0.597)	0.093 (0.021, 0.415)	0.039 (0.010, 0.155)	0.196 (0.073, 0.526)	0.686 (0.063, 7.428)	0.142 (0.042, 0.482)	0.322 (0.045, 2.330)	0.245 (0.058, 1.034)	0.262 (0.026, 2.633)	0.395 (0.037, 4.207)	0.628 (0.311, 1.267)	0.038 (0.006, 0.265)	0.325 (0.013, 7.979)
1.668 (0.034, 80.996)	6.013 (2.383, 15.170)	2.620 (0.107, 64.354)	0.221 (0.103, 0.473)	0.420 (0.073, 2.428)	1.536 (0.640, 3.687)	1.262 (0.368, 4.325)	2.309 (0.670, 7.961)	0.695 (0.317, 1.524)	0.991 (0.602, 1.633)	15.380 (4.134, 57.226)	10.397 (0.483, 223.811)	6.801 (2.307, 20.053)	RMGI	0.613 (0.217, 1.732)	0.674 (0.347, 1.308)	0.084 (0.007, 0.975)	0.787 (0.320, 1.939)	0.333 (0.039, 2.829)	0.232 (0.009, 6.155)	2.077 (0.874, 4.938)	0.629 (0.222, 1.781)	0.265 (0.112, 0.627)	1.335 (0.610, 2.923)	4.665 (0.426, 51.060)	0.964 (0.253, 3.670)	2.193 (0.282, 17.053)	1.667 (0.645, 4.309)	1.781 (0.169, 18.730)	2.685 (0.272, 26.475)	4.270 (1.708, 10.676)	0.260 (0.052, 1.294)	2.214 (0.096, 51.244)
2.719 (0.056, 131.145)	9.804 (2.440, 39.397)	4.272 (0.195, 93.738)	0.360 (0.133, 0.974)	0.685 (0.140, 3.347)	2.505 (0.644, 9.741)	2.057 (0.523, 8.091)	3.766 (0.749, 18.942)	1.133 (0.535, 2.398)	1.616 (0.511, 5.116)	25.078 (4.699, 133.847)	16.952 (0.664, 432.940)	11.090 (2.477, 49.655)	1.631 (0.577, 4.605)	SE|1NU|CMP	1.098 (0.450, 2.683)	0.137 (0.013, 1.414)	1.283 (0.324, 5.076)	0.544 (0.050, 5.856)	0.378 (0.014, 9.953)	3.387 (0.876, 13.091)	1.026 (0.555, 1.897)	0.432 (0.147, 1.268)	2.177 (0.593, 7.994)	7.606 (0.560, 103.286)	1.573 (0.289, 8.543)	3.575 (0.359, 35.623)	2.717 (0.665, 11.102)	2.903 (0.222, 38.012)	4.378 (0.355, 54.039)	6.962 (1.743, 27.807)	0.424 (0.075, 2.393)	3.610 (0.132, 98.761)
2.476 (0.053, 115.031)	8.926 (2.859, 27.874)	3.889 (0.163, 92.657)	0.327 (0.172, 0.624)	0.624 (0.113, 3.441)	2.280 (0.760, 6.840)	1.873 (0.663, 5.289)	3.428 (0.842, 13.961)	1.031 (0.610, 1.743)	1.472 (0.641, 3.376)	22.833 (5.239, 99.494)	15.434 (0.668, 356.630)	10.097 (2.839, 35.902)	1.484 (0.765, 2.882)	0.910 (0.373, 2.224)	SE|1NU|CR_CH	0.125 (0.011, 1.400)	1.168 (0.381, 3.579)	0.495 (0.053, 4.644)	0.344 (0.014, 8.670)	3.083 (1.036, 9.179)	0.934 (0.383, 2.278)	0.393 (0.182, 0.850)	1.982 (0.710, 5.533)	6.925 (0.578, 82.963)	1.432 (0.322, 6.366)	3.255 (0.377, 28.106)	2.474 (0.777, 7.882)	2.643 (0.229, 30.475)	3.986 (0.368, 43.185)	6.338 (2.045, 19.648)	0.386 (0.081, 1.833)	3.286 (0.132, 81.532)
19.850 (0.222, 1772.064)	71.565 (5.210, 983.089)	31.181 (1.066, 911.781)	2.625 (0.229, 30.123)	5.000 (0.903, 27.699)	18.280 (1.354, 246.855)	15.016 (1.080, 208.700)	27.487 (1.764, 428.247)	8.269 (0.757, 90.306)	11.798 (0.967, 143.955)	183.057 (11.344, 2954.250)	123.742 (2.436, 6286.753)	80.947 (5.555, 1179.564)	11.902 (1.026, 138.089)	7.299 (0.707, 75.317)	8.017 (0.714, 90.017)	SE|1NU|CR_MF	9.368 (0.688, 127.625)	3.967 (0.153, 102.617)	2.756 (0.051, 147.821)	24.722 (1.837, 332.753)	7.490 (0.685, 81.867)	3.153 (0.269, 37.018)	15.892 (1.212, 208.325)	55.518 (1.806, 1706.842)	11.479 (0.704, 187.242)	26.096 (1.068, 637.784)	19.836 (1.431, 274.898)	21.189 (0.709, 633.715)	31.960 (1.118, 914.063)	50.815 (3.711, 695.826)	3.093 (0.186, 51.496)	26.348 (0.490, 1417.146)

2.119 (0.039, 114.091)	7.639 (3.026, 19.284)	3.328 (0.120, 92.592)	0.280 (0.086, 0.913)	0.534 (0.074, 3.836)	1.951 (1.416, 2.688)	1.603 (0.348, 7.378)	2.934 (0.855, 10.064)	0.883 (0.267, 2.918)	1.259 (0.583, 2.718)	19.541 (5.235, 72.937)	13.209 (0.613, 284.604)	8.641 (2.919, 25.575)	1.270 (0.516, 3.130)	0.779 (0.197, 3.082)	0.856 (0.279, 2.621)	0.107 (0.008, 1.454)	SE|1NU|CR_NANO	0.424 (0.042, 4.312)	0.294 (0.010, 8.831)	2.639 (1.106, 6.297)	0.800 (0.202, 3.167)	0.337 (0.097, 1.170)	1.696 (0.827, 3.480)	5.926 (0.545, 64.425)	1.225 (0.321, 4.671)	2.785 (0.358, 21.693)	2.117 (0.571, 7.845)	2.262 (0.215, 23.758)	3.412 (0.405, 28.772)	5.424 (2.155, 13.655)	0.330 (0.052, 2.082)	2.813 (0.135, 58.610)
5.004 (0.059, 421.112)	18.038 (1.755, 185.434)	7.859 (0.167, 369.260)	0.662 (0.068, 6.406)	1.260 (0.079, 20.033)	4.608 (0.457, 46.460)	3.785 (0.321, 44.661)	6.928 (0.586, 81.974)	2.084 (0.214, 20.340)	2.974 (0.331, 26.733)	46.141 (3.751, 567.704)	31.190 (0.740, 1314.090)	20.403 (1.858, 224.079)	3.000 (0.353, 25.460)	1.840 (0.171, 19.824)	2.021 (0.215, 18.965)	0.252 (0.010, 6.520)	2.361 (0.232, 24.047)	SE|1U|CMP	0.695 (0.014, 34.862)	6.231 (0.620, 62.602)	1.888 (0.175, 20.361)	0.795 (0.079, 7.969)	4.006 (0.411, 39.064)	13.995 (0.565, 346.541)	2.894 (0.232, 36.025)	6.578 (0.340, 127.345)	5.000 (0.482, 51.904)	5.341 (0.222, 128.419)	8.056 (0.351, 184.657)	12.808 (1.251, 131.197)	0.780 (0.054, 11.304)	6.641 (0.148, 297.080)
7.202 (0.902, 57.524)	25.964 (0.860, 784.385)	11.312 (0.129, 994.062)	0.952 (0.037, 24.819)	1.814 (0.050, 66.082)	6.633 (0.222, 197.730)	5.448 (0.184, 161.693)	9.972 (0.299, 332.154)	3.000 (0.124, 72.479)	4.280 (0.155, 118.144)	66.413 (1.940, 2274.237)	44.894 (0.503, 4009.813)	29.368 (0.929, 928.528)	4.318 (0.162, 114.767)	2.648 (0.100, 69.805)	2.909 (0.115, 73.362)	0.363 (0.007, 19.459)	3.399 (0.113, 102.023)	1.439 (0.029, 72.226)	SE|1U|CR_CH	8.969 (0.302, 266.747)	2.717 (0.103, 71.529)	1.144 (0.043, 30.643)	5.766 (0.198, 168.040)	20.142 (0.347, 1168.061)	4.165 (0.121, 143.811)	9.468 (0.198, 453.275)	7.197 (0.237, 218.875)	7.688 (0.136, 435.546)	11.595 (0.212, 632.766)	18.436 (0.612, 555.629)	1.122 (0.032, 39.299)	9.559 (0.102, 897.488)
0.803 (0.015, 42.897)	2.895 (1.826, 4.589)	1.261 (0.046, 34.758)	0.106 (0.033, 0.337)	0.202 (0.029, 1.431)	0.739 (0.320, 1.709)	0.607 (0.135, 2.738)	1.112 (0.429, 2.882)	0.334 (0.104, 1.077)	0.477 (0.234, 0.975)	7.405 (2.723, 20.136)	5.005 (0.263, 95.383)	3.274 (1.674, 6.405)	0.481 (0.203, 1.144)	0.295 (0.076, 1.141)	0.324 (0.109, 0.966)	0.040 (0.003, 0.544)	0.379 (0.159, 0.904)	0.160 (0.016, 1.612)	0.111 (0.004, 3.316)	SE|1U|CR_NANO	0.303 (0.078, 1.173)	0.128 (0.038, 0.432)	0.643 (0.306, 1.353)	2.246 (0.228, 22.145)	0.464 (0.166, 1.297)	1.056 (0.164, 6.807)	0.802 (0.222, 2.901)	0.857 (0.094, 7.811)	1.293 (0.133, 12.582)	2.055 (1.372, 3.080)	0.125 (0.020, 0.775)	1.066 (0.046, 24.444)
2.650 (0.055, 127.651)	9.554 (2.374, 38.448)	4.163 (0.181, 95.890)	0.350 (0.129, 0.950)	0.668 (0.126, 3.546)	2.440 (0.627, 9.507)	2.005 (0.510, 7.876)	3.670 (0.729, 18.484)	1.104 (0.525, 2.323)	1.575 (0.497, 4.994)	24.439 (4.574, 130.595)	16.520 (0.646, 422.167)	10.807 (2.410, 48.453)	1.589 (0.561, 4.497)	0.975 (0.527, 1.801)	1.070 (0.439, 2.610)	0.134 (0.012, 1.459)	1.251 (0.316, 4.955)	0.530 (0.049, 5.712)	0.368 (0.014, 9.686)	3.301 (0.853, 12.776)	SE|2NU|CMP	0.421 (0.143, 1.239)	2.122 (0.577, 7.803)	7.412 (0.545, 100.736)	1.532 (0.282, 8.335)	3.484 (0.349, 34.747)	2.648 (0.647, 10.834)	2.829 (0.216, 37.073)	4.267 (0.345, 52.699)	6.784 (1.696, 27.140)	0.413 (0.073, 2.335)	3.518 (0.128, 96.293)
6.296 (0.129, 307.754)	22.699 (6.414, 80.327)	9.890 (0.399, 245.403)	0.832 (0.520, 1.334)	1.586 (0.270, 9.318)	5.798 (1.698, 19.792)	4.763 (1.307, 17.357)	8.718 (1.931, 39.366)	2.623 (1.158, 5.939)	3.742 (1.383, 10.122)	58.061 (12.071, 279.276)	39.248 (1.620, 950.987)	25.675 (6.446, 102.258)	3.775 (1.596, 8.927)	2.315 (0.788, 6.798)	2.543 (1.176, 5.498)	0.317 (0.027, 3.724)	2.971 (0.854, 10.334)	1.258 (0.125, 12.616)	0.874 (0.033, 23.418)	7.841 (2.313, 26.584)	2.376 (0.807, 6.994)	SE|2NU|CR_CH	5.040 (1.574, 16.140)	17.609 (1.384, 223.967)	3.641 (0.743, 17.846)	8.276 (0.895, 76.547)	6.292 (1.746, 22.671)	6.721 (0.548, 82.352)	10.136 (0.879, 116.874)	16.117 (4.584, 56.662)	0.981 (0.249, 3.871)	8.357 (0.322, 217.174)
1.249 (0.024, 65.602)	4.504 (2.011, 10.087)	1.962 (0.073, 52.974)	0.165 (0.055, 0.493)	0.315 (0.046, 2.149)	1.150 (0.578, 2.289)	0.945 (0.219, 4.069)	1.730 (0.563, 5.314)	0.520 (0.172, 1.578)	0.742 (0.402, 1.372)	11.520 (3.343, 39.698)	7.787 (0.374, 162.276)	5.094 (1.900, 13.660)	0.749 (0.342, 1.640)	0.459 (0.125, 1.687)	0.505 (0.181, 1.409)	0.063 (0.005, 0.825)	0.590 (0.287, 1.209)	0.250 (0.026, 2.435)	0.173 (0.006, 5.056)	1.556 (0.739, 3.273)	0.471 (0.128, 1.734)	0.198 (0.062, 0.635)	SE|2NU|CR_NANO	3.494 (0.344, 35.474)	0.722 (0.205, 2.542)	1.642 (0.222, 12.163)	1.248 (0.364, 4.276)	1.333 (0.134, 13.285)	2.011 (0.220, 18.416)	3.198 (1.431, 7.143)	0.195 (0.033, 1.162)	1.658 (0.076, 36.191)
0.358 (0.004, 34.209)	1.289 (0.126, 13.176)	0.562 (0.010, 30.566)	0.047 (0.004, 0.583)	0.090 (0.005, 1.750)	0.329 (0.031, 3.542)	0.270 (0.018, 3.991)	0.495 (0.056, 4.345)	0.149 (0.012, 1.849)	0.213 (0.020, 2.212)	3.297 (0.272, 40.001)	2.229 (0.053, 92.954)	1.458 (0.135, 15.790)	0.214 (0.020, 2.347)	0.131 (0.010, 1.785)	0.144 (0.012, 1.730)	0.018 (0.001, 0.554)	0.169 (0.016, 1.834)	0.071 (0.003, 1.770)	0.050 (0.001, 2.879)	0.445 (0.045, 4.391)	0.135 (0.010, 1.833)	0.057 (0.004, 0.722)	0.286 (0.028, 2.906)	SE|2NU|SIL	0.207 (0.017, 2.481)	0.470 (0.025, 8.983)	0.357 (0.027, 4.690)	0.382 (0.019, 7.724)	0.576 (0.024, 13.821)	0.915 (0.090, 9.295)	0.056 (0.003, 0.994)	0.475 (0.010, 22.023)
1.729 (0.028, 105.015)	6.235 (2.056, 18.906)	2.716 (0.085, 87.208)	0.229 (0.049, 1.065)	0.436 (0.048, 3.952)	1.592 (0.426, 5.947)	1.308 (0.212, 8.055)	2.395 (0.623, 9.208)	0.720 (0.153, 3.394)	1.028 (0.296, 3.564)	15.947 (3.808, 66.780)	10.780 (0.476, 244.203)	7.052 (2.073, 23.984)	1.037 (0.272, 3.946)	0.636 (0.117, 3.454)	0.698 (0.157, 3.106)	0.087 (0.005, 1.421)	0.816 (0.214, 3.111)	0.346 (0.028, 4.303)	0.240 (0.007, 8.292)	2.154 (0.771, 6.015)	0.653 (0.120, 3.549)	0.275 (0.056, 1.346)	1.384 (0.393, 4.871)	4.837 (0.403, 58.027)	SE|2U|CR_NANO	2.273 (0.271, 19.068)	1.728 (0.335, 8.905)	1.846 (0.165, 20.629)	2.784 (0.230, 33.656)	4.427 (1.477, 13.267)	0.269 (0.033, 2.176)	2.295 (0.085, 61.831)
0.761 (0.009, 61.424)	2.743 (0.409, 18.388)	1.195 (0.027, 53.522)	0.101 (0.011, 0.897)	0.192 (0.013, 2.849)	0.701 (0.091, 5.383)	0.575 (0.053, 6.298)	1.053 (0.130, 8.535)	0.317 (0.035, 2.850)	0.452 (0.062, 3.314)	7.015 (0.848, 58.061)	4.742 (0.206, 109.202)	3.102 (0.429, 22.421)	0.456 (0.059, 3.547)	0.280 (0.028, 2.787)	0.307 (0.036, 2.653)	0.038 (0.002, 0.937)	0.359 (0.046, 2.796)	0.152 (0.008, 2.943)	0.106 (0.002, 5.057)	0.947 (0.147, 6.109)	0.287 (0.029, 2.863)	0.121 (0.013, 1.117)	0.609 (0.082, 4.510)	2.128 (0.111, 40.662)	0.440 (0.052, 3.690)	SEhb|1U|CR_NANO	0.760 (0.079, 7.289)	0.812 (0.045, 14.614)	1.225 (0.065, 23.132)	1.947 (0.291, 13.032)	0.119 (0.009, 1.604)	1.010 (0.026, 38.571)
1.001 (0.018, 54.495)	3.608 (0.958, 13.590)	1.572 (0.056, 44.323)	0.132 (0.039, 0.447)	0.252 (0.034, 1.853)	0.922 (0.253, 3.354)	0.757 (0.160, 3.587)	1.386 (0.291, 6.596)	0.417 (0.122, 1.430)	0.595 (0.203, 1.740)	9.229 (1.824, 46.693)	6.238 (0.251, 155.027)	4.081 (0.968, 17.212)	0.600 (0.232, 1.551)	0.368 (0.090, 1.503)	0.404 (0.127, 1.288)	0.050 (0.004, 0.699)	0.472 (0.127, 1.750)	0.200 (0.019, 2.076)	0.139 (0.005, 4.226)	1.246 (0.345, 4.507)	0.378 (0.092, 1.545)	0.159 (0.044, 0.573)	0.801 (0.234, 2.745)	2.799 (0.213, 36.741)	0.579 (0.112, 2.982)	1.315 (0.137, 12.613)	SelE|1U|CR_NANO	1.068 (0.084, 13.515)	1.611 (0.135, 19.196)	2.562 (0.684, 9.590)	0.156 (0.024, 1.007)	1.328 (0.050, 35.385)
0.937 (0.010, 87.795)	3.377 (0.355, 32.140)	1.472 (0.028, 78.210)	0.124 (0.010, 1.470)	0.236 (0.013, 4.442)	0.863 (0.083, 8.958)	0.709 (0.050, 10.094)	1.297 (0.158, 10.625)	0.390 (0.033, 4.664)	0.557 (0.056, 5.564)	8.639 (0.764, 97.622)	5.840 (0.147, 232.293)	3.820 (0.380, 38.417)	0.562 (0.053, 5.909)	0.344 (0.026, 4.510)	0.378 (0.033, 4.363)	0.047 (0.002, 1.411)	0.442 (0.042, 4.644)	0.187 (0.008, 4.501)	0.130 (0.002, 7.369)	1.167 (0.128, 10.631)	0.353 (0.027, 4.632)	0.149 (0.012, 1.823)	0.750 (0.075, 7.472)	2.620 (0.129, 53.022)	0.542 (0.048, 6.054)	1.231 (0.068, 22.165)	0.936 (0.074, 11.844)	SelE|2U|CR_NANO	1.508 (0.064, 35.332)	2.398 (0.254, 22.617)	0.146 (0.008, 2.520)	1.243 (0.027, 56.605)
0.621 (0.007, 56.306)	2.239 (0.225, 22.278)	0.976 (0.019, 49.919)	0.082 (0.007, 0.916)	0.156 (0.009, 2.797)	0.572 (0.067, 4.861)	0.470 (0.035, 6.320)	0.860 (0.075, 9.830)	0.259 (0.023, 2.908)	0.369 (0.039, 3.465)	5.728 (0.479, 68.477)	3.872 (0.094, 159.956)	2.533 (0.238, 26.991)	0.372 (0.038, 3.671)	0.228 (0.019, 2.819)	0.251 (0.023, 2.718)	0.031 (0.001, 0.895)	0.293 (0.035, 2.472)	0.124 (0.005, 2.845)	0.086 (0.002, 4.706)	0.774 (0.079, 7.529)	0.234 (0.019, 2.895)	0.099 (0.009, 1.137)	0.497 (0.054, 4.553)	1.737 (0.072, 41.708)	0.359 (0.030, 4.342)	0.817 (0.043, 15.421)	0.621 (0.052, 7.395)	0.663 (0.028, 15.534)	SelEse|1NU|CR_NANO	1.590 (0.160, 15.801)	0.097 (0.006, 1.584)	0.824 (0.026, 26.191)
0.391 (0.007, 21.105)	1.408 (0.881, 2.252)	0.614 (0.022, 17.140)	0.052 (0.016, 0.170)	0.098 (0.014, 0.712)	0.360 (0.147, 0.879)	0.295 (0.064, 1.372)	0.541 (0.194, 1.512)	0.163 (0.049, 0.544)	0.232 (0.107, 0.504)	3.602 (1.288, 10.075)	2.435 (0.125, 47.508)	1.593 (0.789, 3.214)	0.234 (0.094, 0.586)	0.144 (0.036, 0.574)	0.158 (0.051, 0.489)	0.020 (0.001, 0.269)	0.184 (0.073, 0.464)	0.078 (0.008, 0.800)	0.054 (0.002, 1.635)	0.487 (0.325, 0.729)	0.147 (0.037, 0.590)	0.062 (0.018, 0.218)	0.313 (0.140, 0.699)	1.093 (0.108, 11.095)	0.226 (0.075, 0.677)	0.514 (0.077, 3.437)	0.390 (0.104, 1.461)	0.417 (0.044, 3.933)	0.629 (0.063, 6.250)	SelEse|1U|CR_NANO	0.061 (0.010, 0.387)	0.519 (0.022, 12.073)
6.419 (0.104, 394.571)	23.141 (3.627, 147.658)	10.082 (0.309, 329.343)	0.849 (0.203, 3.555)	1.617 (0.174, 15.058)	5.911 (0.949, 36.811)	4.855 (0.746, 31.595)	8.888 (1.170, 67.498)	2.674 (0.550, 13.010)	3.815 (0.710, 20.502)	59.193 (7.434, 471.350)	40.013 (1.253, 1278.062)	26.175 (3.777, 181.399)	3.849 (0.773, 19.171)	2.360 (0.418, 13.328)	2.592 (0.545, 12.322)	0.323 (0.019, 5.384)	3.029 (0.480, 19.104)	1.283 (0.088, 18.603)	0.891 (0.025, 31.215)	7.994 (1.290, 49.551)	2.422 (0.428, 13.697)	1.019 (0.258, 4.024)	5.139 (0.861, 30.677)	17.952 (1.006, 320.410)	3.712 (0.460, 29.985)	8.438 (0.624, 114.171)	6.414 (0.993, 41.438)	6.852 (0.397, 118.333)	10.335 (0.631, 169.203)	16.431 (2.586, 104.386)	SelEse|2NU|CR_CH	8.520 (0.250, 290.296)
0.753 (0.005, 111.230)	2.716 (0.117, 63.301)	1.183 (0.013, 104.994)	0.100 (0.004, 2.526)	0.190 (0.005, 6.935)	0.694 (0.033, 14.604)	0.570 (0.020, 16.653)	1.043 (0.040, 26.915)	0.314 (0.012, 8.002)	0.448 (0.020, 9.999)	6.948 (0.260, 185.583)	4.696 (0.064, 345.883)	3.072 (0.125, 75.309)	0.452 (0.020, 10.456)	0.277 (0.010, 7.579)	0.304 (0.012, 7.550)	0.038 (0.001, 2.041)	0.356 (0.017, 7.410)	0.151 (0.003, 6.735)	0.105 (0.001, 9.822)	0.938 (0.041, 21.520)	0.284 (0.010, 7.783)	0.120 (0.005, 3.110)	0.603 (0.028, 13.164)	2.107 (0.045, 97.778)	0.436 (0.016, 11.735)	0.990 (0.026, 37.834)	0.753 (0.028, 20.055)	0.804 (0.018, 36.609)	1.213 (0.038, 38.536)	1.929 (0.083, 44.912)	0.117 (0.003, 3.999)	SelEse|2NU|CR_NANO
